# Click. Screen. Degrade. A Miniaturized D2B Workflow for Rapid PROTAC Discovery

**DOI:** 10.1021/acs.jmedchem.5c02543

**Published:** 2026-01-23

**Authors:** Marko Mitrović, Francesco Aleksy Greco, Yiliam Cruz García, Aleksandar Lučić, Lasse Hoffmann, Rohit Chander, Julia Schönfeld, Nick Liebisch, Saran Aswathaman Sivashanmugam, Martin Peter Schwalm, Markus Egner, Max Lewandowski, Daniel Merk, Viktoria Morasch, Elmar Wolf, Susanne Müller, Thomas Hanke, Ewgenij Proschak, Kerstin Hiesinger, Stefan Knapp

**Affiliations:** † Institute of Pharmaceutical Chemistry, 9173 Goethe University Frankfurt, Max-von-Laue-Str. 9, Frankfurt am Main 60438, Germany; ‡ Structural Genomics Consortium (SGC), Buchmann Institute for Molecular Life Sciences (BMLS), Max-von-Laue-Str. 15, Frankfurt am Main 60438, Germany; § German Cancer Research Center (DKFZ), Im Neuenheimer Feld 280, Heidelberg 69120, Germany; ∥ Institute of Biochemistry, University of Kiel, Rudolf-Höber-Str. 1, Kiel 24118, Germany; ⊥ Department of Pharmacy, Ludwig-Maximilians-Universität (LMU) München, Munich 81377, Germany; # Fraunhofer Institute for Translational Medicine and Pharmacology (ITMP), Theodor-Stern-Kai 7, Frankfurt am Main 60596, Germany

## Abstract

Targeted protein degradation is one of the fastest developing fields in medicinal chemistry and chemical biology. Despite significant development in assay technologies and inhibitor discovery, the development of PROTACs remains a challenging endeavor since rational design approaches remain widely elusive. Our workflow eliminates the rate-limiting step of classic synthesis, namely compound purification, and pairs it with high-throughput, semi-automated plate-based synthesis, and direct cellular assay evaluation. We applied this direct-to-biology approach to four diverse targets, demonstrating the general applicability of this technology. PROTAC synthesis was realized by using the highly efficient copper-catalyzed azide–alkyne cycloaddition reaction. This simplified reaction setup enabled synthesis in the nanomole scale with reaction volumes as low as 5 μL. The high-throughput strategy allows hundreds of PROTACs to be synthesized and evaluated within a few days, facilitating comprehensive assessment of target degradability, rapid hit identification, and selection of the most suitable E3 ligase for degrader development.

## Graphical abstract

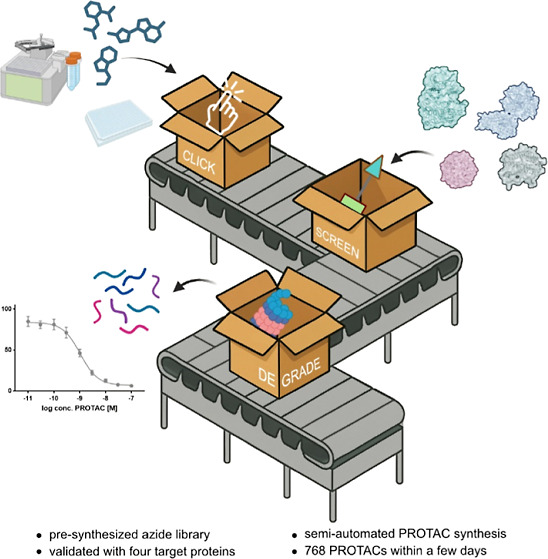

## Introduction

Targeted protein degradation (TPD) has emerged as a new and promising, and potentially transformative pharmacological modality, receiving increased focus from researchers in academia and industry. Since the first reports of Proteolysis Targeting Chimeras (PROTACs) in 2001, more than 30 degrader molecules have entered clinical development for diverse oncology indications.^1–3^ The general architecture of a heterobifunctional PROTAC molecule comprises a ligand that targets a given E3 ligase and a warhead that binds to the protein of interest (POI) connected via a linker region. A ternary complex is formed between the PROTAC, the POI and the E3 ligase, which is poised to catalyze the transfer of ubiquitin onto the POI, thereby tagging it for subsequent degradation by the ubiquitin-proteasome system.^4,5^ Unlike conventional small molecule inhibitors, which rely on druggable binding pockets and require high inhibitor occupancy at the targeted binding site, degraders function catalytically via an event-driven mode of action. Additionally, the ligands used in the development of PROTACs do not need to bind to sites relevant to the disease-causing process of the POI. This has greatly expanded the druggable proteome.^6^

However, one of the main challenges for the development of PROTACs is the lack of rational design strategies. Furthermore, due to the increased structural complexity associated with this class of molecules, the synthesis and purification of the compounds remain the rate-limiting steps in the development of degraders (Figure 1A). Although critical steps for PROTAC activity have been highlighted,^7^ such as the stability of the ternary complex consisting of the PROTAC-mediated POI and the E3 complex, the design of PROTACs predominantly relies on combinatorial approaches that optimize linker properties and attachment points. This approach renders the development of PROTACs to be very time-consuming and resource-intensive. Thus, the development of PROTACs would benefit from methods that would allow for more streamlined approaches which reduce the time from synthesis to biological evaluation. Direct-to-biology (D2B) has emerged as a promising strategy that eliminates the need for purification by directly testing crude reaction mixtures in relevant assay systems.^8–11^ Using this strategy, traceless coupling to generate phthalimidine-linked PROTACs,^12^ amide coupling^13^ to join POI and E3 ligands, light-induced cyclization to form indazolone-linked PROTACs,^14^ Selenium–Nitrogen Exchange (SeNEx) Click Chemistry^15^ and other miniaturized reactions have yielded libraries ranging from about 40 to over 600 compounds targeting proteins such as BRD4, the androgen receptor, and CDK9 (cyclin-dependent kinase 9). GSK further expanded D2B by adapting multiple reaction types (reductive amination, nucleophilic aromatic substitution, alkylation and C­(sp^2^)–C­(sp^3^) cross-coupling) to 1536-well formats.^16^ Beyond PROTAC discovery, D2B has also been applied to nanoscale synthesis and screening of molecular glues.^17^

**Figure 1:**
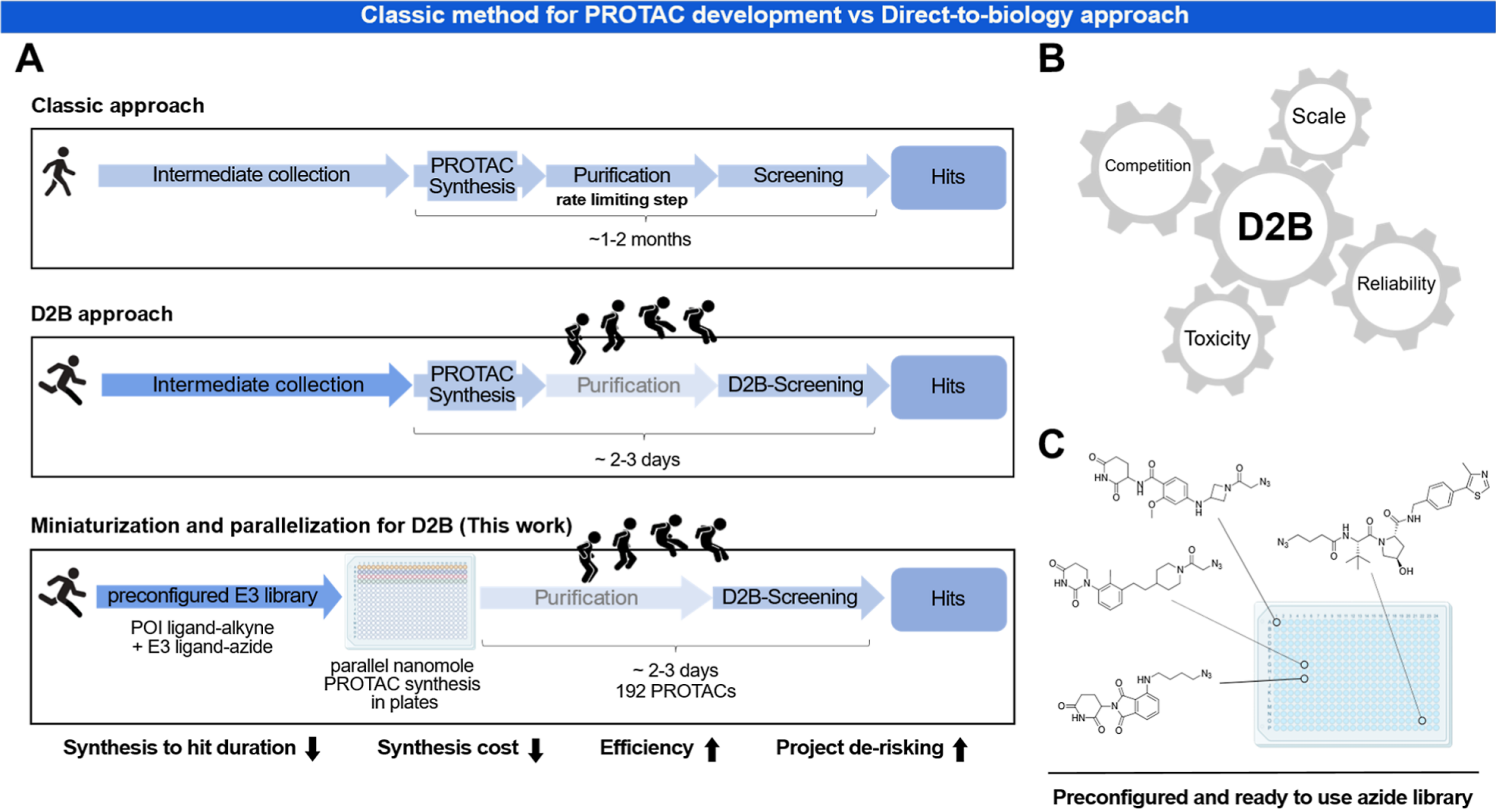
A Direct-to-biology platform for PROTAC synthesis: (A) comparison between a conventional PROTAC development and a direct-to-biology (D2B) approach. (B) Different parameters that need to be optimized for a successful D2B PROTAC development including miniaturization. (C) A chemically diverse set of azides is transferred to 384 well plates that can be directly used in the Mini-Click synthetic approach.

In a D2B approach different factors need to be optimized in order to generate reliable cellular data. In fact, unreacted starting materials and reagents can affect the cellular readout as they can act both as competitors of PROTAC POI interaction and as cytotoxic agents, which can interfere with the assay. The reaction scale can become a key factor in achieving optimal reaction conversion, often limiting the useful volume and concentration ranges for high-throughput synthesis (Figure 1B).^18^

When paired with high-throughput synthesis (HTS) a D2B approach enables quick access to a large set of degrader molecules that can be characterized using appropriate sensor cell lines that monitor cellular POI concentration. Several luminescent detection systems are suitable for developing cellular assays including luciferase complementation assays such as HiBiT or assays using fluorescent tags, for instance GFP.^19^ Given the resource intensive nature of degrader synthesis, especially with varying linker moieties, a miniaturization approach would be expected to cut down synthesis costs. Herein, we report a high-throughput, nanoscale D2B synthesis approach for the rapid assembly of PROTAC molecules in a 384 well plate format using the highly efficient and robust copper-catalyzed azide–alkyne cycloaddition (CuAAC) with subsequent cell-based evaluation (Figure 1C). This method allowed us to quickly address central TPD questions such as degradability, linkerology and the best combination of E3 ligase/POI ligand.

## Results and Discussion

### Synthesis of Alkyne-Bearing Ligands for Four Different Proteins of Interest

To study the general applicability of the developed workflow, we conducted a proof-of-concept study examining a diverse set of proteins. Target proteins from four different drug target families were selected including the well-established model system for PROTAC development Bromodomain-containing protein 4 (BRD4), soluble epoxide hydrolase (sEH), WD repeat-containing protein 5 (WDR5) and Aurora kinase A (AurkA). The targets vary in molar mass, protein family, function, structure and subcellular localization (Table S1). Since these proteins have been previously reported to be targeted by degraders, control PROTACs were available and have been used for benchmarking and as validation tools for our method.^20–23^ To enable the rapid assembly of PROTACs, validated high affinity inhibitors were combined with linkers harboring an alkyne moiety that was oriented toward the solvent region and acting as an exit vector. For each POI, four alkyne derivatives with varying linker composition with regard to length, polarity and rigidity were prepared. Alkyne (A) derivatives obtained from the highly potent BET bromodomain inhibitor JQ1 targeting BRD4 (Figure 2, top left panel) as well as AurkA inhibitor MK-5108 (Figure 2, bottom right panel) were easily accessible to yield A1-A4 (BRD4) and A13-A16 (AurkA).^20,24^ For sEH, two exit vector strategies across three different chemotypes were introduced to the alkyne set: as previously reported, ligands A5 and A6 exit at the short branch and ligands A7 and A8 at the long branch of the L-shaped binding pocket of the hydrolase domain of sEH (Figure 2, top right panel).^25^ In the case of WDR5, ligands A9-A11, based on Dimethyl-F-OICR-9429-COOH (XF056-121) and A12, derived from the previously reported WDR5 chemical probe LH168, were chosen, which target the WIN-site pocket of WDR5 and share a similar strategy for linker attachment (Figure 2, bottom left panel).^21,26^

**Figure 2:**
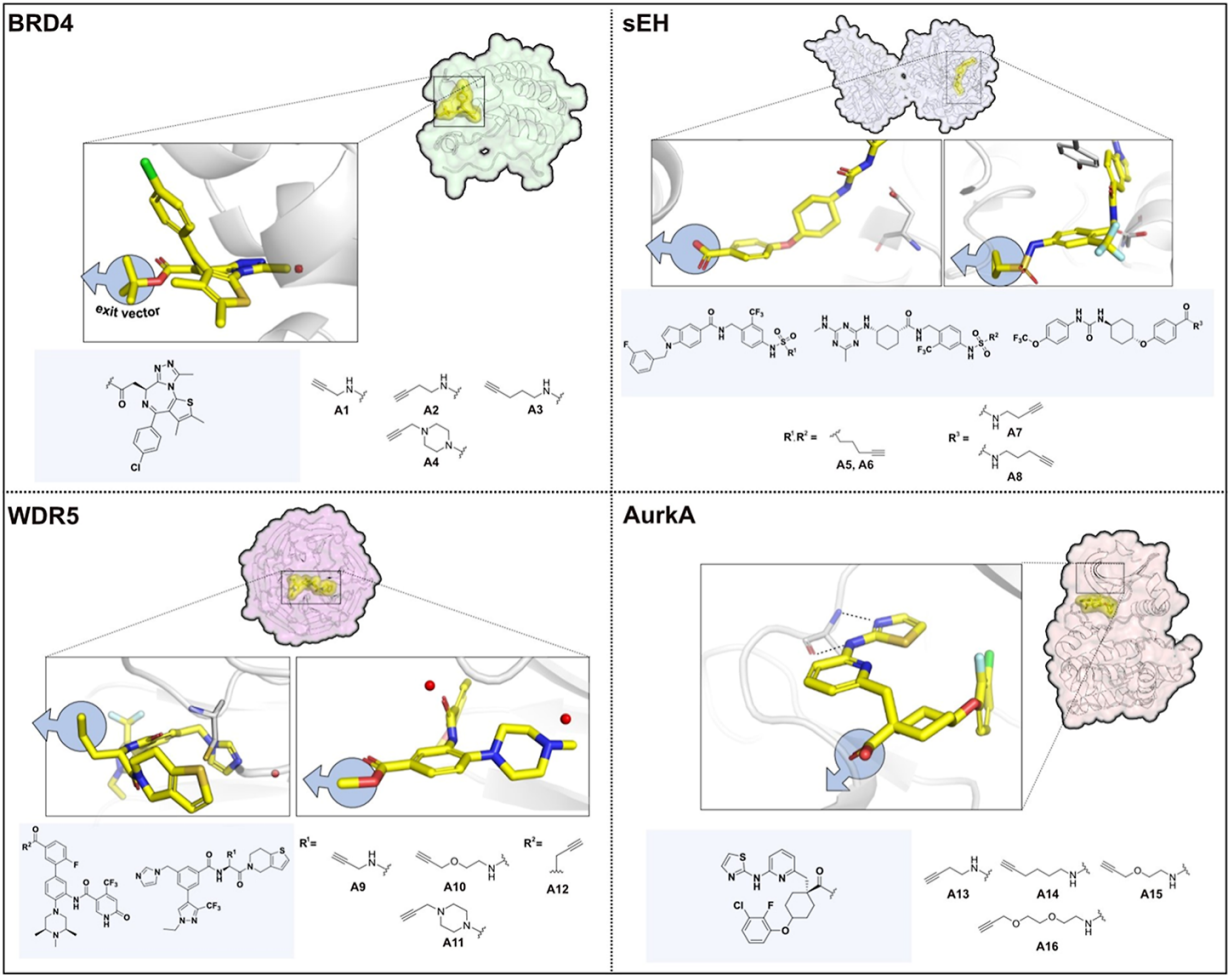
Overview of the proteins of interest used in this work: Four target proteins from different families were used in this study: Bromodomain-containing protein 4 (BRD4, top left), soluble epoxide hydrolase (sEH, top right), WD repeat-containing protein 5 (WDR5, bottom left) and Aurora kinase A (AurkA, bottom right) in complex with their corresponding ligands and exit strategies. The ligands were modified with various alkyne moieties and are shown next to the respective POI.

### Design and Synthesis of the E3 Ligand-Linker Library

Next, we synthesized a wide selection of E3 ligase ligands combined with various types and lengths of linkers bearing a terminal azide functionality, including alkyl, polyethylene glycol (PEG) and saturated heterocyclic moieties (Figure 3A–E). The E3 ligase ligand core was either commercially available or prepared on a multigram scale and further modified to yield a total of 48 azides (X1-48), with 36 analogues linked to CRBN (thalidomide, phenyl dihydrouracil^27^ and benzamide^28^) and 12 analogues linked to VHL ligands. Straight forward synthetic routes including amide couplings and S_N_2 reactions were applied to rapidly assemble the azide building blocks, yielding the final azides at 50–150 mg scale. Synthetic procedures, NMR and mass spectra for active analogues and precursors are provided in the Experimental Section and Supporting Information. With four alkyne derivatives selected per POI ligand and 48 azides in hand, a total of 192 unique PROTACs were synthetically accessible for each target protein using CuAAC reactions.

**Figure 3:**
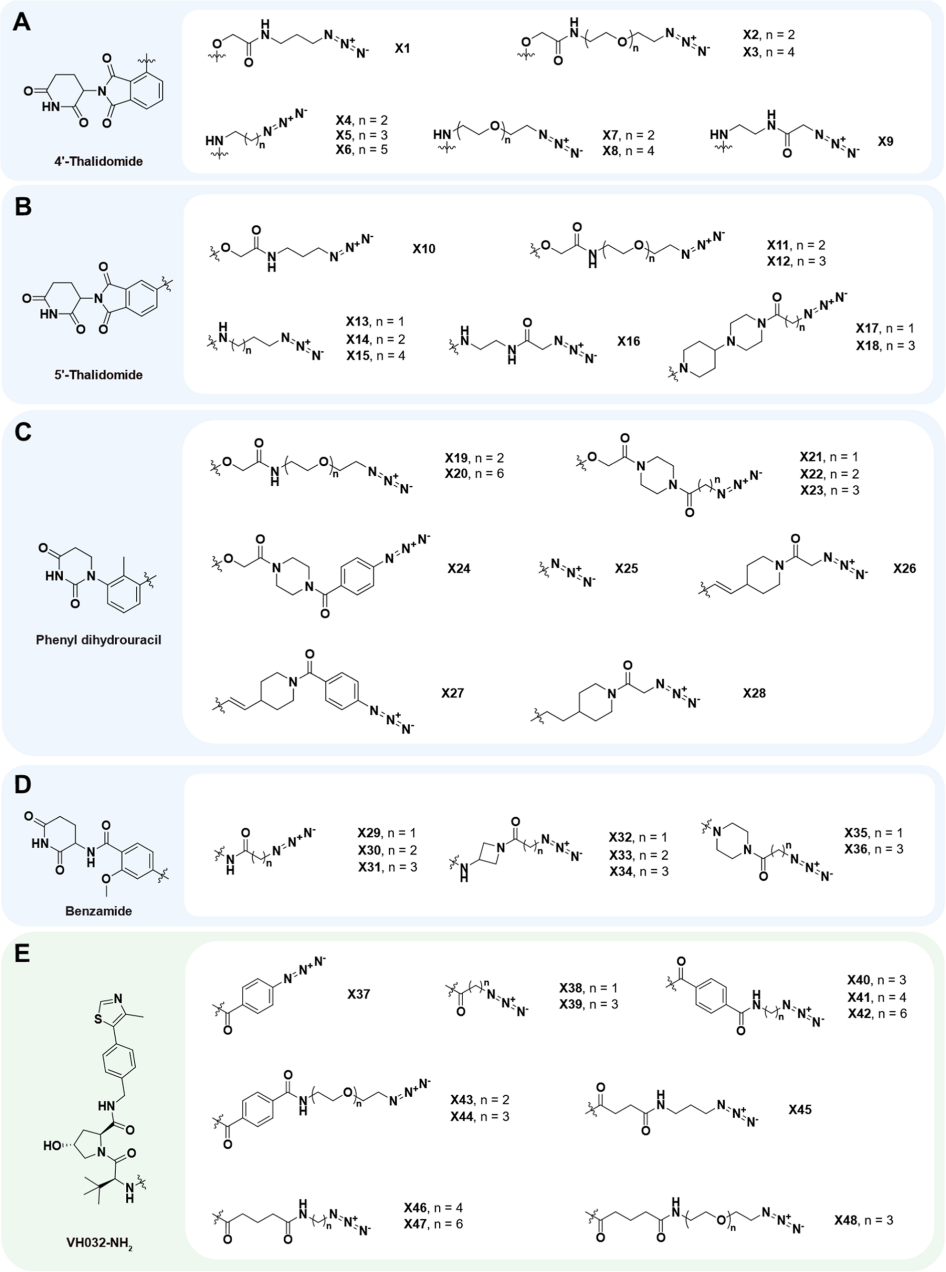
Overview of the modified E3 ligands used in this work: Chemical structures of the E3 ligase azide combinations (48 in total) targeting CRBN (blue shades) and VHL (green shade). (A) Nine derivatives based on 4′-substituted thalidomide scaffold, (B) nine derivatives based on 5′-substituted thalidomide scaffold, (C) 10 derivatives based on phenyl dihydrouracil (PDHU) scaffold, (D) eight derivatives based on benzamide scaffold and (E) 12 derivatives based on VH032-NH_2_ scaffold were synthesized.

### Optimization and Evaluation of the D2B Platform on the First Model System BRD4

To reliably apply our method, we sought to optimize the reaction conditions for the CuAAC by using BRD4 as a target protein. Since the biological evaluation of the PROTACs was performed in a D2B approach, the use of Cu^I^ as a catalyst and its associated effects on cell viability needed to be assessed.^29^ For this purpose, we examined different reaction conditions, modifying reactant concentration and volume, with the intent to lower the used Cu^I^ concentration (Figure 4B,C). The optimal and most reproducible reaction conditions were identified with reactant concentrations of 20 mM and a reaction volume of 5 μL. These conditions offered the best balance, providing the lowest possible scale, consistently resulting in high conversion rates, and the low Cu^I^ concentration had negligible influence on cell viability in the HiBiT assay (Figures 4E and S5, threshold concentration of our copper system above 100 μM). In addition, the specified reaction conditions enable longer use of the library, as on average only ∼50 μg of a single azide was utilized per reaction. Finally, we set the equivalent of alkyne and azide at 1 eq., the equivalent of CuSO_4_·5H_2_O and sodium ascorbate at 0.3 eq. and the reaction temperature at rt (Figure 4A). A total of 192 unique BRD4 PROTACs were synthesized after 24 h reaction time and the conversion rate of each well was determined by HPLC/UV analysis. Among the 192 reactions, 106 reached reaction conversions of more than 95% and an average conversion rate of 92% was achieved. This indicated the high reliability and broad scope of our method (Figure 4D). We therefore proceeded to perform HiBiT lytic assay-based screening using the crude BRD4 PROTACs at 1 μM concentration. In parallel, a cell-viability assay was conducted to rule out false-positive screening results caused by cytotoxicity. No significant cell toxicity was observed at a final crude concentration of 2.5 μM after 24 h (Figure S6).

**Figure 4:**
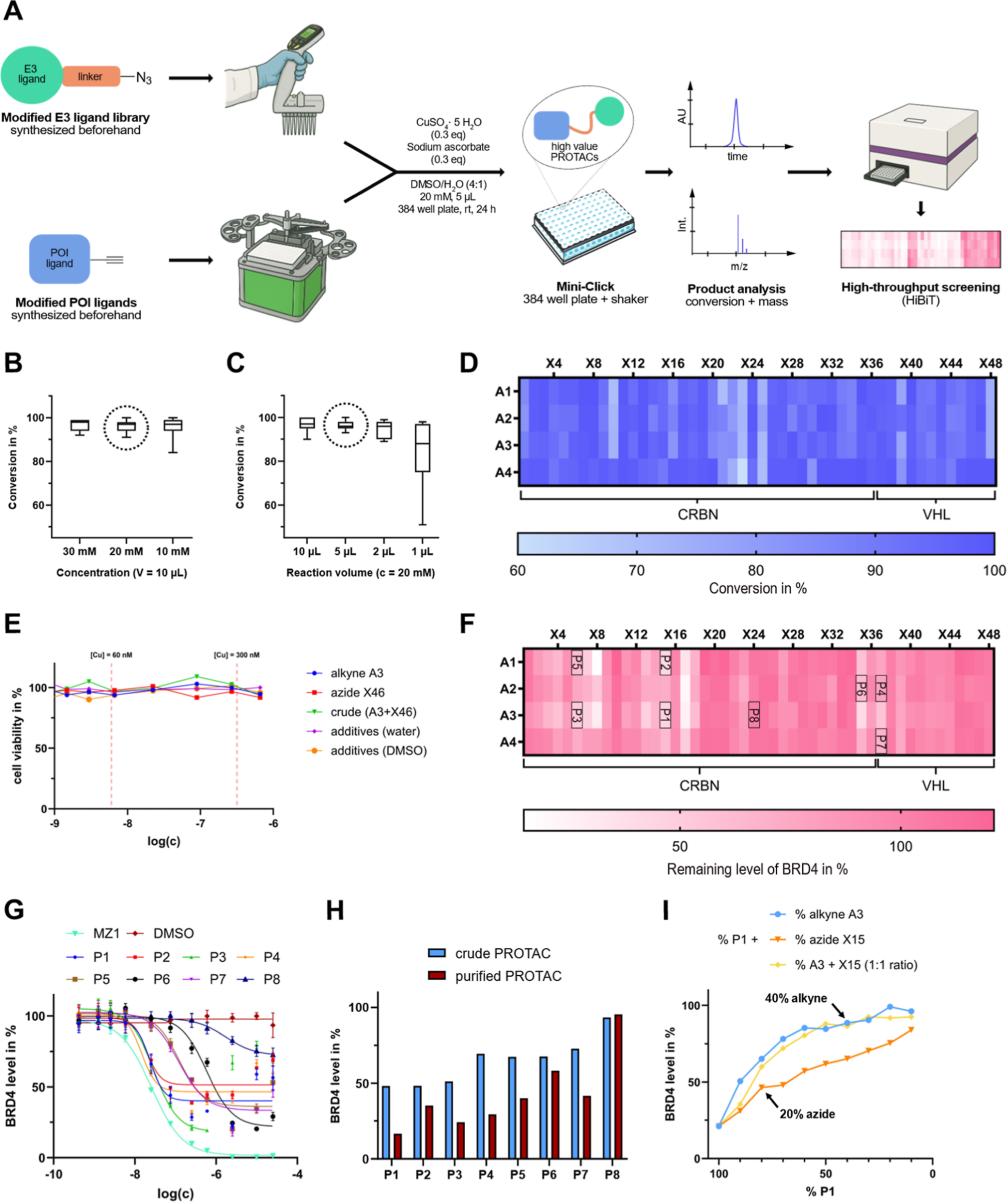
Assembly of the PROTAC library using CuAAC and identification of degraders from D2B screening: (A) general workflow of the miniaturized PROTAC synthesis in 384 plates utilizing CuAAC described in this work: Library development including POI/E3-ligands derivatized with alkynes or azides, respectively. Automated and semiautomated dispensing for reaction set up. Product formation monitoring via HPLC-MS and hit identification using direct cell-based screening. (B) Fluctuation of the overall conversion rate of the BRD4-targeting ligands with varying reactant concentrations. (C) Effects of reaction volumes on conversion rates. (D) Conversion rates of the CuAAC chemistry for BRD4 PROTACs, indicated by a color-coded heat-map based on HPLC/UV data. (E) Cell viability data after 24 h with components used in an exemplary click reaction (CellTiter-Glo). (F) HiBiT lytic assay-based screening results for BRD4 at 1 μM after 6 h PROTAC exposure. (G) Dose response curves for the purified PROTACs P1-8 using the HiBiT detection system. MZ1 was used as a positive control.^30^ (H) HiBiT lytic assay data for purified BRD4 PROTACs P1-8 compared to the corresponding crude reaction mixtures after 6 h treatment, using a theoretical 200 nM PROTAC concentration assuming 100% conversion rates. (I) Spiking experiment for PROTAC P1 with alkyne A3 (blue), azide X15 (orange) and a mixture (1:1) of A3 and X15 (yellow) at 200 nM PROTAC concentration after 6 h exposure.

Examination of our HiBiT lytic assay data revealed that after 6 h, several PROTACs significantly decreased BRD4 levels, with 11 PROTACs achieving degradation above 50% (Figure 4F). Noticeably, among the different CRBN targeting chemotypes only the thalidomide based (X1–X18) PROTACs showed a significant degradation efficacy, indicating that the right choice of E3 ligase ligand scaffold has a crucial effect on the performance of the PROTAC. Next, eight PROTACs P1-8, ranging from high to low degradation efficacy in our D2B screen, were resynthesized at larger scale and purified to validate the hits generated with the developed plate based synthetic method (P1–P8, Data S1). Analysis of the resynthesized PROTACs revealed degradation of BRD4 in cells in a dose dependent manner. Importantly, the determined PROTAC potencies of the purified degraders correlated well with degradation levels of the corresponding molecules synthesized and evaluated by our D2B approach (Figure 4G). Additionally, a HiBiT lytic assay comparing the purified and crude PROTACs P1-8, was carried out at 200 nM (Figure 4H). We observed a significant difference in degradation efficacy when comparing purified with crude PROTACs (∼25% difference). These data indicated that competition of PROTAC binding to the POI or E3 ligases with unreacted reactants in crude reaction mixtures, reduced sensitivity detecting degrader molecules. To confirm that the lower degradation efficacy was due to unreacted POI and E3 ligands, we designed “spiking experiments”, in which a pure PROTAC (P1) was added to a solution of the corresponding reaction components (alkyne A3, azide X15 and a mixture of A3 and X15 in 1:1 ratio), mimicking partial conversions of the CuAAC reaction (Figure 4I). The obtained degradation data revealed that the presence of just 10% unreacted alkyne decreased the degradation efficacy by half compared to pure PROTAC P1 (24 vs 51% residual BRD4 levels). The effect of 10% residual azide was smaller but still noteworthy (24 vs 30% residual BRD4 levels), probably due to weaker E3 ligand binding affinities. To verify that the observed degradation was dependent on the proteasomal pathway, cells were treated with P1 together with either the proteasome inhibitor MG132 or the neddylation inhibitor MLN4924 (Figures S8 and S9). Under these coincubation conditions, no BRD4 degradation was observed. Additionally, the negative control compound for P1 (methylated at the imide nitrogen) failed to induce degradation, confirming that BRD4 degradation was E3 ligase dependent. Based on the excellent conversion rates we obtained, the developed screening method was sensitive enough to identify 11 active BRD4 PROTACs with a degradation efficacy above 50%, resulting in an overall hit rate of approximately 6%. To prioritize the resynthesized hits, we evaluated solubility limits in PBS, metabolic stability, and cytotoxicity of these PROTACs (see Supporting Information Figures S7 and S10–S12). The studied PROTACs showed similar performance in terms of solubility and cytotoxicity. Regarding their metabolic stability, PROTACs with linear PEG or alkyl linkers showed poorer performance than nitrogen containing rigid ring systems, and thalidomide-based ligands were less stable compared to benzamide, phenyl dihydrouracil or VHL-ligand based PROTACs.

After successfully implementing the D2B platform with the model protein BRD4, we used the established protocol to identify active PROTACs for three additional structurally diverse proteins: sEH, WDR5 and AurkA. The goal of this project was 2-fold: first, to demonstrate the general applicability of our D2B platform; and second, to address key questions in PROTAC optimization, such as the roles of incubation times, linker attachment points and screening concentrations, as well as E3 ligase compatibility of a selected target.

Targeting sEH, our CuAAC generated PROTACs were tested in a HiBiT lytic assay system established in HeLa cells at two different incubation times of 6 and 18 h, respectively. A projected PROTAC concentration of 200 nM was used. Applying a degradation threshold of 45% for sEH, we identified 10 highly active PROTACs and yielded an overall hit rate of 5%. Notably, the azide X8 exhibited degradation independent of the chemotype of the sEH ligand used (Figure 5A, left side, highlighted in blue). In general, most potent sEH degradation was observed with alkyne A5 and CRBN-based azides. Within the same linker chemotype, longer linker moieties resulted in improved degradation potencies. For other alkynes (A6–A8), this trend was less consistent. Initially, no hits were detected with VHL-based azides after 6 h. However, at the 18 h time point, the combination of PROTAC A6 + X42 led to a 56% decrease of sEH levels (Figure 5A,B). Interestingly, VHL-based hits were exclusively observed using the POI ligand A6, underscoring the importance of utilizing diverse POI ligands when available (Figure 5A, right side, highlighted in blue). To date, no effective VHL-based PROTACs have been reported for sEH, and X42 may serve as a promising starting point for further development of VHL-recruiting sEH degraders. These results also highlight the value of screening across multiple time points to gain a more comprehensive understanding of degradation kinetics.

**Figure 5:**
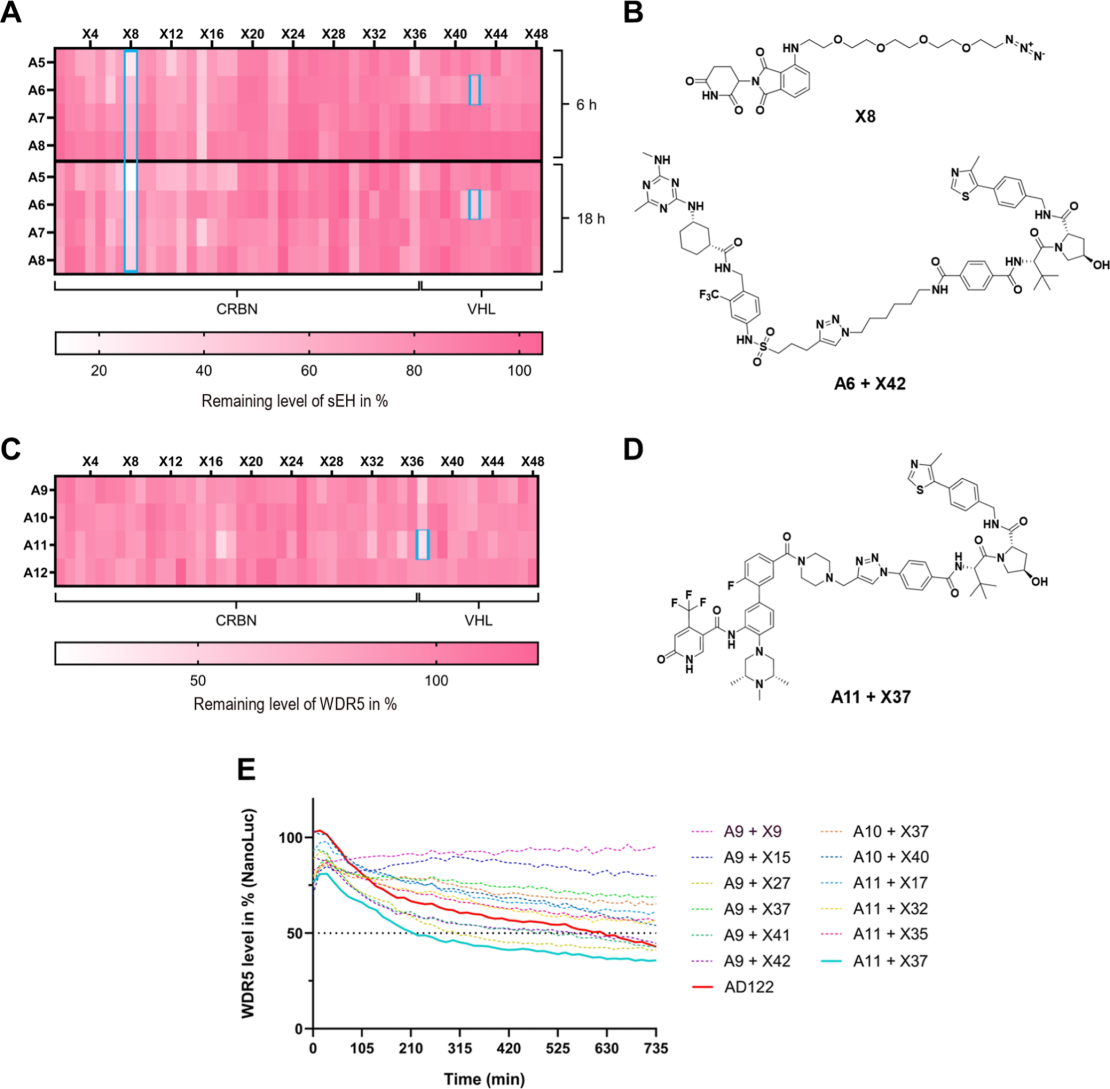
Identification of active PROTACs for sEH and WDR5: (A) HiBiT lytic assay-based screening data for sEH at a PROTAC concentration of 200 nM after 6- and 18 h incubation time. The best performing azide (X8) and the time dependent degradation effect of VHL containing PROTAC A6 + X42 are highlighted (blue). (B) Chemical structures of the azide X8 and PROTAC A6 + X42. (C) HiBiT lytic assay-based screening data for WDR5 at a PROTAC concentration of 1 μM after an incubation time of 6 h. (D) Chemical structure of the PROTAC A11 + X37 that induced the most efficient degradation of WDR5. (E) NanoLuc time dependent live cell measurement monitoring the degradation of WDR5 at a PROTAC concentration of 1 μM (crude reaction mixtures and AD122).

Next, we explored our D2B platform for a screen identifying degraders of the nuclear protein WDR5 for which we developed PROTACs previously and also established a HiBiT assay technology.^7,21^ In our screen for WDR5-targeting PROTACs, three PROTACs were identified that degraded WDR5 by more than 45% (Figure 5C). In line with previous results, we observed that degradation of WDR5 can be achieved with both CRBN- and VHL-based PROTACs.^21,31^ Among them, A11 + X37 emerged as the most potent hit (Figure 5D). Interestingly, the second ligand used in this study based on a recently developed ligand LH168 ^26^ yielded no hits, despite the overall excellent conversion rates observed for this ligand (Figure S2). This may be attributed to a different linker attachment point or the observed slow binding kinetics of this compound, potentially reducing the effectiveness of this ligand for PROTAC design. In an orthogonal live cell, NanoLuc luminescence assay in MV4-11 cells, A11 + X37 was confirmed as the most potent hit (Figure 5E), surpassing the reference PROTAC AD122 ^7,21^ during the 12 h incubation time. In general, more hits were identified using this screening method, possibly due to a higher sensitivity of this assay system.

The last case study focused on the mitotic kinase AurkA. Also, for this POI, we previously identified PROTACs using traditional PROTAC development strategies.^20,24^ In addition, AurkA has been identified as a highly “degradable” kinase using nonselective kinase inhibitors making it an excellent model system for validation of our platform.^32^ Indeed, when screening the generated D2B PROTAC set, we observed excellent hit rates (64% of the crude PROTACs show a degradation above 60% at 1 μM and 18% at 200 nM crude PROTAC concentration, respectively). Due to the large number of potent hits, we rescreened the library in HiBiT lytic assays (MV4-11 cells) at a reduced PROTAC concentration (50 nM) to better differentiate moderate from potent degraders (Figure 6A). Applying a 60% degradation threshold at this concentration, we still identified 8 potent hits at this low screening concentration (hit rate of 4%). We ranked the PROTACs according to their degradation potency at 50 nM and correlated the HiBiT degradation efficacy values with data measured at 1 μM (Figure 6B). The comparison of these data showed excellent overall correlation at both concentrations allowing us to identify most active PROTACs (A16 + X7 and A13 + X30) for this highly degradable POI (Figure 6C). The high hit rate allowed us to derive structure–activity relationship trends (Figure 6D, at 50 nM): the PEG linkers with an *N*-functionalization at the thalidomide (Thal) resulted in potent POI degradation, followed by alkyl linkers. The use of benzamide (BA)-derived ligands in combination with an alkyl linker was as effective as the combination of thalidomide and PEG linkers. Overall, ether-functionalized (C–O) phenyl dihydrouracil (PDHU) ligands appeared to be less favorable compared to C­(sp^2^)–C­(sp^3^) exit vectors. BA-derived CRBN ligands demonstrated comparable performance to those derived from thalidomide but it is likely that these PROTACs show a more favorable profile in terms of neo-substrate degradation and chemical stability.^28^

**Figure 6:**
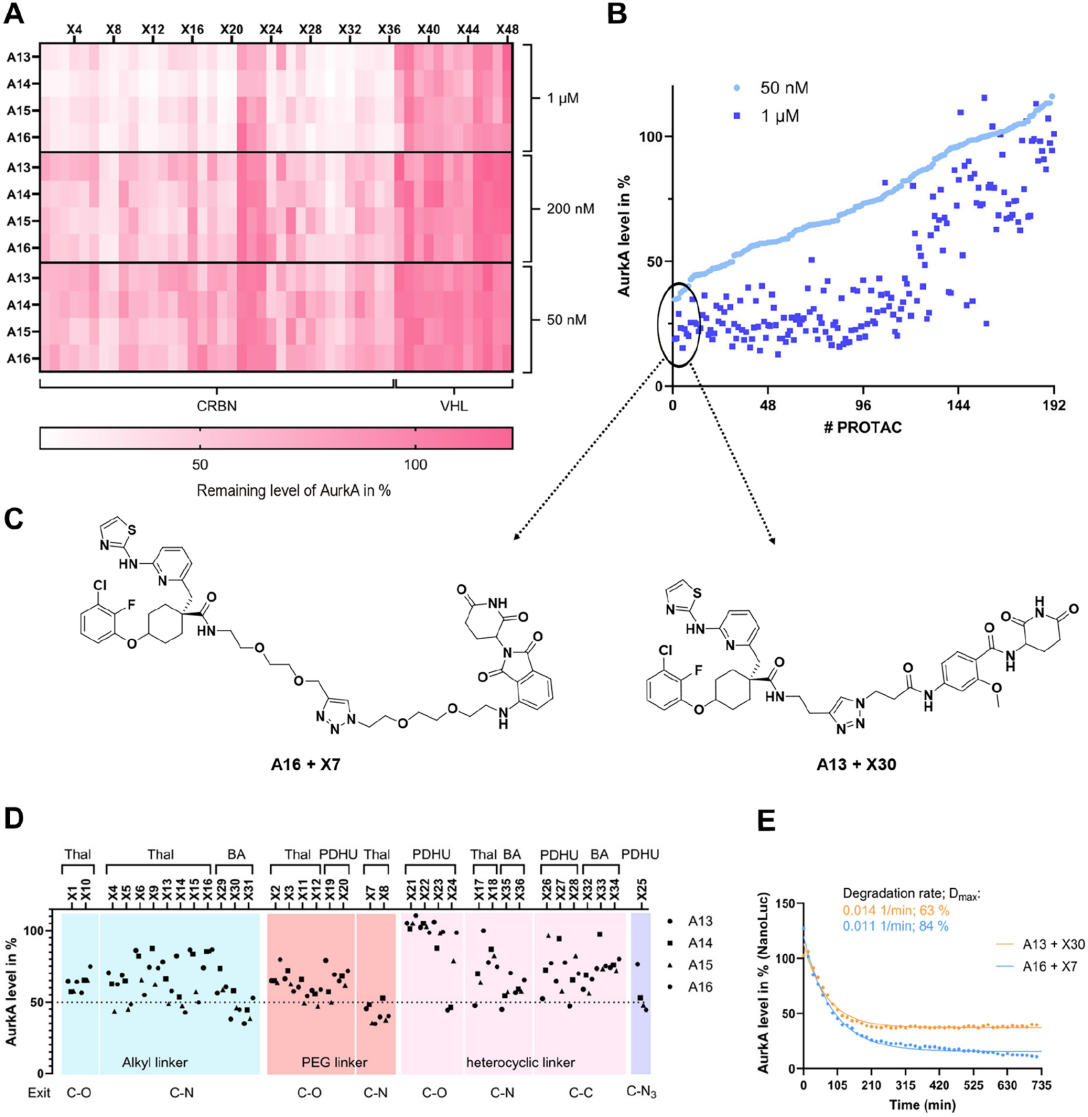
Identification of active PROTACs for AurkA: (A) HiBiT lytic assay-based screening data for AurkA at 50 nM, 200 nM and 1 μM PROTAC concentration after 6 h incubation time. The clusters of CRBN and VHL containing PROTACs are labeled beneath the heatmap. (B) Screening hits sorted according to degradation efficacy using HiBiT lytic assay data measured at a PROTAC concentration of 50 nM. The data showed good reproducibility of screens carried out at 1 μM and 50 nM PROTAC concentration and identified eight PROTACs with more than 60% POI degradation at 50 nM. (C) Chemical structures of the PROTACs A16 + X7 and A13 + X30 that induce efficient degradation of AurkA (Dmax >60%) at a PROTAC concentration of 50 nM. (D) HiBiT lytic assay-based screening data for AurkA of crude reaction mixtures of CRBN ligand-bearing PROTACs at a PROTAC concentration of 50 nM after 6 h treatment. The data is sorted by different components of the PROTACs: E3 ligand (Thalidomide: Thal, Benzamide: BA, PDHU: phenyl dihydrouracil), linker (alkyl, PEG, heterocyclic) and exit vector composition (C–O, C–N, C–C or C–N_3_). (E) NanoLuc time dependent live cell measurement monitoring the degradation of AurkA at a PROTAC concentration of 1 μM (crude reaction mixtures of PROTACs A16 + X7 and A13 + X30). The initial phase of each concentration-dependent degradation curve was fitted using a one-component exponential decay model in GraphPad Prism. From this analysis, the best fit parameters *K* (degradation rate) and plateau (minimum remaining fraction) were determined.

Overall, heterocyclic linker systems were less effective, indicating that it is necessary to test multiple and diverse heterocycles, as these linkers often demonstrate better metabolic stability^33^ and might enhance pharmacokinetic properties.^34^ Our parallel synthetic approach is well suited for this purpose, as a rapid evaluation of the unpurified PROTACs can be used to quickly investigate an alternative linker choice that might be better suited for an in vivo application. Metabolic stability studies of the resynthesized PROTACs showed that the introduction of a piperazine group provided enhanced stability that was independent of the selected E3 ligand (P5 and P7, Figure S12).

A detailed analysis of degradation kinetics using a NanoLuc live cell system allowed us to gain insight into time dependent effects (Figures 6E and S4). We investigated both the degradation rate and the *D*_max_ within the defined time window. PROTACs A13 + X30 and A16 + X7 both showed a rapid onset of degradation with a degradation rate of 0.014 min^–1^ and 0.011 min^–1^ ,respectively. Interestingly, A13 + X30 reached a degradation plateau at *D*_max_ of 63% while A16 + X7 even outperformed the other hits with a *D*_max_ of 84% (see also Figure S3).

## Conclusion and Outlook

The empirical nature of TPD drug discovery poses a significant hurdle toward the development of new degrader molecules. We used a D2B approach that allowed us to rapidly interrogate linker SAR, estimate trends in hijackable E3 ligases for degradation and quickly evaluate adequate POI ligand choices. In contrast to classical medicinal chemistry programs, with systematic synthesis and isolation of chemical matter, we were able to address all variables of PROTAC optimization in parallel without the need for time-consuming PROTAC purification. This approach positively impacted the overall synthetic output and substantially reduced the time needed from molecule assembly to cellular assay. While it is true that significant synthetic work is needed beforehand for the synthesis of the library azide components and the design of corresponding alkynes, we believe that the overall value of this technology greatly outweighs the ab initio effort, especially since the same azide collection can be applied to multiple targets. In fact, sustainability is not only achieved via repurposing of the library, we managed to drastically decrease the scale of the CuAAC reaction with a consumption of a single azide in the two-digit μg range. D2B combined with high-throughput screening methods enables the generation of large SAR data sets. Analysis and handling of this data dense output can become a bottleneck and should be combined with computational chemistry and machine learning systems to further streamline PROTAC design and enhance our understanding for adequate POI/E3 combinations, linker choice and overall degradability of a target. The D2B nanoscale synthesis reported in this study has been used to degrade proteins from four different families and we anticipate this methodology to be applied to disease relevant targets in future studies. We have identified several hits for each protein that can be further developed in future optimization efforts. By applying established best-practice experience, the most promising hit can be prioritized. Ideally, it will feature a more rigid linker and a E3 ligase ligand with favorable chemical and pharmacological stability. Cell viability assays should be run in parallel to assess toxicity of the PROTAC and dependent on the target genetic approaches should be used to distinguish between any observed off-target toxicity from inhibition-based toxicity. We offer this method as an enabling technology to the TPD community interested in quick and efficient assessment of the degradability of a target of interest. This is further facilitated by introducing a ready-to-use, preplated storage format of the library that allows rapid assembly of degrader molecules. Future efforts will be focused on expanding the library with the aim of introducing more complex and rigid linker systems that have been reported to positively influence physicochemical and pharmacokinetic properties as well as new E3 ligands that may also allow tissue specific degradation based on E3 ligase expression and will increase the diversity of the E3-ligand/linker library.

## Experimental Section

### Synthetic Procedures and Characterization

The synthesis of compounds will be explained in the following. All chemicals were purchased from common suppliers with a purity ≥95% and were used without further purification. The solvents with an analytical grade were obtained from VWR Chemicals and Merck. Dried solvents were purchased from Acros, stored over molecular sieves and kept under an inert atmosphere. Solvents used in column chromatography or flash column chromatography were technical grade. Perdeuterated solvents were purchased from Eurofins. All microwave-assisted reactions were carried out in sealed reaction vials (0.5–10 mL) with a Biotage Initiator Microwave System with Robot Eight by Biotage. Flash column chromatography purifications were carried out with a puriFlash XS 520 Plus system from Interchim. For normal-phase chromatography PF-30SIHP-JP-F0024 columns were used with a gradient of DCM and methanol serving as the mobile phase. For reverse-phase chromatography PF-30C18HP-F0012 and PF-30C18HP-F0025 columns were used with a gradient of H_2_O and ACN serving as the mobile phase. To monitor the progression of the reactions and determining the purity of compounds analytical high-performance liquid chromatography (HPLC) was performed. A 1260 Infinity II LC System consisting of the multisampler G7167A, the column compartment G7116A, the multicolumn thermostat G7116A, the flexible pump G7104C, a single quadrupole LC/MSD system InfinityLab G6125B and the diode array detector HS G7117C by the company Agilent Technologies was used for this purpose. An ACE UltraCore Super C18 column (150 × 3.0 mm) from Avantor was used as the stationary phase and a gradient of H_2_O and ACN with 0.1% formic acid served as the mobile phase. UV detection took place at wavelengths of 254 and 280 nm. The following gradient was used: 0 min: 5% B—2 min: 80% B—5 min: 95% B—7 min: 95% B (flow rate of 0.6 mL/min). All compounds resynthesized for the BRD4 case study and all azides and alkynes have a purity of >95%, determined by HPLC. All other PROTACs tested are used as crude samples. Nuclear magnetic resonance (NMR) spectra were recorded with spectrometers DPX250 (250 MHz ^1^H), AV300 (300 MHz ^1^H, 282 MHz ^19^F), AV400 (400 MHz ^1^H, 101 MHz ^13^C, 377 MHz ^19^F) and AV500 (500 MHz ^1^H, 126 MHz ^13^C) from Bruker with all the measurements being performed at rt and in deuterated solvents. Chemical shifts (δ) are reported in parts per million (ppm) and refer to the internal standard tetramethylsilane at 0.00 ppm and to the residual solvent signal. DMSO-*d*_6_, acetone-*d*_6_, methylene chloride-*d*_2_ and chloroform-*d* were used as a solvent, and the spectra were calibrated to the solvent signal: 2.50 ppm (^1^H NMR) or 39.52 ppm (^13^C NMR) for DMSO-*d*_6_, 2.05 ppm (^1^H NMR) or 206.3 ppm (^13^C NMR) for acetone-*d*_6_, 5.32 ppm (^1^H NMR) or 53.8 ppm (^13^C NMR) for methylene chloride-*d*_2_ and 7.26 ppm (^1^H NMR) or 77.2 ppm (^13^C NMR) for chloroform-*d*. The coupling constant *J* was stated in Hz. The multiplicity M of the signals in the spectra was characterized by following abbreviations: s (singlet), d (doublet), dd (doublet of doublets), t (triplet), td (triplet of doublets), quartet (q), quintet (quin), septet (sept), m (multiplet).

### Safety Considerations

We have not encountered any explosion during the handling of azide species in all procedures listed in this document. However, for safety concerns, sodium azide (NaN_3_) and all organic azide compounds should be treated as explosive and toxic substances. When handling sodium azide or organic azide compounds, care should be taken to avoid strong mechanical shock or friction, and contact with metal apparatus (syringe needle, metal spatula, etc.) should be avoided. Instead, we recommend using a plastic spatula or plastic pipet.

#### Setup of the Miniaturized CuAAC Reactions in 384 Well Plate Format

Using the example of the synthesis of 192 unique BRD4 PROTACs: 50 mM stock solutions of alkynes **A1-4** and azides **X1-48** in DMSO as well as 60 mM stock solutions of CuSO_4_·5H_2_O and sodium ascorbate in H_2_O were prepared. First, 4 × 2 μL of each azide stock solution were manually pipetted with an E1-ClipTip Electronic Multichannel pipet on to the 384 well plate (4 × 2 μL x 48, in total 192 occupied wells with 2 μL of the respective azide stock solution). Next, 2 μL of the respective alkyne (48 wells per alkyne), 0.5 μL of CuSO_4_·5H_2_O (in each well) and 0.5 μL of sodium ascorbate (in each well) stock solution were added to the 384 well plate with the MANTIS Liquid Dispenser. The plate was sealed and put on a plate shaker (300 rpm) at rt for 24 h (In-depth description of miniaturized plate setup in Supporting Information, Figure S1).

#### General Procedure A for Amide Coupling

Carboxylic acid (1.0 equiv) and HATU (1.2–1.3 equiv) were dissolved in DMF. Amine (1.0 equiv) and DIPEA (3.0 equiv) were added to the resulting mixture and it was stirred at rt for 2 h. The solvent was removed under reduced pressure and the crude product was purified by flash chromatography using acetonitrile/water as an eluent.

#### General Procedure B for Amide Coupling

Carboxylic acid (1.0 equiv), 1-methyl-1*H*-imidazole (3.5 equiv), amine (1.0–1.5 equiv) and *N*,*N*,*N*′,*N*′-tetramethylchloroformamidinium hexafluorophosphate (TCFH) (1.2 equiv) were dissolved in dry acetonitrile. The reaction mixture was stirred at rt for 18 h. The reaction mixture was diluted with EtOAc and washed with saturated NaHCO_3_ (3×) and brine (1×). The organic phase was dried over MgSO_4_ and the solvent was removed under reduced pressure. The crude product was purified by flash chromatography using acetonitrile/water as an eluent.

#### General Procedure C for Microwave-Assisted Nucleophilic Aromatic Substitution

Fluoro derivative (1.0 equiv), amine (1.2–1.5 equiv) and DIPEA (3.0–4.0 equiv) were charged in a microwave vial and dissolved in DMSO. The suspension was degassed with argon under sonication. The vial was heated in a microwave oven to 150 °C for 5 min. The solvent was removed under reduced pressure and the crude product was purified by flash chromatography using acetonitrile/water as an eluent.

#### General Procedure D for *N*-Boc Deprotection

*N*-Boc protected amines were dissolved in dry DCM. The flask was cooled to 0 °C in an ice bath and trifluoroacetic acid (TFA) in an excess was added dropwise. The solution was stirred at rt for 2 h. The solvent was removed under reduced pressure and the crude product used without further purification.

#### General Procedure E for Copper-Catalyzed Azide–Alkyne Cycloaddition

Azide (1.0 equiv) and Alkyne (1.0 equiv) were dissolved in DMSO. Copper sulfate pentahydrate (0.3 equiv) and sodium ascorbate (0.3 equiv) were dissolved in water and added to the reaction mixture. The solution was stirred at rt for 18 h. The solvent was removed under reduced pressure and the crude product was purified by flash chromatography using acetonitrile/water as an eluent.

##### Synthesis of (*S*)-2-(4-(4-Chlorophenyl)-2,3,9-trimethyl-6*H*-thieno­[3,2-*f*]­[1,2,4]­triazolo­[4,3-*a*]­[1,4]­diazepin-6-yl)-*N*-(prop-2-yn-1-yl)­acetamide (**A1**)

The title compound was prepared according to general procedure A, using (*S*)-2-(4-(4-chlorophenyl)-2,3,9-trimethyl-6*H*-thieno­[3,2-*f*]­[1,2,4]­triazolo­[4,3-*a*]­[1,4]­diazepin-6-yl)­acetic acid (150 mg, 0.374 mmol), prop-2-yn-1-amine (20 mg, 0.374 mmol.), HATU (184 mg, 0.486 mmol) and DIPEA (195 μL, 1.12 mmol). The title compound was obtained as a colorless solid (152 mg, 93%). ^1^H NMR (400 MHz, DMSO-*d*_6_) δ: 8.67 (t, *J* = 5.6 Hz, 1H), 7.46 (dd, 4H), 4.50 (dd, *J* = 8.6, 5.7 Hz), 4.03–3.95 (m1H), 3.91–3.82 (m, 1H), 3.21–3.14 (m, 2H), 2.60 (s, 3H), 2.41 (s, 3H), 1.62 (s, 3H). ^13^C NMR (101 MHz, DMSO-*d*_6_) δ: 169.7, 163.0, 155.1, 149.8, 136.8, 135.2, 132.3, 130.7, 130.2, 129.8, 129.6, 128.5, 82.4, 72.1, 53.8, 37.9, 37.6, 18.8, 14.1, 12.7, 11.3. ESI-MS: *m*/*z* = 438.10 ([M + H]^+^).

##### Synthesis of (*S*)-*N*-(But-3-yn-1-yl)-2-(4-(4-chlorophenyl)-2,3,9-trimethyl-6*H*-thieno­[3,2-*f*]­[1,2,4]­triazolo­[4,3-*a*]­[1,4]­diazepin-6-yl)­acetamide (**A2**)

The title compound was prepared according to general procedure A, using (*S*)-2-(4-(4-chlorophenyl)-2,3,9-trimethyl-6*H*-thieno­[3,2-*f*]­[1,2,4]­triazolo­[4,3-*a*]­[1,4]­diazepin-6-yl)­acetic acid (100 mg, 0.249 mmol), but-3-yn-1-amine (26 mg, 0.25 mmol.), HATU (123 mg, 0.324 mmol) and DIPEA (130 μL, 0.748 mmol). The title compound was obtained as a colorless solid (106 mg, 95%). ^1^H NMR (400 MHz, DMSO-*d*_6_) δ: 8.41 (t, *J* = 5.7 Hz), 7.46 (dd, 4H), 4.51 (dd, *J* = 8.4, 5.7 Hz, 1H), 3.33–3.13 (m, 4H), 2.86 (t, *J* = 2.7 Hz, 1H), 2.59 (s, 3H), 2.54 (s, 1H), 2.50 (s, 1H), 2.41 (s, 3H), 2.37–2.32 (m, 2H), 1.62 (s, 3H). ^13^C NMR (101 MHz, DMSO-*d*_6_) δ: 169.7, 163.0, 155.1, 149.8, 136.75, 135.2, 132.3, 130.7, 130.2, 129.8, 129.6, 128.5, 82.4, 72.1, 53.8, 37.9, 37.6, 18.8, 14.1, 12.7, 11.3. ESI-MS: *m*/*z* = 452.10 ([M + H]^+^).

##### Synthesis of (*S*)-2-(4-(4-Chlorophenyl)-2,3,9-trimethyl-6*H*-thieno­[3,2-*f*]­[1,2,4]­triazolo­[4,3-*a*]­[1,4]­diazepin-6-yl)-*N*-(pent-4-yn-1-yl)­acetamide (**A3**)

The title compound was prepared according to general procedure A, using (*S*)-2-(4-(4-chlorophenyl)-2,3,9-trimethyl-6*H*-thieno­[3,2-*f*]­[1,2,4]­triazolo­[4,3-*a*]­[1,4]­diazepin-6-yl)­acetic acid (100 mg, 0.249 mmol), pent-4-yn-1-amine (30 mg, 0.25 mmol.), HATU (123 mg, 0.324 mmol) and DIPEA (130 μL, 0.748 mmol). The title compound was obtained as a colorless solid (112 mg, 97%). ^1^H NMR (400 MHz, DMSO-*d*_6_) δ: 8.23 (t, *J* = 5.6 Hz, 1H), 7.46 (dd, 4H), 4.51 (dd, *J* = 8.2, 6.0 Hz, 1H), 3.26–3.08 (m, 3H), 2.80 (t, *J* = 2.7 Hz, 1H), 2.59 (s, 3H), 2.41 (s, 3H), 2.21 (td, *J* = 6.9, 3.9 Hz, 2H), 1.63 (d, *J* = 4.9 Hz, 5H). ^13^C NMR (101 MHz, DMSO-*d*_6_) δ: 169.6, 163.1, 155.1, 149.8, 136.8, 135.2, 132.3, 130.7, 130.1, 129.8, 129.6, 128.5, 84.1, 71.3, 53.9, 37.6, 28.3, 15.4, 14.0, 12.7, 11.3. ESI-MS: *m*/*z* = 466.15 ([M + H]^+^).

##### Synthesis of (*S*)-2-(4-(4-Chlorophenyl)-2,3,9-trimethyl-6*H*-thieno­[3,2-*f*]­[1,2,4]­triazolo­[4,3-*a*]­[1,4]­diazepin-6-yl)-1-(4-(prop-2-yn-1-yl)­piperazin-1-yl)­ethan-1-one (**A4**)

The title compound was prepared according to general procedure A, using (*S*)-2-(4-(4-chlorophenyl)-2,3,9-trimethyl-6*H*-thieno­[3,2-*f*]­[1,2,4]­triazolo­[4,3-*a*]­[1,4]­diazepin-6-yl)­acetic acid (100 mg, 0.249 mmol), 1-(prop-2-yn-1-yl)­piperazine (31 mg, 0.25 mmol.), HATU (123 mg, 0.324 mmol) and DIPEA (130 μL, 0.748 mmol). The title compound was obtained as a colorless solid (115 mg, 91%). ^1^H NMR (400 MHz, DMSO-*d*_6_) δ: 7.56–7.38 (m, 4H), 4.58 (t, *J* = 6.7 Hz, 1H), 3.67 (q, *J* = 4.4 Hz, 2H), 3.61 (dd, *J* = 16.4, 7.1 Hz, 1H), 3.50 (dt, *J* = 21.6, 5.3 Hz, 2H), 3.41 (dd, *J* = 16.4, 6.3 Hz, 1H), 3.19 (t, 1H), 2.60 (s, 3H), 2.56–2.52 (m, 2H), 2.41 (s, 4H), 2.07 (s, 3H), 1.63 (s, 3H). ^13^C NMR (101 MHz, DMSO-*d*_6_) δ: 168.1, 162.9, 155.3, 149.8, 136.8, 135.2, 132.2, 130.7, 130.2, 129.9, 129.6, 128.5, 79.1, 76.0, 54.1, 51.5, 50.9, 46.0, 44.8, 41.0, 40.4, 39.5, 34.8, 14.0, 12.7, 11.3. ESI-MS: *m*/*z* = 507.10 ([M + H]^+^).

##### Synthesis of Methyl 1-(3-Fluorobenzyl)-1*H*-indole-5-carboxylate (**I1**)

To a solution of methylindole-5-carboxylate (2.00 g, 11.2 mmol, 1.0 equiv) and 3-fluorobenzyl chloride (1.78 g, 12.3 mmol, 1.1 equiv) in dry DMF (50 mL) were added NaH (60% dispersion in mineral oil, 0.67 g, 16.8 mmol, 1.5 equiv) and KI (catalytic amount). The mixture was sparged with argon and stirred at rt. for 3 h. Afterward, the reaction was quenched with water (100 mL) and extracted with EtOAc (3 × 100 mL). The combined organic phase was dried over MgSO_4_ and the solvent was removed under reduced pressure. The crude product was purified by flash chromatography (*n*-Hex/EtOAc = 90:10 → 20:80) to afford the title compound as a pale-yellow solid in 57% yield (1.82 g, 6.41 mmol). ^1^H NMR (300 MHz, DMSO-*d*_6_) δ: 8.28 (s, 1H), 7.74 (dd, *J* = 8.7, 1.5 Hz, 1H), 7.65 (d, *J* = 3.2 Hz, 1H), 7.57 (d, *J* = 8.7 Hz, 1H), 7.42–7.29 (m, 1H), 7.15–6.97 (m, 3H), 6.68 (d, *J* = 3.2 Hz, 1H), 5.50 (s, 2H), 3.83 (s, 3H). ESI-MS: *m*/*z* = 284.05 ([M + H]^+^).

##### Synthesis of 1-(3-Fluorobenzyl)-1*H*-indole-5-carboxylic Acid (**I2**)

To a solution **I1** (656 mg, 2.32 mmol, 1.0 equiv) in THF (12.5 mL) and MeOH (12.5 mL) was added dropwise a 0.5 M aqueous solution of KOH (11.60 mol, 5.0 equiv). The mixture was stirred at 70 °C for 16 h. Afterward, the reaction mixture was cooled to rt and the solvent mixture was evaporated. The residue was then acidified with 2 M aqueous HCl (pH = 3) and the resulting aqueous solution was extracted with DCM (3 × 20 mL). The combined organic phase was dried over MgSO_4_ and the solvent was removed under reduced pressure to afford the title compound as a pale-yellow solid in 98% yield (610 mg, 2.27 mmol). ^1^H NMR (250 MHz, DMSO-*d*_6_) δ: 12.45 (br s, 1H), 8.25 (d, *J* = 1.1 Hz, 1H), 7.72 (dd, *J* = 8.7 Hz, *J* = 1.6 Hz, 1H), 7.63 (d, *J* = 3.2 Hz, 1H), 7.54 (d, *J* = 8.7 Hz, 1H), 7.43–7.29 (m, 1H), 7.13–6.98 (m, 3H), 6.66 (dd, *J* = 3.2 Hz, *J* = 0.6 Hz, 1H), 5.49 (s, 2H). ESI-MS: *m*/*z* = 268.10 ([M + H]^+^).

##### Synthesis of *N*-(4-Amino-2-(trifluoromethyl)­benzyl)-1-(3-fluorobenzyl)-1*H*-indole-5-carboxamide (**I3**)

To a solution of **I2** (100 mg, 0.35 mmol, 1.0 equiv) in dry THF (9 mL) were added DIPEA (0.2 mL, 1.06 mmol, 3.0 equiv) and PyBOP (202 mg, 0.39 mmol, 1.1 equiv). The mixture was stirred at rt for 10 min before 4-amino-3-(trifluoromethyl)­benzylamine (69 mg, 0.35 mmol, 1.0 equiv) and HOBt·H_2_O (27 mg, 0.18 mmol, 0.5 equiv) were added. The resulting reaction mixture was stirred at rt for 16 h. Afterward, the mixture was diluted with EtOAc (10 mL) and washed with brine (15 mL). The aqueous phase was extracted with EtOAc (3 × 15 mL) and the combined organic phase was dried over MgSO_4_. After removing the solvent under reduced pressure, the crude product was purified by flash chromatography (*n*-Hex/EtOAc = 2:1 → 1:1) to afford the title compound as a yellow solid in 89% yield (138 mg, 0.31 mmol). ^1^H NMR (250 MHz, DMSO-*d*_6_) δ: 8.70 (t, *J* = 5.7 Hz, 1H), 8.20 (d, *J* = 1.2 Hz, 1H), 7.69 (dd, *J* = 8.7 Hz, *J* = 1.6 Hz, 1H), 7.61 (d, *J* = 3.2 Hz, 1H), 7.52 (d, *J* = 8.7 Hz, 1H), 7.41–7.28 (m, 1H), 7.16 (d, *J* = 8.4 Hz, 1H), 7.13–6.97 (m, 3H), 6.90 (d, *J* = 2.3 Hz, 1H), 6.74 (dd, *J* = 8.3 Hz, *J* = 2.1 Hz, 1H), 6.62 (d, *J* = 3.1 Hz, 1H), 5.49 (s, 2H), 5.43 (s, 2H), 4.49 (d, *J* = 5.4 Hz, 2H). ESI-MS: *m*/*z* = 441.90 ([M + H]^+^).

##### Synthesis of 1-(3-Fluorobenzyl)-*N*-(4-(pent-4-yn-1-ylsulfonamido)-2-(trifluoromethyl)-benzyl)-*1H*-indole-5-carboxamide (**A5**)

**I3** (100 mg, 0.25 mmol, 1.0 equiv) was dissolved in 10 mL dry CHCl_3_ and the solution was sparged with argon. Then, 1-sulfonyl chloride-4-pentyne (52 mg, 0.29 mmol, 1.2 equiv) and pyridine (0.1 mL, 1.09 mmol, 5.0 equiv) were added. The reaction mixture was stirred at 55 °C for 48 h. After cooling to rt the solution was acidified with 2 M aqueous HCl (pH = 3) and extracted with DCM (3 × 15 mL). The combined organic phase was dried over MgSO_4_ and the solvent was removed under reduced pressure. Purification using reversed phase flash chromatography (ACN/H_2_O = 30:70 → 10:90) afforded the title compound as a colorless solid in 74% yield (96 mg, 0.18 mmol). ^1^H NMR (400 MHz, DMSO-*d*_6_) δ: 10.16 (s, 1H, N*H*), 8.92 (t, ^3^ *J*_HH_ = 5.8 Hz, 1H, N*H*), 8.22 (d, *J* = 1.3 Hz, 1H), 7.70 (dd, *J* = 8.7 Hz, *J* = 1.6 Hz, 1H), 7.63 (d, *J* = 3.2 Hz, 1H), 7.54 (d, *J* = 8.9 Hz, 2H), 7.52–7.42 (m, 2H), 7.35 (m, 1H), 7.12–7.05 (m, 1H), 7.04–6.99 (m, 2H), 6.64 (dd, *J* = 3.2 Hz, *J* = 0.6 Hz, 1H), 5.50 (s, 2H), 4.61 (d, *J* = 5.4 Hz, 2H), 3.23–3.17 (m, 2H), 2.76 (t, *J* = 2.6 Hz, 1H), 2.28 (td, *J* = 7.0 Hz, *J* = 2.6 Hz, 2H), 1.82 (m, 2H). ^13^C NMR (126 MHz, DMSO-*d*_6_) δ: 167.4, 158.0, 137.3, 137.2, 130.73, 130.7, 130.6, 130.5, 127.7, 125.5, 122.9, 120.9, 120.6, 120.2, 114.3, 113.9, 113.8, 113.6, 109.8, 102.4, 82.9, 74.9, 72.22, 72.18, 72.1, 49.9, 48.6, 22.4, 16.2. ^19^F NMR (282 MHz, DMSO-*d*_6_) δ: −59.7 (s), −112.1 (s). ESI-MS: *m*/*z* = 572.23 ([M + H]^+^).

##### Synthesis of *tert*-Butyl-((1*S*,3*R*)-3-((4-amino-2-(trifluoromethyl)­benzyl)­carbamoyl)-cyclohexyl)­carbamate (**I4**)

(4-(Aminomethyl)-3-trifluoromethyl)­aniline (200 mg, 0.78 mmol, 1.0 equiv) and PyBOP (447 mg, 0.86 mmol, 1.1 equiv) were dissolved in dry THF (15 mL). DIPEA (0.41 mL, 2.34 mmol, 3.0 equiv), HOBt·H_2_O (60 mg, 0.39 mmol, 0.5 equiv) and (1*R*,3*S*)-3-((*tert*-Butoxycarbonyl)­amino)­cyclohexane-1-carboxylic acid (150 mg, 0.78 mmol, 1.0 equiv) were added and the reaction mixture was stirred at rt for 16 h. Afterward, the mixture was diluted with EtOAc (15 mL) and washed with H_2_O (2 × 15 mL) and brine (15 mL). The aqueous phase was extracted with EtOAc (3 × 15 mL) and the combined organic phase was dried over MgSO_4_. After removing the solvent under reduced pressure, the crude product was purified by flash chromatography (*n*-Hex/EtOAc = 100:0 → 70:30) to afford the title compound as a yellow solid in 82% yield (266 mg, 0.64 mmol). ^1^H NMR (250 MHz, CDCl_3_) δ: 7.27 (d, *J* = 8.2 Hz, 1H), 6.91 (d, *J* = 2.5 Hz, 1H), 6.76 (dd, *J* = 2.4 Hz, *J* = 8.2 Hz, 1H), 5.72 (t, *J* = 5.4 Hz, 1H), 4.45 (s, 2H), 4.43 (s, 1H), 3.85 (s, 2H), 3.48–3.38 (m, 2H), 2.13–2.07 (m, 2H), 1.95–1.90 (m, 2H), 1.86–1.76 (m, 1H), 1.82–1.76 (m, 2H), 1.42 (s, 9H), 1.08–1.02 (m, 1H). ESI-MS: *m*/*z* = 416.15 ([M + H]^+^).

##### Synthesis of *tert*-Butyl­((1*S*,3*R*)-3-((4-(pent-4-yn-1-ylsulfonamido)-2-(trifluoromethyl)-benzyl)­carbamoyl)­cyclohexyl)­carbamate (**I5**)

**I4** (100 mg, 0.24 mmol, 1.0 equiv), Pent-4-yne-1-sulfonyl chloride (48 mg, 0.27 mmol, 1.1 equiv) and Pyridine (0.1 mL, 1.2 mmol, 5.0eq) were dissolved in dry CHCl_3_ (20 mL) and the reaction mixture was stirred at 60 °C for 48 h. The reaction mixture was diluted with EtOAc (15 mL) and washed with 1 M HCl (2 × 20 mL) and brine (20 mL). The aqueous phase was extracted with EtOAc (3 × 20 mL) and the combined organic phase was dried over MgSO_4_. After removing the solvent under reduced pressure, the crude product was purified by flash chromatography (*n*-Hex/EtOAc = 100:0 → 80:20) to afford the title compound as a brown oil in 37% yield (99 mg, 0.18 mmol). ^1^H NMR (400 MHz, CDCl_3_) δ: 8.36 (s, 1H), 7.50 (d, *J* = 1.7 Hz, 1H), 7.36–7.34 (m, 1H), 7.31–7.29 (m, 1H), 6.32–6.28 (m, 1H), 4.56 (dd, *J* = 7.1 Hz, *J* = 17.6 Hz, 2H), 4.46 (dd, *J* = 5.4 Hz, *J* = 15.7 Hz, 1H), 3.47 (s, 2H), 3.24–3.20 (m, 2H), 2.34 (dt, *J* = 2.6 Hz, *J* = 6.7 Hz, 2H), 2.29–2.23 (m, 1H), 2.14–2.11 (m, 1H), 2.02 (q, *J* = 7.2 Hz, 2H), 1.95 (t, *J* = 2.6 Hz, 1H), 1.90–1.86 (m, 1H), 1.86–1.78 (m, 2H), 1.43 (s, 9H), 1.31–1.29 (m, 2H), 1.14–1.04 (m, 1H). ESI-MS: *m*/*z* = 544.05 ([M + H]^+^).

##### Synthesis of (1*R*,3*S*)-3-Amino-*N*-(4-(pent-4-in-1-ylsulfonamido)-2-(trifluoromethyl)-benzyl)­cyclohexane-1-carboxamide (**I6**)

**I5** (83 mg, 0.15 mmol) was dissolved in dry DCM (2.5 mL). The flask was cooled to 0 °C in an ice bath and trifluoroacetic acid (TFA) in an excess was added dropwise. The solution was stirred at rt for two h. The reaction mixture was diluted with saturated aqueous NaHCO_3_ (pH = 8) and the aqueous phase was extracted with EtOAc (5 × 10 mL). The combined organic phase was dried over MgSO_4_. All volatiles were removed under reduced pressure to afford the title compound as a colorless solid in 99% yield (68 mg, 0.15 mmol). ^1^H NMR (400 MHz, MeOH-*d*_4_) δ: 7.56–7.55 (m, 1H), 7.47–7.42 (m, 2H), 4.91 (s,2H), 3.24–3.20 (m, 2H), 3.15 (tt, *J* = 11.7 Hz, *J* = 3.92 Hz, 1H), 2.45 (tt, *J* = 11.8 Hz, *J* = 3.5 Hz, 1H), 2.31 (td, *J* = 6.9 Hz, *J* = 2.3 Hz, 2H), 2.21 (t, *J* = 2.6 Hz, 1H), 2.12–2.01 (m, 2H), 1.98–1.90 (m, 2H), 1.94–1.86 (m, 2H), 1.47–1.27 (m, 4H). ESI-MS: *m*/*z* = 446.20 ([M + H]^+^).

##### Synthesis of (1*R*,3*S*)-3-((4-Methyl-6-(methylamino)-1,3,5-triazin-2-yl)­amino)-*N*-(4-(pent-4-yn-1-ylsulfonamido)-2-(trifluoromethyl)­benzyl)­cyclohexane-1-carboxamide (**A6**)

2,4-Dichloro-6-methyl-1,3,5-triazine (26 mg, 0.15 mmol, 1 equiv) and methylamine (17 μL, 0.15 mmol, 1 equiv) were combined in a 5 mL round-bottom flask and mixed with 1 M sodium hydroxide solution until a pH of 12 was reached. A solution of **I6** (68 mg, 0.15 mmol, 1 equiv) in 1 mL of methanol was added and the pH was adjusted to 10 with 1 M sodium hydroxide solution. The reaction mixture was stirred at 90 °C for 16 h. The solvent was removed under reduced pressure and the crude product was purified by flash chromatography (DCM/MeOH = 100:0 → 90:10) to afford the title compound as a yellow solid in 20% yield (15 mg, 13 μmol). ^1^H NMR (400 MHz, CD_2_Cl_2_) δ: 7.56 (d, *J* = 2.0 Hz, 1H), 7.39 (d, *J* = 8.4 Hz, 1H), 7.33–7.30 (m, 1H), 4.58–4.47 (m, 2H), 3.99–3.97 (m, 1H), 3.27–3.20 (m, 2H), 2.90–2.86 (m, 2H), 2.33 (dt, *J* = 2.6 Hz, *J* = 6.8 Hz, 3H), 2.17–3.13 (m, 2H), 2.04–1.97 (m, 4H), 1.86–1.80 (m, 2H), 1.40–1.39 (m, 2H), 1.26 (s, 3H), 1.63 (d, *J* = 6.1 Hz, 1H), 0.89–0.84 (m, 1H). ESI-MS: *m*/*z* = 568.35 ([M + H]^+^).

##### Synthesis of *tert*-Butyl 2-((2-(2,6-Dioxopiperidin-3-yl)-1,3-dioxoisoindolin-4-yl)­oxy)­acetate (**A7**)

The title compound was prepared according to general procedure A, using 4-(((1*r*,4*r*)-4-(3-(4-(trifluoromethoxy)­phenyl)­ureido)­cyclohexyl)­oxy)­benzoic acid (20 mg, 0.046 mmol), pent-4-yn-1-amine (5.5 mg, 0.046 mmol.), HATU (23 mg, 0.059 mmol) and DIPEA (24 μL, 0.14 mmol). The title compound was obtained as a colorless solid (13.4 mg, 54%). ^1^H NMR (300 MHz, DMSO-*d*_6_) δ: 8.51 (s, 1H), 8.30 (t, *J* = 5.5 Hz, 1H), 7.82–7.76 (m, 2H), 7.49–7.45 (m, 2H), 7.24–7.19 (m, 2H), 7.01–6.96 (m, 2H), 6.19 (d, *J* = 7.4 Hz, 1H), 4.46–4.40 (m, 1H), 3.58–3.48 (m, 1H), 3.30–3.25 (m, 2H), 2.78 (t, *J* = 2.6 Hz, 1H), 2.24–2.17 (m, 2H), 2.07–1.92 (m, 4H), 1.74–1.64 (m, 2H), 1.55–1.31 (m, 4H). ^19^F NMR (282 MHz, DMSO-*d*_6_) δ: −57.1 (s). ESI-MS: *m*/*z* = 504.25 ([M + H]^+^).

##### Synthesis of *tert*-Butyl 2-((2-(2,6-Dioxopiperidin-3-yl)-1,3-dioxoisoindolin-4-yl)­oxy)­acetate (**A8**)

The title compound was prepared according to general procedure A, using 4-(((1*r*,4*r*)-4-(3-(4-(trifluoromethoxy)­phenyl)­ureido)­cyclohexyl)­oxy)­benzoic acid (20 mg, 0.046 mmol), 3-butyn-1-amine hydrochloride (4.8 mg, 0.046 mmol.), HATU (23 mg, 0.059 mmol) and DIPEA (24 μL, 0.14 mmol). The title compound was obtained as a colorless solid (11.3 mg, 49%). ^1^H NMR (300 MHz, DMSO-*d*_6_) δ: 8.52 (s, 1H), 8.46 (t, *J* = 5.5 Hz, 1H), 7.82–7.77 (m, 2H), 7.50–7.45 (m, 2H), 7.25–7.19 (m, 2H), 7.04–6.99 (m, 2H), 6.20 (d, *J* = 7.6 Hz, 1H), 4.47–4.41 (m, 1H), 3.55–3.51 (m, 1H), 3.39–3.37 (m, 2H), 2.82 (t, *J* = 2.5 Hz, 1H), 2.41 (dt, *J* = 2.4 Hz, 7.1 Hz, 2H), 2.08–1.92 (m, 4H), 1.55–1.31 (m, 4H). ^19^F NMR (282 MHz, DMSO-*d*_6_) δ: −57.1 (s). ESI-MS: *m*/*z* = 490.15 ([M + H]^+^).

##### Synthesis of *N*-(2′-Fluoro-5′-(prop-2-yn-1-ylcarbamoyl)-4-((3*S*,5*R*)-3,4,5-trimethylpiperazin-1-yl)-[1,1′-biphenyl]-3-yl)-6-oxo-4-(trifluoromethyl)-1,6-Dihydropyridine-3-carboxamide (**A9**)

The title compound was prepared according to general procedure A, using 6-fluoro-3′-(6-oxo-4-(trifluoromethyl)-1,6-dihydropyridine-3-carboxamido)-4′-((3*S*,5*R*)-3,4,5-trimethylpiperazin-1-yl)-[1,1′-biphenyl]-3-carboxylic acid (10 mg, 18 μmol), prop-2-yn-1-amine (1.0 mg, 18 μmol), HATU (9.1 mg, 24 μmol) and DIPEA (2.2 μL, 55 μmol). The title compound was obtained as a colorless solid (10.4 mg, 97%). ^1^H NMR (400 MHz, DMSO-*d*_6_) δ: 12.58 (s, 1H), 9.55 (s, 1H), 9.06 (t, *J* = 5.5 Hz, 1H), 8.16 (s, 1H), 8.02–7.97 (m, 2H), 7.94–7.87 (m, 1H), 7.50–7.39 (m, 2H), 7.33 (d, *J* = 8.3 Hz, 1H), 6.83 (s, 1H), 4.07 (dd, *J* = 5.5, 2.5 Hz, 2H), 3.46 (d, *J* = 14.5 Hz, 2H), 3.36 (s, 4H), 3.14 (t, *J* = 2.5 Hz, 1H), 2.89–2.82 (m, 3H), 1.33 (d, *J* = 6.3 Hz, 6H). ^13^C NMR (101 MHz, DMSO-*d*_6_) δ: 164.8, 162.9, 161.8, 161.1, 159.8, 142.2, 138.5, 132.3, 130.6, 130.0, 128.8, 127.7, 125.8, 124.0, 123.1, 121.0, 120.6, 116.5, 116.3, 111.5, 89.5, 81.2, 73.0, 59.8, 55.6, 48.6, 36.5, 29.0, 28.6, 14.6. ESI-MS: *m*/*z* = 584.20 ([M + H]^+^).

##### Synthesis of *N*-(2′-Fluoro-5′-((2-(prop-2-yn-1-yloxy)­ethyl)­carbamoyl)-4-((3*S*,5*R*)-3,4,5-trimethylpiperazin-1-yl)-[1,1′-biphenyl]-3-yl)-6-oxo-4-(trifluoromethyl)-1,6-dihydropyridine-3-carboxamide (**A10**)

The title compound was prepared according to general procedure A, using 6-fluoro-3′-(6-oxo-4-(trifluoromethyl)-1,6-dihydropyridine-3-carboxamido)-4′-((3*S*,5*R*)-3,4,5-trimethylpiperazin-1-yl)-[1,1′-biphenyl]-3-carboxylic acid (10 mg, 18 μmol), 2-(prop-2-yn-1-yloxy)­ethan-1-amine (1.8 mg, 18 μmol), HATU (9.1 mg, 24 μmol) and DIPEA (2.2 μL, 55 μmol). The title compound was obtained as a colorless solid (6.8 mg, 60%). ^1^H NMR (400 MHz, DMSO-*d*_6_) δ: 12.58–11.21 (m, 1H), 9.53 (s, 1H), 8.68 (t, *J* = 5.9 Hz, 1H), 8.01–7.95 (m, 2H), 7.92–7.86 (m, 1H), 7.47–7.38 (m, 2H), 7.31 (s, 1H), 6.83 (s, 1H), 4.17 (d, *J* = 2.4 Hz, 1H), 3.64–3.51 (m, 4H), 3.49–3.41 (m, 4H), 3.34 (s, 4H), 2.90 (s, 4H), 1.23 (s, 6H). ^13^C NMR (101 MHz, DMSO-*d*_6_) δ: 165.2, 162.8, 161.5, 161.1, 159.6, 131.1, 130.4, 129.8, 129.6, 128.6, 127.7, 127.6, 126.2, 125.9, 123.1, 120.9, 116.3, 116.1, 111.3, 108.5, 80.3, 77.5, 77.2, 67.8, 67.7, 57.6, 57.4, 44.0, 28.4, 27.2, 13.9. ESI-MS: *m*/*z* = 628.20 ([M + H]^+^).

##### Synthesis of *N*-(2′-Fluoro-5′-(4-(prop-2-yn-1-yl)­piperazine-1-carbonyl)-4-((3*S*,5*R*)-3,4,5-trimethylpiperazin-1-yl)-[1,1′-biphenyl]-3-yl)-6-oxo-4-(trifluoromethyl)-1,6-dihydropyridine-3-carboxamide (**A11**)

The title compound was prepared according to general procedure A, using 6-fluoro-3′-(6-oxo-4-(trifluoromethyl)-1,6-dihydropyridine-3-carboxamido)-4′-((3*S*,5*R*)-3,4,5-trimethylpiperazin-1-yl)-[1,1′-biphenyl]-3-carboxylic acid (10 mg, 18 μmol), 1-(prop-2-yn-1-yl)­piperazine (2.3 mg, 18 μmol), HATU (9.1 mg, 24 μmol) and DIPEA (2.2 μL, 55 μmol). The title compound was obtained as a colorless solid (11.2 mg, 94%). ^1^H NMR (400 MHz, CD_2_Cl_2_) δ: 8.92 (s, 1H), 8.51 (s, 1H), 7.82 (s, 1H), 7.52 (dd, *J* = 7.4, 2.2 Hz, 1H), 7.43–7.35 (m, 1H), 7.30 (q, *J* = 8.3 Hz, 2H), 7.19 (dd, *J* = 10.3, 8.4 Hz, 1H), 6.88 (s, 1H), 3.76 (s, 2H), 3.51 (s, 2H), 3.34 (d, *J* = 2.5 Hz, 2H), 2.85 (d, *J* = 11.2 Hz, 2H), 2.74 (t, *J* = 11.0 Hz, 2H), 2.55 (s, 6H), 2.35 (s, 3H), 2.33–2.29 (m, 1H), 1.13 (s, 3H), 1.12 (s, 3H). ^13^C NMR (101 MHz, CD_2_Cl_2_) δ: 169.6, 163.4, 162.5, 161.8, 159.8, 141.9, 140.4, 140.1, 138.4, 134.0, 133.0, 132.6, 130.5, 129.5, 129.4, 128.6, 125.9, 123.5, 121.5, 121.1, 120.5, 116.9, 116.7, 114.5, 78.9, 73.8, 59.4, 59.3, 47.2, 17.8. ESI-MS: *m*/*z* = 653.20 ([M + H]^+^).

##### Synthesis of 3-Bromo-5-(bromomethyl)­benzoic Acid (**I7**)

3-Bromo-5-methylbenzoic acid (2.0 g, 9.3 mmol), *N*-bromsuccinimid (3.15 g, 17.7 mmol) and benzoyl peroxide (0.11 g, 0.47 mmol) were suspended in dry acetonitrile (120 mL) and stirred at 80 °C for 18 h. Upon cooling, the mixture was concentrated in vacuo and subsequently purified by flash chromatography using acetonitrile/water to afford the title compound as a white solid (1.63 g, 60%). ^1^H NMR (300 MHz, DMSO-*d*_6_) δ: 8.01 (t, *J* = 1.5 Hz, 1H), 7.95 (dt, *J* = 7.0, 1.9 Hz, 2H), 4.78 (s, 2H).

##### Synthesis of 3-((1*H*-Imidazole-1-yl)­methyl)-5-bromobenzoic Acid (**I8**)

Imidazole (40 mg, 0.59 mmol) was dissolved in dry 1,4-dioxane (5 mL). A solution of **I7** (87 mg, 0.30 mmol) in dry 1,4-dioxane (4 mL) was added dropwise. After stirring for 3 h at 75 °C, the mixture was concentrated in vacuo and purified by flash chromatography using acetonitrile/water as an eluent to afford the title compound as a white solid (51 mg, 61%). ^1^H NMR (400 MHz, DMSO-*d*_6_) δ: 9.12 (s, 1H), 8.03 (d, *J* = 1.4 Hz, 1H), 7.97 (dt, *J* = 11.7, 1.6 Hz, 2H), 7.71 (d, *J* = 64.1 Hz, 2H), 5.49 (s, 2H). ESI-MS: *m*/*z* = 282.95 ([M + H]^+^).

##### Synthesis of 3-((1*H-*Imidazole-1-yl)­methyl)-5-(1-ethyl-3-(trifluoromethyl)-*1H*-pyrazol-4-yl)­benzoic Acid (**I9**)

**I8** (250 mg, 0.895 mmol, 1.0 equiv), (1-ethyl-3-(trifluoromethyl)-1*H*-pyrazol-4-yl)­boronic acid (370 mg, 1.78 mmol, 2.0 equiv), K_3_PO_3_ (475 mg, 2.23 mmol, 2.5 equiv) and XPhos Pd G2 (35 mg, 0.045 mmol, 0.05 equiv) were dissolved in 1,4-dioxane (8 mL) and H_2_O (2 mL). The reaction mixture was stirred at 80 °C for 3 h. Then, the reaction mixture was diluted with H_2_O (20 mL) and extracted with CH_2_Cl_2_ (3 × 20 mL). The combined organic phase was washed with brine (20 mL), dried over MgSO_4_, filtered, and concentrated under reduced pressure. The crude product was purified by flash chromatography using acetonitrile/water as an eluent to afford the title compound (301 mg, 92%). ^1^H NMR (600 MHz, DMSO-*d*_6_) δ: 9.29 (s, 1H), 8.32 (s, 1H), 7.98 (dd, *J* = 7.6, 4.8 Hz, 2H), 7.84 (t, *J* = 1.7 Hz, 1H), 7.72 (t, *J* = 1.6 Hz, 1H), 7.66 (s, 1H), 5.56 (s, 2H), 4.26 (q, *J* = 7.3 Hz, 2H), 1.45 (t, *J* = 7.3 Hz, 3H). ESI-MS: *m*/*z* = 365.05 ([M + H]^+^).

##### Synthesis of *tert*-Butyl (*S*)-(1-(6,7-Dihydrothieno­[3,2-*c*]­pyridin-5­(4*H*)-yl)-1-oxopent-4-yn-2-yl)­carbamate (**I10**)

The title compound was prepared according to general procedure A, using (*S*)-2-((*tert*-butoxycarbonyl)­amino)­pent-4-ynoic acid (500 mg, 2.34 mmol), 4,5,6,7-tetrahydrothieno­[3,2-*c*]­pyridine hydrochloride (412 mg, 2.34 mmol), HATU (1.16 g, 3.04 mmol) and DIPEA (1.22 mL, 7.02 mmol). The title compound was obtained as a colorless solid (715 mg, 91%). ^1^H NMR (400 MHz, CDCl_3_) δ: 7.14 (d, *J* = 5.2 Hz, 1H), 6.79 (dd, *J* = 13.1, 5.1 Hz, 1H), 5.47 (dd, *J* = 26.2, 8.8 Hz, 1H), 4.91 (s, 1H), 4.70 (ddd, *J* = 42.8, 26.5, 16.7 Hz, 2H), 4.10–3.75 (m, 2H), 2.90 (dd, *J* = 20.1, 14.6 Hz, 2H), 2.74–2.53 (m, 2H), 1.58 (s, 1H), 1.44 (s, *J* = 3.8 Hz, 9H). ESI-MS: *m*/*z* = 357.05 ([M + H]^+^).

##### Synthesis of (*S*)-3-((1*H*-Imidazole-1-yl)­methyl)-*N*-(1-(6,7-dihydrothieno­[3,2-*c*]­pyridin-5­(4*H*)-yl)-1-oxopent-4-yn-2-yl)-5-(1-ethyl-3-(trifluoromethyl)-1*H*-pyrazol-4-yl)­benzamide (**A12**)

**I10** (333 mg, 0.996 mmol) was dissolved in dry DCM (14 mL). TFA (6 mL) was added and the reaction mixture was stirred at rt for 1 h. All volatiles were removed under reduced pressure and the crude product was dissolved in dry DMF (20 mL). **I9** (363 mg, 0.996 mmol), HATU (492 mg, 1.29 mmol) and DIPEA (520 μL, 2.99 mmol) were added to the solution and stirred at rt for two h. The crude product was purified by flash chromatography using acetonitrile/water as an eluent to afford the title compound as a light brown solid (307 mg, 53%). ^1^H NMR (600 MHz, DMSO-*d*_6_) δ: 8.93 (dd, *J* = 73.5, 8.4 Hz, 1H), 8.20 (d, *J* = 11.9 Hz, 1H), 7.89–7.71 (m, 3H), 7.41–7.27 (m, 2H), 7.19 (d, *J* = 6.3 Hz, 1H), 6.94–6.80 (m, 2H), 5.28 (d, *J* = 11.2 Hz, 2H), 5.18 (ddd, *J* = 44.8, 15.1, 8.0 Hz, 1H), 4.75–4.43 (m, 2H), 4.25 (q, *J* = 7.3 Hz, 2H), 3.94–3.69 (m, 2H), 2.92–2.58 (m, 5H), 1.43 (dd, *J* = 8.4, 6.2 Hz, 3H). ^13^C NMR (151 MHz, DMSO-*d*_6_) δ: 168.5, 165.2, 138.4, 136.3, 134.4, 133.1, 132.4, 131.9, 131.5, 131.4, 130.2, 126.8, 126.0, 125.3, 124.4, 123.6, 122.6, 120.8, 119.8, 80.9, 72.6, 49.2, 48.5, 47.2, 43.0, 42.6, 25.1, 21.4, 15.0. ESI-MS: *m*/*z* = 581.15 ([M + H]^+^).

##### Synthesis of *N*-(But-3-yn-1-yl)-4-(3-chloro-2-fluorophenoxy)-1-((6-(thiazol-2-ylamino)­pyridin-2-yl)­methyl)­cyclohexane-1-carboxamide (**A13**)

The title compound was prepared according to general procedure A, using 4-(3-chloro-2-fluorophenoxy)-1-((6-(thiazol-2-ylamino)­pyridin-2-yl)­methyl)­cyclohexane-1-carboxylic acid (10 mg, 22 μmol), but-3-yn-1-amine (1.5 mg, 22 μmol), HATU (10.7 mg, 28.1 μmol) and DIPEA (11.3 μL, 65.9 μmol). The title compound was obtained as a colorless solid (10.1 mg, 91%). ^1^H NMR (500 MHz, DMSO-*d*_6_) δ: 11.12 (s, 1H), 7.78 (t, *J* = 5.7 Hz, 1H), 7.55 (d, *J* = 7.8 Hz, 1H), 7.36 (d, *J* = 3.6 Hz, 1H), 7.18–7.13 (m, 1H), 7.10 (s, 1H), 7.09 (d, *J* = 3.2 Hz, 1H), 6.95 (d, *J* = 3.6 Hz, 1H), 6.86 (d, *J* = 8.2 Hz, 1H), 6.62 (d, *J* = 7.3 Hz, 1H), 4.55 (s, 1H), 3.14 (q, *J* = 7.1, 5.4 Hz, 2H), 2.89 (s, 2H), 2.79 (t, *J* = 2.6 Hz, 1H), 2.26 (td, *J* = 7.2, 2.7 Hz, 2H), 1.93–1.87 (m, 2H), 1.83–1.74 (m, 4H), 1.72–1.63 (m, 2H). ^13^C NMR (125 MHz, DMSO-*d*_6_) δ: 174.2, 159.9, 155.1, 150.8, 149.9, 148.0, 146.2, 137.6, 137.4, 125.0, 121.9, 120.5, 120.4, 116.7, 116.3, 110.7, 108.2, 82.5, 74.3, 72.0, 46.7, 38.1, 28.3, 26.6, 18.6. ESI-MS: *m*/*z* = 513.10 ([M + H]^+^).

##### Synthesis of 4-(3-Chloro-2-fluorophenoxy)-*N*-(hex-5-yn-1-yl)-1-((6-(thiazol-2-ylamino)­pyridin-2-yl)­methyl)­cyclohexane-1-carboxamide (**A14**)

The title compound was prepared according to general procedure A, using 4-(3-chloro-2-fluorophenoxy)-1-((6-(thiazol-2-ylamino)­pyridin-2-yl)­methyl)­cyclohexane-1-carboxylic acid (10 mg, 22 μmol), hex-5-yn-1-amine (2.1 mg, 22 μmol), HATU (10.7 mg, 28.1 μmol) and DIPEA (11.3 μL, 65.9 μmol). The title compound was obtained as a colorless solid (7.1 mg, 61%). ^1^H NMR (500 MHz, DMSO-*d*_6_) δ: 11.11 (s, 1H), 7.60 (t, *J* = 5.6 Hz, 1H), 7.54 (dd, *J* = 8.3, 7.3 Hz, 1H), 7.35 (d, *J* = 3.6 Hz, 1H), 7.21–7.13 (m, 1H), 7.10 (s, 1H), 7.09 (d, *J* = 3.0 Hz, 1H), 6.95 (d, *J* = 3.6 Hz, 1H), 6.86 (d, *J* = 8.2 Hz, 1H), 6.59 (d, *J* = 7.3 Hz, 1H), 4.55 (s, 1H), 3.03 (q, *J* = 6.5 Hz, 2H), 2.90 (s, 2H), 2.75 (t, *J* = 2.6 Hz, 1H), 2.14 (td, *J* = 6.9, 2.7 Hz, 2H), 1.94–1.87 (m, 2H), 1.84–1.74 (m, 4H), 1.69–1.61 (m, 2H), 1.51–1.42 (m, 2H), 1.42–1.33 (m, 2H). ^13^C NMR (125 MHz, DMSO-*d*_6_) δ: 174.0, 159.9, 155.3, 150.8, 149.9, 148.0, 146.2, 137.57, 137.4, 125.0, 121.9, 120.5, 120.4, 116.7, 116.2, 110.7, 108.1, 84.5, 74.4, 71.3, 46.6, 38.4, 28.4, 28.3, 26.7, 25.6, 17.5. ESI-MS: *m*/*z* = 541.15 ([M + H]^+^).

##### Synthesis of 4-(3-Chloro-2-fluorophenoxy)-*N*-(2-(prop-2-yn-1-yloxy)­ethyl)-1-((6-(thiazol-2-ylamino)­pyridin-2-yl)­methyl)­cyclohexane-1-carboxamide (**A15**)

The title compound was prepared according to general procedure A, using 4-(3-chloro-2-fluorophenoxy)-1-((6-(thiazol-2-ylamino)­pyridin-2-yl)­methyl)­cyclohexane-1-carboxylic acid (10 mg, 22 μmol), 2-(prop-2-yn-1-yloxy)­ethan-1-amine (2.2 mg, 22 μmol), HATU (10.7 mg, 28.1 μmol) and DIPEA (11.3 μL, 65.9 μmol). The title compound was obtained as a colorless solid (7.6 mg, 65%). ^1^H NMR (500 MHz, DMSO-*d*_6_) δ: 11.11 (s, 1H), 7.70 (t, *J* = 5.7 Hz, 1H), 7.55 (dd, *J* = 8.2, 7.3 Hz, 1H), 7.35 (d, *J* = 3.6 Hz, 1H), 7.17–7.13 (m, 1H), 7.10 (s, 1H), 7.09 (d, *J* = 3.0 Hz, 1H), 6.95 (d, *J* = 3.6 Hz, 1H), 6.86 (d, *J* = 8.1 Hz, 1H), 6.63 (dd, *J* = 7.4, 0.8 Hz, 1H), 4.55 (s, 1H), 4.12 (d, *J* = 2.4 Hz, 2H), 3.44 (t, *J* = 2.4 Hz, 1H), 3.42 (d, *J* = 6.0 Hz, 2H), 3.21 (q, *J* = 6.0 Hz, 2H), 2.90 (s, 2H), 1.91–1.85 (m, 2H), 1.84–1.73 (m, 4H), 1.70–1.61 (m, 2H). ^13^C NMR (125 MHz, DMSO-*d*_6_) δ: 74.2, 159.8, 155.1, 150.7, 149.9, 147.9, 146.3, 146.2, 137.6, 137.4, 125.0, 121.8, 120.5, 120.4, 116.7, 116.2, 110.7, 108.1, 80.3, 77.2, 74.3, 67.7, 57.3, 46.6, 38.6, 28.3, 26.6. ESI-MS: *m*/*z* = 543.15 ([M + H]^+^).

##### Synthesis of 4-(3-Chloro-2-fluorophenoxy)-*N*-(2-(2-(prop-2-yn-1-yloxy)­ethoxy)­ethyl)-1-((6-(thiazol-2-ylamino)­pyridin-2-yl)­methyl)­cyclohexane-1-carboxamide (**A16**)

The title compound was prepared according to general procedure A, using 4-(3-chloro-2-fluorophenoxy)-1-((6-(thiazol-2-ylamino)­pyridin-2-yl)­methyl)­cyclohexane-1-carboxylic acid (10 mg, 22 μmol), 2-(2-(prop-2-yn-1-yloxy)­ethoxy)­ethan-1-amine (3.1 mg, 22 μmol), HATU (10.7 mg, 28.1 μmol) and DIPEA (11.3 μL, 65.9 μmol). The title compound was obtained as a colorless solid (7.4 mg, 58%). ^1^H NMR (500 MHz, DMSO-*d*_6_) δ: 11.11 (s, 1H), 7.64 (t, *J* = 5.6 Hz, 1H), 7.54 (dd, *J* = 8.2, 7.3 Hz, 1H), 7.35 (d, *J* = 3.6 Hz, 1H), 7.19–7.13 (m, 1H), 7.11 (s, 1H), 7.09 (d, *J* = 2.9 Hz, 1H), 6.95 (d, *J* = 3.6 Hz, 1H), 6.86 (d, *J* = 7.9 Hz, 1H), 6.63 (d, *J* = 7.2 Hz, 1H), 4.55 (s, 1H), 4.13 (d, *J* = 2.4 Hz, 2H), 3.61–3.47 (m, 4H), 3.40 (t, *J* = 2.4 Hz, 1H), 3.38–3.32 (m, 2H), 3.19 (q, *J* = 6.0 Hz, 2H), 2.90 (s, 2H), 1.94–1.86 (m, 2H), 1.84–1.74 (m, 4H), 1.71–1.62 (m, 2H). ^13^C NMR (125 MHz, DMSO-*d*_6_) δ: 174.2, 159.8, 155.2, 150.7, 149.9, 147.9, 146.3, 146.2, 137.6, 137.4, 125.0, 124.9, 121.8, 120.5, 120.4, 116.7, 116.2, 110.7, 108.1, 80.3, 77.5, 74.7, 69.2, 68.8, 68.5, 57.5, 46.7, 38.7, 28.4, 26.6. ESI-MS: *m*/*z* = 587.15 ([M + H]^+^).

##### Synthesis of *tert*-Butyl 2-((2-(2,6-Dioxopiperidin-3-yl)-1,3-dioxoisoindolin-4-yl)­oxy)­acetate (**I11**)

2-(2,6-Dioxopiperidin-3-yl)-4-hydroxyisoindoline-1,3-dione (1.00 g, 3.65 mmol, 1.0 equiv) and potassium carbonate (1.26 g, 9.12 mmol, 3.0 equiv) were suspended in dry DMF (15 mL). The flask was cooled to 0 °C in an ice bath and *tert*-butyl bromoacetate (538 μL, 3.65 mmol, 1.0 equiv) was added dropwise through a dropping funnel over 30 min. The solution was warmed to ambient temperature and stirred for two h. The reaction mixture was diluted with cold water and the precipitate was filtered. The colorless solid was dried under reduced pressure to obtain the title compound (1.21 g, 85%). ^1^H NMR (400 MHz, DMSO-*d*_6_) δ: 11.11 (s, 1H), 7.80 (t, *J* = 7.9 Hz, 1H), 7.48 (d, *J* = 7.3 Hz, 1H), 7.37 (d, *J* = 8.5 Hz, 1H), 5.10 (dd, *J* = 13.0, 5.3 Hz, 1H), 4.96 (s, 2H), 2.89 (t, *J* = 19.1, 14.0, 5.3 Hz, 1H), 2.58 (t, 2H), 2.15–1.92 (m, 1H), 1.42 (s, 9H). ESI-MS: *m*/*z* = 411.10 ([M + Na]^+^).

##### Synthesis of 2-((2-(2,6-Dioxopiperidin-3-yl)-1,3-dioxoisoindolin-4-yl)­oxy)­acetic Acid (**I12**)

**I11** (1.00 g, 3.02 mmol) was dissolved in dry DCM (5 mL). The flask was cooled to 0 °C in an ice bath and trifluoroacetic acid (TFA) in an excess was added dropwise. The solution was stirred at rt for two h. The solvent was removed under reduced pressure and the crude product used without further purification. ESI-MS: *m*/*z* = 333.10 ([M + H]^+^).

##### Synthesis of *N*-(3-Azidopropyl)-2-((2-(2,6-dioxopiperidin-3-yl)-1,3-dioxoisoindolin-4-yl)-oxy)­acetamide (**X1**)

The title compound was prepared according to general procedure A, using **I12** (100 mg, 0.304 mmol), 3-azidopropan-1-amine (30 mg, 0.30 mmol), HATU (150 mg, 0.395 mmol) and DIPEA (158 μL, 0.912 mmol). The title compound was obtained as a light yellow solid (114 mg, 91%). ^1^H NMR (400 MHz, DMSO-*d*_6_) δ: 11.11 (s, 1H), 8.05 (t, *J* = 5.8 Hz, 1H), 7.81 (dd, *J* = 8.5, 7.3 Hz, 1H), 7.50 (d, *J* = 7.2 Hz, 1H), 7.39 (d, *J* = 8.5 Hz, 1H), 5.12 (dd, *J* = 12.9, 5.4 Hz, 1H), 4.78 (s, 2H), 3.37 (t, *J* = 6.8 Hz, 2H), 3.22 (q, *J* = 6.5 Hz, 2H), 2.90 (m, 1H), 2.63–2.51 (m, 2H), 2.16–1.99 (m, 1H), 1.69 (quin, *J* = 6.8 Hz, 2H). ^13^C NMR (101 MHz, DMSO-*d*_6_) δ: 172.8, 169.9, 166.7, 166.7, 165.5, 155.1, 136.9, 133.1, 120.4, 116.8, 116.1, 67.7, 48.8, 48.3, 35.8, 30.9, 28.3, 22.0. ESI-MS: *m*/*z* = 437.10 ([M + Na]^+^).

##### Synthesis of *N*-(2-(2-(2-Azidoethoxy)­ethoxy)­ethyl)-2-((2-(2,6-dioxopiperidin-3-yl)-1,3-dioxoisoindolin-4-yl)­oxy)­acetamide (**X2**)

The title compound was prepared according to general procedure B, using **I12** (350 mg, 1.05 mmol) and 1-(2-aminoethoxy)-2-(2-azidoethoxy)­ethane (219 mg, 1.26 mmol), 1-methyl-1*H*-imidazole (293 μL, 3.68 mmol) and TCFH (353 mg, 1.26 mmol). The title compound was obtained as a colorless solid (315 mg, 62%). ^1^H NMR (500 MHz, acetone-*d*_6_) δ: 9.95 (s, 1H), 7.87 (dd, *J* = 8.4, 7.3 Hz, 1H), 7.63 (s, 1H), 7.55–7.48 (m, 2H), 5.19–5.11 (m, 1H), 4.75 (s, 2H), 3.71–3.56 (m, 8H), 3.48 (q, *J* = 5.5 Hz, 2H), 3.35 (t, *J* = 5.0 Hz, 2H), 3.04–2.93 (m, 1H), 2.83–2.74 (m, 2H), 2.29–2.19 (m, 1H). ^13^C NMR (126 MHz, acetone-*d*_6_) δ: 171.7, 169.1, 166.7, 166.6, 165.9, 154.9, 137.0, 133.7, 120.3, 117.8, 116.3, 70.3, 70.2, 69.9, 69.3, 69.3, 68.0, 50.5, 49.3, 38.7, 31.1, 29.4, 29.3, 29.1, 29.0, 28.8, 28.6, 28.5, 22.4. ESI-MS: *m*/*z* = 489.2 ([M + H]^+^).

##### Synthesis of *N*-(3-Azidopropyl)-2-((2-(2,6-dioxopiperidin-3-yl)-1,3-dioxoisoindolin-4-yl)-oxy)­acetamide (**X3**)

The title compound was prepared according to general procedure B, using **I12** (360 mg, 1.08 mmol) and 14-azido-3,6,9,12-tetraoxatetradecan-1-amine (283 μL, 1.19 mmol), 1-methyl-1*H*-imidazole (301 μL, 3.78 mmol) and TCFH (363 mg, 1.30 mmol). The title compound was obtained as a white solid (460 mg, 73%). ^1^H NMR (400 MHz, CD_2_Cl_2_) δ: 8.56 (s, 1H), 7.67 (dd, *J* = 8.4, 7.3 Hz, 1H), 7.49–7.42 (m, 2H), 7.15 (dd, *J* = 8.4, 0.7 Hz, 1H), 4.92–4.82 (m, 1H), 4.56 (s, 2H), 3.60–3.49 (m, 16H), 3.45 (quin, *J* = 5.2 Hz, 2H), 3.29 (t, 2H), 2.81–2.60 (m, 3H), 2.13–2.02 (m, 1H). ^13^C NMR (101 MHz, CD_2_Cl_2_) δ: 171.0, 168.3, 166.7, 166.6, 165.9, 154.5, 136.9, 133.7, 119.5, 118.0, 117.0, 70.6, 70.5, 70.4, 70.3, 69.8, 69.5, 68.1, 50.8, 49.3, 39.0, 31.4, 22.6. ESI-MS: *m*/*z* = 577.2 ([M + H]^+^).

##### Synthesis of 4-((3-Azidopropyl)­amino)-2-(2,6-dioxopiperidin-3-yl)­isoindoline-1,3-dione (**X4**)

The title compound was prepared according to general procedure C, using 2-(2,6-dioxopiperidin-3-yl)-4-fluoroisoindoline-1,3-dione (150 mg, 0.543 mmol), 3-azidopropan-1-amine (65 mg, 0.65 mmol) and DIPEA (283 μL, 1.63 mmol). The title compound was obtained as a yellow solid (155 mg, 80%). ^1^H NMR (400 MHz, DMSO-*d*_6_) δ: 11.09 (s, 1H), 7.59 (dd, *J* = 8.6, 7.1 Hz, 1H), 7.11 (d, *J* = 8.6 Hz, 1H), 7.03 (d, *J* = 7.0 Hz, 1H), 6.67 (t, *J* = 6.1 Hz, 1H), 5.05 (dd, *J* = 12.9, 5.4 Hz, 1H), 3.45 (t, *J* = 6.7 Hz, 2H), 3.38 (q, *J* = 6.7 Hz, 2H), 2.95–2.81 (m, 1H), 2.64–2.53 (m, 2H), 2.08–1.94 (m, 1H), 1.83 (quin, *J* = 6.8 Hz, 2H). ^13^C NMR (101 MHz, DMSO-*d*_6_) δ: 172.8, 170.1, 168.8, 167.3, 146.2, 136.3, 132.2, 117.2, 110.5, 109.3, 48.1, 39.2, 30.9, 27.9, 22.1. ESI-MS: *m*/*z* = 357.15 ([M + H]^+^).

##### Synthesis of 4-((4-Azidobutyl)­amino)-2-(2,6-dioxopiperidin-3-yl)­isoindoline-1,3-dione (**X5**)

The title compound was prepared according to general procedure C, using 2-(2,6-dioxopiperidin-3-yl)-4-fluoroisoindoline-1,3-dione (200 mg, 0.724 mmol), 4-azidobutan-1-amine (99 mg, 0.87 mmol) and DIPEA (378 μL, 2.17 mmol). The title compound was obtained as a yellow solid (196 mg, 73%). ^1^H NMR (400 MHz, DMSO-*d*_6_) δ: 11.09 (s, 1H), 7.58 (dd, *J* = 8.6, 7.1 Hz, 1H), 7.11 (d, *J* = 8.6 Hz, 1H), 7.02 (d, *J* = 7.0 Hz, 1H), 6.61 (t, *J* = 6.1 Hz, 1H), 5.05 (dd, *J* = 12.9, 5.4 Hz, 1H), 3.41–3.33 (m, 4H), 2.96–2.81 (m, 1H), 2.62–2.52 (m, 2H), 2.06–1.96 (m, 1H), 1.66–1.59 (m, 4H). ^13^C NMR (101 MHz, DMSO-*d*_6_) δ: 172.8, 170.1, 168.8, 167.3, 146.3, 136.2, 132.2, 117.2, 110.4, 109.1, 50.3, 41.2, 39.4, 30.8, 25.9, 25.7, 22.1. ESI-MS: *m*/*z* = 371.10 ([M + H]^+^).

##### Synthesis of 4-((6-Azidohexyl)­amino)-2-(2,6-dioxopiperidin-3-yl)­isoindoline-1,3-dione (**X6**)

The title compound was prepared according to general procedure C, using 2-(2,6-dioxopiperidin-3-yl)-4-fluoroisoindoline-1,3-dione (200 mg, 0.724 mmol), 6-azidohexan-1-amine (123 mg, 0.868 mmol) and DIPEA (378 μL, 2.17 mmol). The title compound was obtained as a yellow solid (195 mg, 68%). ^1^H NMR (400 MHz, DMSO-*d*_6_) δ: 11.09 (s, 1H), 7.57 (dd, *J* = 8.6, 7.1 Hz, 1H), 7.08 (d, *J* = 8.6 Hz, 1H), 7.02 (d, *J* = 7.0 Hz, 1H), 6.53 (t, *J* = 6.0 Hz, 1H), 5.05 (dd, *J* = 12.9, 5.4 Hz, 1H), 3.33–3.25 (m, 4H), 2.95–2.77 (m, 1H), 2.64–2.51 (m, 2H), 2.06–1.96 (m, 1H), 1.68–1.49 (m, 4H), 1.36 (quin, *J* = 3.5 Hz, 4H). ^13^C NMR (101 MHz, DMSO-*d*_6_) δ: 172.8, 170.1, 168.9, 167.3, 146.4, 136.2, 132.2, 117.1, 110.3, 109.1, 50.5, 48.5, 41.7, 30.9, 28.5, 28.1, 25.8, 22.1. ESI-MS: *m*/*z* = 399.20 ([M + H]^+^).

##### Synthesis of 4-({2-[2-(2-Azidoethoxy)­ethoxy]­ethyl}­amino)-2-(2,6-dioxopiperidin-3-yl)-2,3-dihydro-1*H*-isoindole-1,3-dione (**X7**)

2-(2,6-Dioxopiperidin-3-yl)-4-fluoroisoindoline-1,3-dione (231 mg, 0.821 mmol, 1.0 equiv) and 1-(2-aminoethoxy)-2-(2-azidoethoxy)­ethane (157 mg, 0.903 mmol, 1.1 equiv) were dissolved in DMF (4 mL). *N*,*N*-Diisopropylethylamine (0.281 μL, 1.64 mmol, 2.0 equiv) was added and the reaction mixture was heated up to 90 °C for 14 h. After TLC confirmed completion of the reaction, EtOAc (50 mL) was added to the mixture and the solution was washed with brine (2 × 50 mL) and water (50 mL). The organic phase was dried over MgSO_4_ and the solvent was removed under reduced pressure. The crude product was purified by flash chromatography using acetonitrile/water as an eluent to obtain the title compound as a yellow solid (220 mg, 62%). ^1^H NMR (400 MHz, acetone-*d*_6_) δ: 9.98–9.75 (m, 1H), 7.63–7.55 (m, 1H), 7.17–7.11 (m, 1H), 7.08–7.01 (m, 1H), 6.81–6.50 (m, 1H), 5.11–5.02 (m, 1H), 3.80–3.73 (m, 2H), 3.72–3.62 (m, 5H), 3.59–3.51 (m, 2H), 3.43–3.31 (m, 2H), 3.03–2.88 (m, 1H), 2.82–2.72 (m, 4H), 2.28–2.15 (m, 1H). ^13^C NMR (101 MHz, acetone-*d*_6_) δ: 172.6, 170.2, 170.2, 168.2, 147.8, 136.8, 133.6, 117.8, 111.4, 111.1, 71.3, 71.2, 70.8, 70.2, 51.4, 49.8, 43.0, 32.0, 23.4. ESI-MS: *m*/*z* = 431.2 ([M + H]^+^).

##### Synthesis of 4-(15-Azido-4,7,10,13-tetraoxa-1-azapentadecan-1-yl)-2-(2,6-dioxopiperidin-3-yl)-2,3-dihydro-1*H*-isoindole-1,3-dione (**X8**)

2-(2,6-Dioxopiperidin-3-yl)-4-fluoroisoindoline-1,3-dione (556 mg, 1.97 mmol, 1.0 equiv) and 1-(2-aminoethoxy)-2-(2-azidoethoxy)­ethane (569 mg, 2.18 mmol, 1.1 equiv) were dissolved in DMF (10 mL). DIPEA (728 μL, 4.26 mmol, 2.2 equiv) was added and the reaction mixture was heated up to 90 °C for 14 h. After TLC confirmed completion of the reaction, EtOAc (50 mL) was added to the mixture and the solution was washed with brine (2 × 50 mL) and water (50 mL). The organic phase was dried over MgSO_4_ and the solvent was removed under reduced pressure. The crude product was purified by flash chromatography using acetonitrile/water as an eluent to obtain the title compound as a yellow solid (338 mg, 33%). ^1^H NMR (500 MHz, acetone-*d*_6_) δ: 9.86 (s, 1H), 7.63–7.56 (m, 1H), 7.14 (d, *J* = 8.5 Hz, 1H), 7.05 (dd, *J* = 7.1, 0.6 Hz, 1H), 6.62 (t, *J* = 5.7 Hz, 1H), 5.07 (dd, *J* = 12.6, 5.4 Hz, 1H), 3.75 (t, *J* = 5.4 Hz, 2H), 3.68–3.65 (m, 2H), 3.65–3.62 (m, 4H), 3.60–3.60 (m, 4H), 3.59–3.58 (m, 4H), 3.54 (q, *J* = 5.5 Hz, 2H), 3.37 (t, *J* = 5.0 Hz, 2H), 3.03–2.89 (m, 1H), 2.80–2.72 (m, 2H), 2.28–2.12 (m, 1H). ^13^C NMR (126 MHz, acetone-*d*_6_) δ: 172.6, 170.2, 170.2, 168.2, 147.8, 136.8, 133.6, 117.8, 111.4, 111.1, 71.3, 71.3, 71.2, 71.2, 70.6, 70.2, 51.4, 49.8, 43.0, 32.0, 23.4. ESI-MS: *m*/*z* = 519.2 ([M + H]^+^).

##### Synthesis of *tert*-Butyl (2-((2-(2,6-Dioxopiperidin-3-yl)-1,3-dioxoisoindolin-4-yl)­amino)-ethyl)­carbamate (**I13**)

The title compound was prepared according to general procedure C, using 2-(2,6-dioxopiperidin-3-yl)-4-fluoroisoindoline-1,3-dione (1.30 mg, 4.71 mmol), *tert*-butyl (2-aminoethyl)­carbamate (905 mg, 5.65 mmol) and DIPEA (2.46 mL, 14.1 mmol). The title compound was obtained as a yellow solid (899 mg, 46%). ^1^H NMR (400 MHz, DMSO-*d*_6_) δ: 11.08 (s, 1H), 7.58 (dd, *J* = 8.6, 7.1 Hz, 1H), 7.14 (d, *J* = 8.6 Hz, 1H), 7.06–6.95 (m, 2H), 6.71 (t, *J* = 6.2 Hz, 1H), 5.05 (dd, *J* = 12.5, 5.4 Hz, 1H), 3.41–3.34 (m, 2H), 3.12 (q, *J* = 6.1 Hz, 2H), 2.98–2.79 (m, 1H), 2.64–2.53 (m, 2H), 2.06–1.95 (m, 1H), 1.36 (s, 9H). ESI-MS: *m*/*z* = 439.10 ([M + Na]^+^).

##### Synthesis of 4-((2-Aminoethyl)­amino)-2-(2,6-dioxopiperidin-3-yl)­isoindoline-1,3-dione (**I14**)

The title compound was prepared according to general procedure D, using **I13** (169 mg, 0.406 mmol). The crude product was used without further purification. ESI-MS: *m*/*z* = 317.10 ([M + H]^+^).

##### Synthesis of 2-Azido-*N*-(2-((2-(2,6-dioxopiperidin-3-yl)-1,3-dioxoisoindolin-4-yl)­amino)-ethyl)­acetamide (**X9**)

The title compound was prepared according to general procedure A, using **I14** (128 mg, 0.406 mmol), 2-azidoacetic acid (30.4 μL, 0.406 mmol), HATU (185 mg, 0.487 mmol) and DIPEA (212 μL, 1.22 mmol). The title compound was obtained as a yellow solid (93 mg, 57%). ^1^H NMR (400 MHz, DMSO-*d*_6_) δ: 11.09 (s, 1H), 8.33 (t, *J* = 5.6 Hz, 1H), 7.59 (dd, *J* = 8.6, 7.0 Hz, 1H), 7.17 (d, *J* = 8.6 Hz, 1H), 7.04 (d, *J* = 7.0 Hz, 1H), 6.74 (t, *J* = 6.2 Hz, 1H), 5.06 (dd, *J* = 12.9, 5.4 Hz, 1H), 3.82 (s, 2H), 3.42 (q, *J* = 6.3 Hz, 2H), 3.32–3.26 (m, 2H), 2.96–2.78 (m, 1H), 2.63–2.51 (m, 2H), 2.10–1.89 (m, 1H). ^13^C NMR (101 MHz, DMSO-*d*_6_) δ: 172.8, 170.1, 168.7, 167.9, 167.3, 146.2, 136.2, 132.2, 117.1, 110.6, 109.3, 50.8, 48.5, 41.2, 38.1, 30.9, 22.1. ESI-MS: *m*/*z* = 400.20 ([M + H]^+^).

##### Synthesis of *tert*-Butyl 2-((2-(2,6-Dioxopiperidin-3-yl)-1,3-dioxoisoindolin-5-yl)­oxy)­acetate (**I15**)

2-(2,6-Dioxopiperidin-3-yl)-5-hydroxyisoindoline-1,3-dione (1.00 g, 3.65 mmol, 1 equiv) and potassium carbonate (1.26 g, 9.12 mmol, 3 equiv) were suspended in dry DMF (15 mL). The flask was cooled to 0 °C in an ice bath and *tert*-butyl bromoacetate (538 μL, 3.65 mmol, 1 equiv) was added dropwise through a dropping funnel over 30 min. The solution was warmed to ambient temperature and stirred for two h. The reaction mixture was diluted with cold water and the precipitate was filtered. The colorless solid was dried under reduced pressure to obtain the title compound (1.13 g, 80%). ^1^H NMR (400 MHz, DMSO-*d*_6_) δ: 11.11 (s, 1H), 7.85 (d, *J* = 8.2 Hz, 1H), 7.40 (s, 1H), 7.35 (dd, *J* = 8.3, 2.4 Hz, 1H), 5.11 (dd, *J* = 12.9, 5.4 Hz, 1H), 4.93 (s, 2H), 2.97–2.81 (m, 1H), 2.65–2.53 (m, 2H), 2.13–2.01 (m, 1H), 1.43 (s, 9H). ESI-MS: *m*/*z* = 389.10 ([M + H]^+^).

##### Synthesis of 2-((2-(2,6-Dioxopiperidin-3-yl)-1,3-dioxoisoindolin-5-yl)­oxy)­acetic Acid (**I16**)

**I15** (1.00 g, 3.02 mmol) was dissolved in dry DCM (5 mL). The flask was cooled to 0 °C in an ice bath and trifluoroacetic acid (TFA) in an excess was added dropwise. The solution was stirred at rt for two h. The solvent was removed under reduced pressure and the crude product used without further purification. ESI-MS: *m*/*z* = 333.05 ([M + H]^+^).

##### Synthesis of *N*-(3-Azidopropyl)-2-((2-(2,6-Dioxopiperidin-3-yl)-1,3-Dioxoisoindolin-5-yl)-oxy)­acetamide (**X10**)

The title compound was prepared according to general procedure A, using **I16** (100 mg, 0.304 mmol), 3-azidopropan-1-amine (30 mg, 0.30 mmol), HATU (150 mg, 0.395 mmol) and DIPEA (158 μL, 0.912 mmol). The title compound was obtained as a light yellow solid (121 mg, 96%). ^1^H NMR (400 MHz, DMSO-*d*_6_) δ: 11.10 (s, 1H), 8.28 (t, *J* = 5.8 Hz, 1H), 7.87 (d, *J* = 8.3 Hz, 1H), 7.44 (d, *J* = 2.3 Hz, 1H), 7.39 (dd, *J* = 8.3, 2.3 Hz, 1H), 5.12 (dd, *J* = 12.9, 5.4 Hz, 1H), 4.73 (s, 2H), 3.35 (t, *J* = 6.8 Hz, 2H), 3.20 (q, *J* = 6.5 Hz, 2H), 2.98–2.82 (m, 1H), 2.66–2.51 (m, 2H), 2.09–2.01 (m, 1H), 1.69 (quin, *J* = 6.8 Hz, 2H). ^13^C NMR (101 MHz, DMSO-*d*_6_) δ: 172.7, 169.9, 166.9, 166.7, 163.1, 133.7, 125.3, 123.5, 120.9, 109.4, 67.3, 49.0, 48.3, 35.7, 30.9, 28.3, 22.0. ESI-MS: *m*/*z* = 437.10 ([M + Na]^+^).

##### Synthesis of *N*-(2-(2-(2-Azidoethoxy)­ethoxy)­ethyl)-2-((2-(2,6-dioxopiperidin-3-yl)-1,3-dioxoisoindolin-5-yl)­oxy)­acetamide (**X11**)

The title compound was prepared according to general procedure B, using **I16** (150 mg, 0.451 mmol) and 1-(2-aminoethoxy)-2-(2-azidoethoxy)­ethane (102 mg, 0.587 mmol), 1-methyl-1*H*-imidazole (126 μL, 1.58 mmol) and TCFH (152 mg, 0.542 mmol). The title compound was obtained as a colorless solid (100 mg, 45%). ^1^H NMR (500 MHz, acetone-*d*_6_) δ: 9.91 (s, 1H), 7.85 (d, *J* = 8.2 Hz, 1H), 7.59 (s, 1H), 7.45 (d, *J* = 2.2 Hz, 1H), 7.43 (dd, *J* = 8.2, 2.3 Hz, 1H), 5.12 (dd, *J* = 12.7, 5.5 Hz, 1H), 4.75 (s, 2H), 3.67 (t, *J* = 4.8 Hz, 2H), 3.63–3.58 (m, 4H), 3.56 (t, *J* = 5.7 Hz, 2H), 3.46 (q, *J* = 5.7 Hz, 2H), 3.37 (t, *J* = 4.9 Hz, 2H), 3.08–2.90 (m, 1H), 2.81–2.73 (m, 2H), 2.29–2.17 (m, 1H). ^13^C NMR (126 MHz, acetone-*d*_6_) δ: 172.6, 170.0, 167.6, 167.6, 167.5, 164.0, 135.3, 126.0, 125.4, 121.5, 110.2, 71.1, 71.0, 70.7, 70.2, 68.5, 51.3, 50.3, 39.5, 31.9, 23.3. ESI-MS: *m*/*z* = 489.1 ([M + H]^+^).

##### Synthesis of *N*-(2-(2-(2-(2-Azidoethoxy)­ethoxy)­ethoxy)­ethyl)-2-((2-(2,6-dioxopiperidin-3-yl)-1,3-dioxoisoindolin-5-yl)­oxy)­acetamide (**X12**)

The title compound was prepared according to general procedure B, using **I16** (180 mg, 0.542 mmol) and 1-[2-(2-aminoethoxy)­ethoxy]-2-(2-azidoethoxy)­ethane (130 mg, 0.596 mmol), 1-methyl-1*H*-imidazole (151 μL, 1.90 mmol) and TCFH (182 mg, 0.650 mmol). The title compound was obtained as a colorless solid (110 mg, 38%). ^1^H NMR (400 MHz, acetone-*d*_6_) δ: 9.90 (s, 1H), 7.85 (dd, *J* = 8.1, 0.7 Hz, 1H), 7.60 (s, 1H), 7.45 (dd, *J* = 2.4, 0.6 Hz, 1H), 7.43 (dd, *J* = 8.1, 2.4 Hz, 1H), 5.12 (dd, *J* = 12.6, 5.5 Hz, 1H), 4.75 (s, 2H), 3.68–3.64 (m, 2H), 3.62–3.60 (m, 4H), 3.57 (q, *J* = 1.2 Hz, 4H), 3.56–3.53 (m, 2H), 3.45 (q, *J* = 5.7 Hz, 2H), 3.38 (t, *J* = 5.0 Hz, 2H), 3.04–2.91 (m, 1H), 2.78–2.70 (m, 2H), 2.28–2.17 (m, 1H). ^13^C NMR (101 MHz, acetone-*d*_6_) δ: 171.7, 169.1, 166.7, 166.7, 166.6, 163.1, 134.4, 125.1, 124.5, 120.6, 109.4, 70.3, 70.3, 70.0, 69.8, 69.2, 67.7, 50.4, 49.4, 38.9, 31.1, 22.4. ESI-MS: *m*/*z* = 533.2 ([M + H]^+^).

##### Synthesis of 5-((3-Azidopropyl)­amino)-2-(2,6-dioxopiperidin-3-yl)­isoindoline-1,3-dione (**X13**)

The title compound was prepared according to general procedure C, using 2-(2,6-dioxopiperidin-3-yl)-5-fluoroisoindoline-1,3-dione (200 mg, 0.724 mmol), 3-azidopropan-1-amine (87 mg, 0.87 mmol) and DIPEA (378 μL, 2.17 mmol). The title compound was obtained as a yellow solid (60 mg, 23%). ^1^H NMR (400 MHz, DMSO-*d*_6_) δ: 11.05 (s, 1H), 7.57 (d, *J* = 8.3 Hz, 1H), 7.15 (t, *J* = 5.5 Hz, 1H), 6.96 (d, *J* = 2.1 Hz, 1H), 6.86 (dd, *J* = 8.4, 2.2 Hz, 1H), 5.03 (dd, *J* = 12.9, 5.4 Hz, 1H), 3.46 (t, *J* = 6.7 Hz, 2H), 3.24 (q, *J* = 6.5 Hz, 2H), 2.96–2.80 (m, 1H), 2.65–2.52 (m, 2H), 2.08–1.95 (m, 1H), 1.82 (quin, *J* = 6.8 Hz, 2H). ^13^C NMR (101 MHz, DMSO-*d*_6_) δ: 172.8, 170.1, 167.6, 167.1, 154.2, 134.2, 125.1, 116.2, 48.6, 48.3, 39.6, 30.9, 27.5, 22.2. ESI-MS: *m*/*z* = 357.20 ([M + H]^+^).

##### Synthesis of 5-((4-Azidobutyl)­amino)-2-(2,6-dioxopiperidin-3-yl)­isoindoline-1,3-dione (**X14**)

The title compound was prepared according to general procedure C, using 2-(2,6-dioxopiperidin-3-yl)-5-fluoroisoindoline-1,3-dione (200 mg, 0.724 mmol), 4-azidobutan-1-amine (99 mg, 0.87 mmol) and DIPEA (378 μL, 2.17 mmol). The title compound was obtained as a yellow solid (93 mg, 35%). ^1^H NMR (400 MHz, DMSO-*d*_6_) δ: 11.05 (s, 1H), 7.56 (d, *J* = 8.4 Hz, 1H), 7.13 (t, *J* = 5.5 Hz, 1H), 6.96 (d, *J* = 2.1 Hz, 1H), 6.85 (dd, *J* = 8.4, 2.1 Hz, 1H), 5.03 (dd, *J* = 12.8, 5.5 Hz, 1H), 3.44–3.34 (m, 2H), 3.19 (q, *J* = 6.1 Hz, 2H), 2.95–2.78 (m, 1H), 2.64–2.50 (m, 2H), 2.07–1.89 (m, 1H), 1.73–1.49 (m, 4H). ^13^C NMR (101 MHz, DMSO-*d*_6_) δ: 172.8, 170.1, 167.7, 167.1, 154.4, 134.2, 125.1, 115.9, 50.3, 48.6, 41.9, 30.9, 25.9, 25.4, 22.2. ESI-MS: *m*/*z* = 371.20 ([M + H]^+^).

##### Synthesis of 5-((6-Azidohexyl)­amino)-2-(2,6-dioxopiperidin-3-yl)­isoindoline-1,3-dione (**X15**)

The title compound was prepared according to general procedure C, using 2-(2,6-dioxopiperidin-3-yl)-5-fluoroisoindoline-1,3-dione (200 mg, 0.724 mmol), 6-azidohexan-1-amine (124 mg, 0.867 mmol) and DIPEA (378 μL, 2.17 mmol). The title compound was obtained as a yellow solid (107 mg, 37%). ^1^H NMR (400 MHz, DMSO-*d*_6_) δ: 11.05 (s, 1H), 7.56 (d, *J* = 8.4 Hz, 1H), 7.09 (t, *J* = 5.4 Hz, 1H), 6.94 (d, *J* = 2.1 Hz, 1H), 6.84 (dd, *J* = 8.4, 2.1 Hz, 1H), 5.03 (dd, *J* = 12.9, 5.4 Hz, 1H), 3.44–3.24 (m, 2H), 3.15 (q, *J* = 6.5 Hz, 2H), 2.93–2.80 (m, 1H), 2.62–2.50 (m, 2H), 2.05–1.85 (m, 1H), 1.65–1.46 (m, 4H), 1.43–1.33 (m, 4H). ^13^C NMR (101 MHz, DMSO-*d*_6_) δ: 172.8, 170.1, 167.7, 167.1, 154.4, 134.2, 125.1, 115.8, 50.8, 48.6, 42.3, 30.9, 28.2, 28.0, 26.0, 25.9, 22.2. ESI-MS: *m*/*z* = 421.20 ([M + H]^+^).

##### Synthesis of *tert*-Butyl (2-((2-(2,6-Dioxopiperidin-3-yl)-1,3-dioxoisoindolin-5-yl)­amino)-ethyl)­carbamate (**I17**)

The title compound was prepared according to general procedure C, using 2-(2,6-dioxopiperidin-3-yl)-5-fluoroisoindoline-1,3-dione (2.00 g, 7.24 mmol), *tert*-butyl (2-aminoethyl)­carbamate (1.39 g, 8.69 mmol) and DIPEA (3.78 mL, 21.7 mmol). The title compound was obtained as a yellow solid (293 mg, 10%). ^1^H NMR (400 MHz, DMSO-*d*_6_) δ: 11.05 (s, 1H), 7.56 (d, *J* = 8.4 Hz, 1H), 7.13 (t, *J* = 5.9 Hz, 1H), 6.99–6.96 (m, 1H), 6.91 (t, *J* = 4.7 Hz, 1H), 6.86 (dd, *J* = 8.4, 2.2 Hz, 1H), 5.03 (dd, *J* = 12.9, 5.4 Hz, 1H), 3.22 (quin, *J* = 6.6 Hz, 2H), 3.15–3.09 (m, 2H), 2.96–2.81 (m, 1H), 2.60–2.51 (m, 2H), 2.04–1.94 (m, 1H), 1.37 (s, 9H). ESI-MS: *m*/*z* = 439.10 ([M + Na]^+^).

##### Synthesis of 5-((2-Aminoethyl)­amino)-2-(2,6-dioxopiperidin-3-yl)­isoindoline-1,3-dione (**I18**)

The title compound was prepared according to general procedure D, using **I17** (293 mg, 0.704 mmol). The crude product was used without further purification. ESI-MS: *m*/*z* = 317.15 ([M + H]^+^).

##### Synthesis of 2-Azido-*N*-(2-((2-(2,6-Dioxopiperidin-3-yl)-1,3-dioxoisoindolin-5-yl)­amino)-ethyl)­acetamide (**X16**)

The title compound was prepared according to general procedure A, using **I18** (128 mg, 0.405 mmol), 2-azidoacetic acid (41 mg, 0.41 mmol), HATU (185 mg, 487 mmol) and DIPEA (282 μL, 1.62 mmol). The title compound was obtained as a yellow solid (70 mg, 43%). ^1^H NMR (400 MHz, DMSO-*d*_6_) δ: 11.06 (s, 1H), 8.25 (t, *J* = 5.1 Hz, 1H), 7.58 (d, *J* = 8.4 Hz, 1H), 7.17 (t, *J* = 5.2 Hz, 1H), 7.00 (d, *J* = 2.1 Hz, 1H), 6.88 (dd, *J* = 8.4, 2.2 Hz, 1H), 5.03 (dd, *J* = 12.9, 5.4 Hz, 1H), 3.84 (s, 2H), 3.31–3.24 (m, 4H), 2.93–2.83 (m, 1H), 2.63–2.51 (m, 2H), 2.03–1.96 (m, 1H). ^13^C NMR (101 MHz, DMSO-*d*_6_) δ: 172.8, 170.1, 167.6, 167.1, 154.2, 134.2, 125.2, 116.3, 50.8, 48.6, 41.6, 37.7, 30.9, 22.2. ESI-MS: *m*/*z* = 400.15 ([M + H]^+^).

##### Synthesis of *tert*-Butyl 4-(1-(2-(2,6-Dioxopiperidin-3-yl)-1,3-dioxoisoindolin-5-yl)-piperidin-4-yl)­piperazine-1-carboxylate (**I19**)

The title compound was prepared according to general procedure C, using 2-(2,6-dioxopiperidin-3-yl)-5-fluoroisoindoline-1,3-dione (500 mg, 1.81 mmol), *tert*-butyl 4-(piperidin-4-yl)­piperazine-1-carboxylate (731 mg, 2.72 mmol) and DIPEA (1.26 mL, 7.24 mmol). The title compound was obtained as a yellow solid (551 mg, 58%). ^1^H NMR (400 MHz, DMSO-*d*_6_) δ: 11.07 (s, 1H), 7.65 (d, *J* = 8.5 Hz, 1H), 7.31 (d, *J* = 2.3 Hz, 1H), 7.23 (dd, *J* = 8.7, 2.4 Hz, 1H), 5.06 (dd, *J* = 12.9, 5.4 Hz, 1H), 4.06 (d, *J* = 13.3 Hz, 2H), 3.28 (t, *J* = 5.0 Hz, 4H), 2.94 (t, 2H), 2.90–2.82 (m, 1H), 2.65–2.53 (m, 2H), 2.49–2.47 (m, 1H), 2.42 (t, *J* = 5.1 Hz, 4H), 2.04–1.97 (m, 1H), 1.82 (d, *J* = 10.8 Hz, 2H), 1.53–1.41 (m, 2H), 1.38 (s, 9H). ESI-MS: *m*/*z* = 526.20 ([M + H]^+^).

##### Synthesis of 2-(2,6-Dioxopiperidin-3-yl)-5-(4-(piperazin-1-yl)­piperidin-1-yl)­isoindoline-1,3-dione (**I20**)

The title compound was prepared according to general procedure D, using **I19** (500 mg, 0.951 mmol). The crude product was used without further purification. ESI-MS: *m*/*z* = 426.15 ([M + H]^+^).

##### Synthesis of 5-(4-(4-(2-Azidoacetyl)­piperazin-1-yl)­piperidin-1-yl)-2-(2,6-dioxopiperidin-3-yl)­isoindoline-1,3-dione (**X17**)

The title compound was prepared according to general procedure A, using **I20** (134 mg, 0.315 mmol), 2-azidoacetic acid (32 mg, 0.32 mmol), HATU (156 mg, 0.409 mmol) and DIPEA (165 μL, 0.945 mmol). The title compound was obtained as a yellow solid (102 mg, 64%). ^1^H NMR (400 MHz, DMSO-*d*_6_) δ: 11.08 (s, 1H), 7.65 (d, *J* = 8.5 Hz, 1H), 7.32 (d, *J* = 2.3 Hz, 1H), 7.24 (dd, *J* = 8.7, 2.3 Hz, 1H), 5.06 (dd, *J* = 13.0, 5.4 Hz, 1H), 4.12 (s, 2H), 4.07 (d, *J* = 13.4 Hz, 2H), 3.44 (t, *J* = 4.9 Hz, 2H), 3.28 (t, *J* = 5.0 Hz, 2H), 2.96 (t, *J* = 12.3 Hz, 2H), 2.91–2.81 (m, 1H), 2.64–2.52 (m, 3H), 2.48–2.46 (m, 4H), 2.04–1.99 (m, 1H), 1.81 (d, *J* = 10.4 Hz, 2H), 1.51–1.40 (m, 2H). ^13^C NMR (101 MHz, DMSO-*d*_6_) δ: 172.8, 170.1, 167.6, 167.0, 166.9, 165.7, 154.7, 134.0, 125.0, 117.6, 107.8, 60.5, 49.6, 48.7, 48.6, 48.3, 46.6, 44.4, 41.8, 30.9, 27.0, 22.1. ESI-MS: *m*/*z* = 509.20 ([M + H]^+^).

##### Synthesis of 5-(4-(4-(4-Azidobutanoyl)­piperazin-1-yl)­piperidin-1-yl)-2-(2,6-dioxopiperidin-3-yl)­isoindoline-1,3-dione (**X18**)

The title compound was prepared according to general procedure A, using **I20** (134 mg, 0.315 mmol), 4-azidobutanoic acid (37 mg, 0.32 mmol), HATU (156 mg, 0.409 mmol) and DIPEA (165 μL, 0.945 mmol). The title compound was obtained as a yellow solid (82 mg, 50%). ^1^H NMR (400 MHz, DMSO-*d*_6_) δ: 11.08 (s, 1H), 7.65 (d, *J* = 8.5 Hz, 1H), 7.32 (d, *J* = 2.3 Hz, 1H), 7.24 (dd, *J* = 8.7, 2.3 Hz, 1H), 5.06 (dd, *J* = 12.9, 5.4 Hz, 1H), 4.07 (d, *J* = 12.9 Hz, 2H), 3.45–3.38 (m, 4H), 3.36 (s, 1H), 3.33–3.32 (m, 1H), 2.96 (t, *J* = 12.6 Hz, 2H), 2.90–2.81 (m, 1H), 2.63–2.52 (m, 3H), 2.49–2.45 (m, 2H), 2.42 (t, *J* = 5.0 Hz, 2H), 2.36 (t, *J* = 7.3 Hz, 2H), 2.04–1.97 (m, 1H), 1.82 (d, *J* = 12.5 Hz, 2H), 1.73 (quin, *J* = 7.1 Hz, 2H), 1.55–1.38 (m, 2H). ^13^C NMR (101 MHz, DMSO-*d*_6_) δ: 172.8, 170.1, 169.5, 167.6, 166.9, 154.7, 134.0, 125.0, 117.6, 117.6, 107.8, 60.5, 50.2, 48.9, 48.7, 48.5, 46.6, 45.1, 41.4, 30.9, 29.0, 27.0, 24.0, 22.1. ESI-MS: *m*/*z* = 537.20 ([M + H]^+^).

##### Synthesis of 1-(3-Hydroxy-2-methylphenyl)­dihydropyrimidine-2,4­(1*H*,3*H*)-dione (**I21**)

3-amino-2-methylphenol (4.00 g, 32.5 mmol) and acrylic acid (3.34 mL, 48.7 mmol) were dissolved in dry toluene (20 mL) and stirred at 110 °C for 4 h. The solvent was removed under reduced pressure and the crude reaction mixture diluted with acetic acid (25 mL). Urea (5.85 g, 97.4 mmol) was added and stirred at 100 °C for 18 h. The reaction mixture was cooled in an ice bath (0 °C) and diluted with water, filtered and the precipitate was washed with water (20 mL) and *n*-hexane (20 mL). The precipitate was dried under reduced pressure to give the title compound as a colorless solid (6.61 g, 92%). ^1^H NMR (400 MHz, DMSO-*d*_6_) δ: 10.27 (s, 1H), 9.48 (s, 1H), 7.01 (t, *J* = 7.8 Hz, 1H), 6.77 (dd, *J* = 8.1, 1.2 Hz, 1H), 6.70 (dd, *J* = 7.9, 1.2 Hz, 1H), 3.80–3.63 (m, 1H), 3.53–3.40 (m, 1H), 2.87–2.58 (m, 2H), 1.96 (s, 3H). ESI-MS: *m*/*z* = 221.05 ([M + H]^+^).

##### Synthesis of *tert*-Butyl 2-(3-(2,4-Dioxotetrahydropyrimidin-1­(2*H*)-yl)-2-methylphenoxy)-acetate (**I22**)

**I21** (2.00 g, 9.08 mmol) and potassium carbonate (3.77 g, 27.2 mmol) were dissolved in dry DMF (20 mL). *Tert*-butyl bromoacetate (1.41 mL, 9.54 mmol) was added dropwise and the reaction mixture was stirred at rt for 2 h. The reaction mixture was diluted with water, filtered and the precipitate was washed with water (10 mL) and *n*-hexane (10 mL). The precipitate was dried under reduced pressure to give the title compound (2.49 g, 82%). ^1^H NMR (400 MHz, DMSO-*d*_6_) δ: 10.31 (s, 1H), 7.17 (t, *J* = 8.2 Hz, 1H), 6.90 (d, *J* = 7.9 Hz, 1H), 6.80 (d, *J* = 8.3 Hz, 1H), 4.69 (s, 2H), 3.84–3.66 (m, 1H), 3.54–3.40 (m, 1H), 2.86–2.60 (m, 2H), 2.05 (s, 3H), 1.43 (s, 9H). ESI-MS: *m*/*z* = 357.05 ([M + Na]^+^).

##### Synthesis of 2-(3-(2,4-Dioxotetrahydropyrimidin-1­(2*H*)-yl)-2-methylphenoxy)­acetic Acid (**I23**)

**I22** (1.00 g, 2.99 mmol) was dissolved in dry DCM (10 mL). The flask was cooled to 0 °C in an ice bath and trifluoroacetic acid (TFA) in an excess was added dropwise. The solution was stirred at rt for two h. The solvent was removed under reduced pressure and the crude product used without further purification. ESI-MS: *m*/*z* = 279.95 ([M + H]^+^).

##### Synthesis of *N*-(2-(2-(2-Azidoethoxy)­ethoxy)­ethyl)-2-(3-(2,4-dioxotetrahydropyrimidin-1­(2*H*)-yl)-2-methylphenoxy)­acetamide (**X19**)

The title compound was prepared according to general procedure A, using **I23** (125 mg, 0.449 mmol), 2-(2-(2-azidoethoxy)­ethoxy)­ethan-1-amine (78 mg, 0.45 mmol), HATU (256 mg, 0.674 mmol) and DIPEA (234 μL, 1.35 mmol). The title compound was obtained as a colorless solid (122 mg, 62%). ^1^H NMR (400 MHz, DMSO-*d*_6_) δ: 10.33 (s, 1H), 7.95 (t, *J* = 5.7 Hz, 1H), 7.17 (t, *J* = 8.1 Hz, 1H), 6.91 (d, *J* = 7.6 Hz, 1H), 6.84 (d, *J* = 8.1 Hz, 1H), 4.51 (s, 2H), 3.80–3.67 (m, 1H), 3.61–3.57 (m, 2H), 3.57–3.51 (m, 6H), 3.51–3.45 (m, 3H), 3.42–3.36 (m, 2H), 2.82–2.63 (m, 2H), 2.08 (s, 3H). ^13^C NMR (101 MHz, DMSO-*d*_6_) δ: 170.7, 167.7, 156.3, 151.7, 141.7, 126.6, 124.5, 119.9, 110.8, 69.6, 69.6, 69.7, 68.7, 67.5, 49.9, 44.6, 38.2, 31.0, 10.8. ESI-MS: *m*/*z* = 435.20 ([M + H]^+^).

##### Synthesis of *N*-(20-Azido-3,6,9,12,15,18-hexaoxaicosyl)-2-(3-(2,4-dioxotetrahydro-pyrimidin-1­(2*H*)-yl)-2-methylphenoxy)­acetamide (**X20**)

The title compound was prepared according to general procedure A, using **I23** (125 mg, 0.449 mmol), 20-azido-3,6,9,12,15,18-hexaoxaicosan-1-amine (157 mg, 0.449 mmol), HATU (256 mg, 0.674 mmol) and DIPEA (234 μL, 1.35 mmol). The title compound was obtained as a colorless solid (163 mg, 59%). ^1^H NMR (400 MHz, DMSO-*d*_6_) δ: 10.33 (s, 1H), 7.96 (t, *J* = 5.7 Hz, 1H), 7.18 (t, *J* = 8.1 Hz, 1H), 6.91 (d, *J* = 7.9 Hz, 1H), 6.84 (d, *J* = 8.3 Hz, 1H), 4.52 (s, 2H), 3.81–3.70 (m, 1H), 3.59 (t, *J* = 4.9 Hz, 2H), 3.56–3.44 (m, 25H), 3.38 (t, *J* = 4.9 Hz, 2H), 2.84–2.61 (m, 2H), 2.08 (s, 3H). ^13^C NMR (101 MHz, DMSO-*d*_6_) δ: 170.7, 167.6, 156.3, 151.7, 141.7, 126.6, 124.5, 119.9, 110.8, 69.8, 69.8, 69.7, 69.6, 69.2, 68.8, 67.5, 50.0, 44.6, 39.3, 38.2, 31.0, 10.8. ESI-MS: *m*/*z* = 633.25 ([M + Na]^+^).

##### Synthesis of *tert*-Butyl 4-(2-(3-(2,4-Dioxotetrahydropyrimidin-1­(2*H*)-yl)-2-methyl-phenoxy)­acetyl)­piperazine-1-carboxylate (**I24**)

The title compound was prepared according to general procedure A, using *I* **23** (487 mg, 1.75 mmol), *tert*-butyl piperazine-1-carboxylate (326 mg, 1.75 mmol), HATU (865 mg, 2.27 mmol) and DIPEA (915 μL, 3.42 mmol). The title compound was obtained as a colorless solid (721 mg, 92%). ^1^H NMR (400 MHz, DMSO-*d*_6_) δ: 10.32 (s, 1H), 7.16 (t, *J* = 8.1 Hz, 1H), 6.88 (d, *J* = 7.9 Hz, 1H), 6.85 (d, *J* = 8.3 Hz, 1H), 4.87 (s, 2H), 3.83–3.68 (m, 1H), 3.50 (t, *J* = 6.1 Hz, 1H), 3.48–3.44 (m, 4H), 3.38 (s, 2H), 3.30 (s, 2H), 2.84–2.61 (m, 2H), 2.05 (s, 3H), 1.41 (s, 9H). ESI-MS: *m*/*z* = 469.20 ([M + Na]^+^).

##### Synthesis of 1-(2-Methyl-3-(2-oxo-2-(piperazin-1-yl)­ethoxy)­phenyl)­dihydropyrimidine-2,4­(1H,3H)-dione (**I25**)

The title compound was prepared according to general procedure D, using **I24** (710 mg, 1.59 mmol). The crude product was used without further purification. ESI-MS: *m*/*z* = 347.15 ([M + H]^+^).

##### Synthesis of 1-(3-(2-(4-(2-Azidoacetyl)­piperazin-1-yl)-2-oxoethoxy)-2-methylphenyl)-dihydropyrimidine-2,4­(1*H*,3*H*)-dione (**X21**)

The title compound was prepared according to general procedure A, using **I25** (138 mg, 0.398 mmol), 2-azidoacetic acid (40 mg, 0.39 mmol), HATU (197 mg, 0.518 mmol) and DIPEA (207 μL, 1.19 mmol). The title compound was obtained as a colorless solid (132 mg, 77%). ^1^H NMR (400 MHz, DMSO-*d*_6_) δ: 10.32 (s, 1H), 7.17 (t, *J* = 8.1 Hz, 1H), 6.87 (t, *J* = 8.4 Hz, 2H), 4.90 (s, 2H), 4.18 (s, 2H), 3.82–3.66 (m, 1H), 3.55–3.47 (m, 7H), 3.40 (s, 2H), 2.86–2.61 (m, 2H), 2.05 (s, 3H). ^13^C NMR (101 MHz, DMSO-*d*_6_) δ: 170.7, 166.2, 166.1, 156.5, 151.7, 141.7, 126.5, 124.1, 119.5, 110.7, 66.4, 49.7, 44.7, 44.0, 43.7, 41.0, 31.0, 10.7. ESI-MS: *m*/*z* = 430.15 ([M + H]^+^).

##### Synthesis of 1-(3-(2-(4-(3-Azidopropanoyl)­piperazin-1-yl)-2-oxoethoxy)-2-methylphenyl)-dihydropyrimidine-2,4­(1*H*,3*H*)-dione (**X22**)

The title compound was prepared according to general procedure A, using **I25** (138 mg, 0.398 mmol), 3-azidopropanoic acid (46 mg, 0.39 mmol), HATU (197 mg, 0.518 mmol) and DIPEA (207 μL, 1.19 mmol). The title compound was obtained as a colorless solid (118 mg, 67%). ^1^H NMR (400 MHz, DMSO-*d*_6_) δ: 10.32 (s, 1H), 7.17 (t, *J* = 8.1 Hz, 1H), 6.88 (t, *J* = 8.3 Hz, 2H), 4.90 (s, 2H), 3.84–3.67 (m, 1H), 3.57–3.43 (m, 11H), 2.85–2.73 (m, 1H), 2.71–2.59 (m, 3H), 2.05 (s, 3H). ^13^C NMR (101 MHz, DMSO-*d*_6_) δ: 170.7, 168.7, 166.1, 156.7, 151.7, 141.7, 126.5, 124.1, 119.5, 110.7, 66.4, 46.6, 44.6, 44.3, 43.9, 41.1, 40.8, 31.7, 31.0, 10.7. ESI-MS: *m*/*z* = 444.25 ([M + H]^+^).

##### Synthesis of 1-(3-(2-(4-(4-Azidobutanoyl)­piperazin-1-yl)-2-oxoethoxy)-2-methylphenyl)-odihydropyrimidine-2,4­(1*H*,3*H*)-dione (**X23**)

The title compound was prepared according to general procedure A, using **I25** (138 mg, 0.398 mmol), 4-azidobutanoic acid (51 mg, 0.39 mmol), HATU (197 mg, 0.518 mmol) and DIPEA (207 μL, 1.19 mmol). The title compound was obtained as a colorless solid (130 mg, 71%). ^1^H NMR (400 MHz, DMSO-*d*_6_) δ: 10.32 (s, 1H), 7.17 (t, *J* = 8.1 Hz, 1H), 6.87 (t, *J* = 8.8 Hz, 2H), 4.89 (s, 2H), 3.81–3.70 (m, 1H), 3.66–3.43 (m, 9H), 3.36 (t, *J* = 7.1 Hz, 2H), 2.87–2.58 (m, 2H), 2.42 (t, *J* = 7.3 Hz, 2H), 2.05 (s, 3H), 1.77 (quin, *J* = 7.1 Hz, 2H). ^13^C NMR (101 MHz, DMSO-*d*_6_) δ: 170.7, 170.1, 166.1, 156.5, 151.7, 141.7, 126.5, 124.1, 119.5, 110.7, 66.4, 50.2, 48.6, 44.7, 44.7, 43.9, 41.3, 41.1, 31.0, 29.1, 24.0, 10.7. ESI-MS: *m*/*z* = 458.20 ([M + H]^+^).

##### Synthesis of 1-(3-(2-(4-(4-Azidobenzoyl)­piperazin-1-yl)-2-oxoethoxy)-2-methylphenyl)-dihydropyrimidine-2,4­(1*H*,3*H*)-dione (**X24**)

The title compound was prepared according to general procedure A, using **I25** (138 mg, 0.398 mmol), 4-azidobenzoic acid (65 mg, 0.39 mmol), HATU (197 mg, 0.518 mmol) and DIPEA (207 μL, 1.19 mmol). The title compound was obtained as a colorless solid (131 mg, 67%). ^1^H NMR (400 MHz, DMSO-*d*_6_) δ: 10.32 (s, 1H), 7.49 (d, *J* = 8.0 Hz, 2H), 7.22–7.12 (m, 3H), 6.87 (dd, *J* = 10.8, 8.0 Hz, 2H), 4.90 (s, 2H), 3.81–3.69 (m, 1H), 3.60–3.37 (m, 9H), 2.85–2.58 (m, 2H), 2.05 (s, 3H). ^13^C NMR (101 MHz, DMSO-*d*_6_) δ: 170.7, 168.5, 166.1, 156.5, 151.7, 141.6, 140.8, 132.1, 129.1, 126.4, 124.1, 119.5, 119.1, 110.6, 66.4, 44.6, 31.0, 10.7. ESI-MS: *m*/*z* = 492.15 ([M + H]^+^).

##### Synthesis of 1-(3-Iodo-2-methylphenyl)­dihydropyrimidine-2,4­(1*H*,3*H*)-dione (**I26**)

3-iodo-2-methylaniline (10.0 g, 42.9 mmol) and acrylic acid (4.42 mL, 64.4 mmol) were dissolved in dry toluene (30 mL) and stirred at 110 °C for 4 h. The solvent was removed under reduced pressure and the crude reaction mixture diluted with acetic acid (35 mL). Urea (7.73 g, 0.129 mol) was added and stirred at 100 °C for 18 h. The reaction mixture was cooled in an ice bath (0 °C) and diluted with water, filtered and the precipitate was washed with water (20 mL) and *n*-hexane (20 mL). The precipitate was dried under reduced pressure to give the title compound as a light-brown solid (11.9 g, 84%). ^1^H NMR (400 MHz, DMSO-*d*_6_) δ: 10.39 (s, 1H), 7.82 (dd, *J* = 7.9, 1.2 Hz, 1H), 7.33 (dd, *J* = 7.9, 1.2 Hz, 1H), 7.03 (t, *J* = 8.0 Hz, 1H), 3.84–3.66 (m, 1H), 3.57–3.48 (m, 1H), 2.85–2.61 (m, 2H), 2.28 (s, 3H). ESI-MS: *m*/*z* = 331.85 ([M + H]^+^).

##### Synthesis of 1-(3-Azido-2-methylphenyl)­dihydropyrimidine-2,4­(1*H*,3*H*)-dione (**X25**)

**I26** (200 mg, 0.606 mmol), sodium azide (79 mg, 1.2 mmol), copper iodide (23 mg, 0.12 mmol), l-proline (28 mg, 0.24 mmol) and potassium carbonate (236 mg, 1.82 mmol) were dissolved in dry DMSO (2 mL) and stirred at 80 °C for 18 h. The solvent was removed under reduced pressure and the crude product was purified by flash chromatography using acetonitrile/water as an eluent. The title compound was obtained as a colorless solid (48 mg, 32%). ^1^H NMR (400 MHz, DMSO-*d*_6_) δ: 10.38 (s, 1H), 7.34 (t, *J* = 7.9 Hz, 1H), 7.24 (dd, *J* = 8.1, 1.2 Hz, 1H), 7.13 (dd, *J* = 7.8, 1.2 Hz, 1H), 3.89–3.69 (m, 1H), 3.54–3.41 (m, 1H), 2.87–2.73 (m, 1H), 2.71–2.60 (m, 1H), 2.03 (s, 3H). ^13^C NMR (101 MHz, DMSO-*d*_6_) δ: 170.7, 151.7, 142.3, 138.7, 127.5, 127.3, 123.6, 117.4, 44.5, 31.0, 12.0. ESI-MS: *m*/*z* = 246.10 ([M + H]^+^).

##### Synthesis of *tert*-Butyl (*E*)-4-(3-(2,4-Dioxotetrahydropyrimidin-1­(2*H*)-yl)-2-methylstyryl)-piperidine-1-carboxylate (**I27**)

**I26** (500 mg, 1.52 mmol), *tert*-butyl 4-vinylpiperidine-1-carboxylate (640 mg, 3.03 mmol), potassium acetate (297 mg, 3.03 mmol) and palladium­(II) acetate (17 mg, 0.076 mmol) were dissolved in dry DMF (5 mL) and stirred at 100 °C for 18 h. The solvent was removed under reduced pressure and the crude product was purified by flash chromatography using acetonitrile/water as an eluent. The title compound was obtained as a colorless solid (590 mg, 94%). ^1^H NMR (400 MHz, DMSO-*d*_6_) δ: 10.32 (s, 1H), 7.39 (d, *J* = 7.4 Hz, 1H), 7.24–7.08 (m, 3H), 6.63 (d, *J* = 15.8 Hz, 1H), 6.09 (dd, *J* = 15.8, 6.8 Hz, 1H), 3.97 (d, *J* = 12.9 Hz, 2H), 3.81–3.67 (m, 1H), 3.55–3.44 (m, 1H), 2.86–2.61 (m, 4H), 2.41–2.30 (m, 1H), 2.12 (s, 3H), 2.11–2.04 (m, 1H), 1.74 (d, *J* = 12.5 Hz, 2H), 1.41 (s, 9H). ESI-MS: *m*/*z* = 436.25 ([M + Na]^+^).

##### Synthesis of (*E*)-1-(2-Methyl-3-(2-(piperidin-4-yl)­vinyl)­phenyl)­dihydropyrimidine-2,4­(1*H*,3*H*)-dione (**I28**)

The title compound was prepared according to general procedure D, using **I27** (310 mg, 0.939 mmol). The crude product was used without further purification. ESI-MS: *m*/*z* = 314.20 ([M + H]^+^).

##### Synthesis of (*E*)-1-(3-(2-(1-(2-Azidoacetyl)­piperidin-4-yl)­vinyl)-2-methylphenyl)­dihydro-pyrimidine-2,4­(1*H*,3*H*)-Dione (**X26**)

The title compound was prepared according to general procedure A, using **I28** (118 mg, 0.377 mmol), 2-azidoacetic acid (38 mg, 0.38 mmol), HATU (186 mg, 0.489 mmol) and DIPEA (197 μL, 1.13 mmol). The title compound was obtained as a colorless solid (106 mg, 71%). ^1^H NMR (400 MHz, DMSO-*d*_6_) δ: 10.33 (s, 1H), 7.39 (d, *J* = 7.4 Hz, 1H), 7.28–7.06 (m, 3H), 6.64 (d, *J* = 15.8 Hz, 1H), 6.10 (dd, *J* = 15.9, 6.7 Hz, 1H), 4.35 (q, *J* = 16.2 Hz, 1H), 4.25–4.01 (m, 3H), 3.85–3.57 (m, 2H), 3.58–3.37 (m, 1H), 3.17–2.89 (m, 1H), 2.90–2.54 (m, 4H), 2.47–2.36 (m, 1H), 1.89–1.54 (m, 2H), 1.55–1.23 (m, 2H). ^13^C NMR (101 MHz, DMSO-*d*_6_) δ: 170.7, 165.5, 151.9, 142.4, 141.2, 137.7, 136.7, 132.7, 126.4, 125.9, 124.7, 113.2, 49.7, 44.7, 43.9, 41.7, 40.1, 39.5, 31.1, 31.0, 13.7. ESI-MS: *m*/*z* = 397.15 ([M + H]^+^).

##### Synthesis of (*E*)-1-(3-(2-(1-(4-Azidobenzoyl)­piperidin-4-yl)­vinyl)-2-methylphenyl)-dihydropyrimidine-2,4­(1*H*,3*H*)-dione (**X27**)

The title compound was prepared according to general procedure A, using **I28** (118 mg, 0.377 mmol), 4-azidobenzoic acid (61 mg, 0.38 mmol), HATU (186 mg, 0.489 mmol) and DIPEA (197 μL, 1.13 mmol). The title compound was obtained as a colorless solid (88 mg, 51%). ^1^H NMR (400 MHz, DMSO-*d*_6_) δ: 10.34 (s, 1H), 7.41 (dd, *J* = 7.7, 1.6 Hz, 1H), 7.28–7.10 (m, 3H), 6.66 (d, *J* = 15.8 Hz, 1H), 6.12 (dd, *J* = 15.8, 6.7 Hz, 1H), 4.49–4.32 (m, 1H), 4.23–4.04 (m, 3H), 3.84–3.66 (m, 2H), 3.56–3.42 (m, 1H), 3.06 (t, *J* = 12.5 Hz, 1H), 2.84–2.64 (m, 3H), 2.14 (s, 3H), 1.87–1.60 (m, 3H), 1.49–1.20 (m, 2H). ^13^C NMR (101 MHz, DMSO-*d*_6_) δ: 170.7, 165.5, 151.8, 142.4, 141.2, 137.7, 136.7, 132.7, 126.4, 126.0, 125.9, 124.7, 113.2, 49.7, 44.7, 43.9, 41.3, 31.6, 31.1, 31.0, 13.7. ESI-MS: *m*/*z* = 459.20 ([M + H]^+^).

##### Synthesis of *tert*-Butyl 4-(3-(2,4-Dioxotetrahydropyrimidin-1­(2*H*)-yl)-2-methylphenethyl)-piperidine-1-carboxylate (**I29**)

**I27** (280 mg, 0.677 mmol) was dissolved in dry DMF (10 mL). Pd/C (5%, 72 mg, 0.034 mmol) was added and the argon atmosphere was replaced with a hydrogen atmosphere. The reaction mixture was stirred at rt for 2 h. The reaction mixture was filtered through a pad of Celite and the filter cake was rinsed with methanol (20 mL). The solvents were removed under reduced pressure to give the title compound as a colorless solid (202 mg, 69%). ^1^H NMR (400 MHz, DMSO-*d*_6_) δ: 10.31 (s, 1H), 7.22–7.00 (m, 3H), 3.94 (d, *J* = 12.8 Hz, 2H), 3.78–3.63 (m, 1H), 3.55–3.37 (m, 1H), 2.82–2.58 (m, 6H), 2.10 (s, 3H), 1.72 (d, *J* = 12.9 Hz, 2H), 1.55–1.41 (m, 5H), 1.39 (s, 9H). ESI-MS: *m*/*z* = 438.25 ([M + Na]^+^).

##### Synthesis of 1-(2-Methyl-3-(2-(piperidin-4-yl)­ethyl)­phenyl)­dihydropyrimidine-2,4­(1*H*,3*H*)-dione (**I30**)

The title compound was prepared according to general procedure D, using **I29** (119 mg, 0.286 mmol). The crude product was used without further purification. ESI-MS: *m*/*z* = 338.25 ([M + Na]^+^).

##### Synthesis of 1-(3-(2-(1-(2-Azidoacetyl)­piperidin-4-yl)­ethyl)-2-methylphenyl)­dihydro-pyrimidine-2,4­(1*H*,3*H*)-dione (**X28**)

The title compound was prepared according to general procedure A, using **I30** (90 mg, 0.286 mmol), 2-azidoacetic acid (29 mg, 0.29 mmol), HATU (141 mg, 0.372 mmol) and DIPEA (149 μL, 0.859 mmol). The title compound was obtained as a colorless solid (99 mg, 87%). ^1^H NMR (400 MHz, DMSO-*d*_6_) δ: 10.31 (s, 1H), 7.43–6.71 (m, 3H), 4.35 (d, *J* = 13.0 Hz, 1H), 4.13 (d, *J* = 6.4 Hz, 2H), 3.76–3.68 (m, 1H), 3.63 (d, *J* = 13.4 Hz, 1H), 3.52–3.43 (m, 1H), 2.95 (t, *J* = 12.6 Hz, 1H), 2.82–2.72 (m, 1H), 2.71–2.56 (m, 4H), 2.10 (s, 3H), 1.78 (s, 2H), 1.62–1.49 (m, 0H), 1.53–1.39 (m, 2H), 1.26 (t, *J* = 5.7 Hz, 1H), 1.23–0.96 (m, 2H). ^13^C NMR (101 MHz, DMSO-*d*_6_) δ: 170.7, 165.4, 151.8, 141.8, 141.1, 133.4, 127.9, 126.2, 124.8, 49.7, 44.7, 44.2, 41.7, 36.7, 35.2, 31.9, 31.3, 31.0, 30.3, 13.2. ESI-MS: *m*/*z* = 399.20 ([M + H]^+^).

##### Synthesis of *N*-(2,6-Dioxopiperidin-3-yl)-2-methoxy-4-nitrobenzamide (**I31**)

2-methoxy-4-nitrobenzoic acid (5.14 g, 26.1 mmol), 3-aminopiperidine-2,6-dione hydrochloride (8.58 g, 52.1 mmol), 1-ethyl-3-(3-(dimethylamino)­propyl)­carbodiimide hydrochloride (5.50 g, 28.7 mmol), 1-hydroxybenzotriazole hydrate (4.39 g, 28.7 mmol) were dissolved in dry DMF (40 mL). DIPEA (15.2 mL, 117 mmol) was added and the reaction mixture was stirred at rt for 18 h. The reaction mixture was cooled in an ice bath (0 °C) and diluted with water, filtered and the precipitate was washed with water (50 mL). The precipitate was dried under reduced pressure to give the title compound as a colorless solid (6.72 g, 84%). ^1^H NMR (400 MHz, DMSO-*d*_6_) δ: 10.90 (s, 1H), 8.75 (d, *J* = 7.8 Hz, 1H), 7.97–7.88 (m, 3H), 4.86–4.65 (m, 1H), 4.01 (s, 3H), 2.86–2.70 (m, 1H), 2.58–2.51 (m, 1H), 2.19–1.92 (m, 2H). ESI-MS: *m*/*z* = 308.05 ([M + H]^+^).

##### Synthesis of 4-Amino-*N*-(2,6-dioxopiperidin-3-yl)-2-methoxybenzamide (**I32**)

**I31** (1.29 mg, 4.20 mmol), iron powder (1.17 g, 20.9 mmol) and ammonium chloride (1.12 g, 20.9 mmol) were dissolved in dry methanol (10 mL) and water (5 mL) and the reaction mixture was stirred at 65 °C for 2 h. The reaction mixture was filtered through a pad of Celite and the filter cake was rinsed with methanol (15 mL). The solvent was removed under reduced pressure. The crude product was purified by flash chromatography using acetonitrile/water as an eluent to obtain the title compound as a yellow solid (449 mg, 39%). ^1^H NMR (400 MHz, DMSO-*d*_6_) δ: 10.85 (s, 1H), 8.35 (d, *J* = 6.9 Hz, 1H), 7.66 (d, *J* = 8.5 Hz, 1H), 6.25 (s, 1H), 6.21 (dd, *J* = 8.5, 1.9 Hz, 1H), 5.79 (s, 2H), 4.68 (dt, *J* = 12.3, 6.0 Hz, 1H), 3.83 (s, 3H), 2.81–2.68 (m, 1H), 2.54–2.42 (m, 1H), 2.18–1.97 (m, 2H). ESI-MS: *m*/*z* = 310.10 ([M + H]^+^).

##### Synthesis of 4-(2-Azidoacetamido)-*N*-(2,6-dioxopiperidin-3-yl)-2-methoxybenzamide (**X29**)

The title compound was prepared according to general procedure A, using **I31** (110 mg, 0.397 mmol), 2-azidoacetic acid (40 mg, 0.40 mmol), HATU (196 mg, 0.517 mmol) and DIPEA (208 μL, 1.19 mmol). The title compound was obtained as a colorless solid (115 mg, 80%). ^1^H NMR (400 MHz, DMSO-*d*_6_) δ: 10.88 (s, 1H), 10.41 (s, 1H), 8.54 (d, *J* = 7.3 Hz, 1H), 7.87 (d, *J* = 8.5 Hz, 1H), 7.56 (d, *J* = 1.9 Hz, 1H), 7.22 (dd, *J* = 8.6, 1.9 Hz, 1H), 4.74 (q, *J* = 8.5 Hz, 1H), 4.08 (s, 2H), 3.91 (s, 3H), 2.84–2.69 (m, 1H), 2.56–2.50 (m, 1H), 2.17–2.02 (m, 2H). ^13^C NMR (101 MHz, DMSO-*d*_6_) δ: 172.9, 172.4, 166.9, 163.9, 157.9, 142.6, 131.9, 116.3, 111.0, 102.3, 55.9, 51.3, 50.0, 31.0, 24.1. ESI-MS: *m*/*z* = 361.15 ([M + H]^+^).

##### Synthesis of 4-(3-Azidopropanamido)-*N*-(2,6-dioxopiperidin-3-yl)-2-methoxybenzamide (**X30**)

The title compound was prepared according to general procedure A, using **I31** (200 mg, 0.721 mmol), 3-azidopropanoic acid (125 mg, 1.08 mmol), HATU (356 mg, 0.937 mmol) and DIPEA (377 μL, 2.16 mmol). The title compound was obtained as a colorless solid (156 mg, 58%). ^1^H NMR (400 MHz, DMSO-*d*_6_) δ: 10.88 (s, 1H), 10.33 (s, 1H), 8.53 (d, *J* = 7.2 Hz, 1H), 7.86 (d, *J* = 8.5 Hz, 1H), 7.56 (d, *J* = 1.9 Hz, 1H), 7.23 (dd, *J* = 8.6, 1.9 Hz, 1H), 4.81–4.69 (m, 1H), 3.90 (s, 3H), 3.63 (t, *J* = 6.3 Hz, 2H), 2.83–2.74 (m, 1H), 2.66 (t, *J* = 6.3 Hz, 2H), 2.57–2.52 (m, 1H), 2.15–2.10 (m, 2H). ^13^C NMR (101 MHz, DMSO-*d*_6_) δ: 172.9, 172.4, 169.2, 163.9, 157.9, 143.2, 131.8, 115.9, 110.8, 102.0, 55.8, 50.0, 46.6, 35.7, 30.9, 24.1. ESI-MS: *m*/*z* = 375.10 ([M + H]^+^).

##### Synthesis of 4-(4-Azidobutanamido)-*N*-(2,6-dioxopiperidin-3-yl)-2-methoxybenzamide (**X31**)

The title compound was prepared according to general procedure A, using **I31** (200 mg, 0.721 mmol), 4-azidobutanoic acid (140 mg, 1.08 mmol), HATU (356 mg, 0.937 mmol) and DIPEA (377 μL, 2.16 mmol). The title compound was obtained as a colorless solid (190 mg, 68%). ^1^H NMR (400 MHz, DMSO-*d*_6_) δ: 10.87 (s, 1H), 10.23 (s, 1H), 8.52 (d, *J* = 7.2 Hz, 1H), 7.85 (d, *J* = 8.5 Hz, 1H), 7.58 (d, *J* = 1.9 Hz, 1H), 7.21 (dd, *J* = 8.6, 1.8 Hz, 1H), 4.79–4.63 (m, 1H), 3.89 (s, 3H), 3.40 (t, *J* = 6.8 Hz, 2H), 2.86–2.70 (m, 1H), 2.55–2.52 (m, 1H), 2.44 (t, *J* = 7.3 Hz, 2H), 2.18–2.02 (m, 2H), 1.85 (quin, *J* = 7.0 Hz, 2H). ^13^C NMR (101 MHz, DMSO-*d*_6_) δ: 172.9, 172.4, 170.9, 163.9, 157.9, 143.4, 131.8, 115.6, 110.7, 102.0, 55.8, 50.2, 50.0, 33.3, 30.9, 24.1, 24.0. ESI-MS: *m*/*z* = 389.10 ([M + H]^+^).

##### Synthesis of *N*-(2,6-Dioxopiperidin-3-yl)-4-iodo-2-methoxybenzamide (**I32**)

4-Iodo-2-methoxybenzoic acid (10.0 g, 35.9 mmol), 3-aminopiperidine-2,6-dione hydrochloride (11.84 g, 71.9 mmol), 1-ethyl-3-(3-(dimethylamino)­propyl)­carbodiimide hydrochloride (7.58 g, 39.6 mmol), 1-hydroxybenzotriazole hydrate (6.06 g, 39.6 mmol) were dissolved in dry DMF (50 mL). DIPEA (28.1 mL, 162 mmol) was added and the reaction mixture was stirred at rt for 18 h. The reaction mixture was cooled in an ice bath (0 °C) and diluted with water, filtered and the precipitate was washed with water (50 mL). The precipitate was dried under reduced pressure to give the title compound as a light gray solid (12.3 g, 89%). ^1^H NMR (400 MHz, DMSO-*d*_6_) δ: 10.87 (s, 1H), 8.56 (d, *J* = 7.5 Hz, 1H), 7.57 (d, *J* = 8.1 Hz, 1H), 7.50 (d, *J* = 1.5 Hz, 1H), 7.45 (dd, *J* = 8.1, 1.5 Hz, 1H), 4.78–4.68 (m, 1H), 3.92 (s, 3H), 2.83–2.70 (m, 1H), 2.54 (t, *J* = 3.7 Hz, 1H), 2.16–2.00 (m, 2H). ESI-MS: *m*/*z* = 388.95 ([M + H]^+^).

##### Synthesis of *tert*-Butyl 3-((4-((2,6-Dioxopiperidin-3-yl)­carbamoyl)-3-methoxyphenyl)­amino)­azetidine-1-carboxylate (**I33**)

**I32** (1.50 g, 3.86 mmol), *tert*-butyl 3-aminoazetidine-1-carboxylate (1.33 g, 7.73 mmol), copper iodide (147 mg, 0.775 mmol), l-proline (178 mg, 1.55 mmol) and potassium carbonate (1.60 g, 11.6 mmol) were dissolved in dry DMSO (10 mL) and stirred at 80 °C for 18 h. The solvent was removed under reduced pressure and the crude product was purified by flash chromatography using acetonitrile/water as an eluent. The title compound was obtained as a colorless solid (830 mg, 50%). ^1^H NMR (400 MHz, DMSO-*d*_6_) δ: 10.85 (s, 1H), 8.02 (d, *J* = 6.6 Hz, 1H), 7.93 (d, *J* = 8.6 Hz, 1H), 6.18 (dd, *J* = 8.6, 2.1 Hz, 1H), 6.07 (d, *J* = 2.1 Hz, 1H), 4.76–4.61 (m, 2H), 4.32–4.18 (m, 5H), 3.93 (s, 3H), 3.81–3.69 (m, 4H), 1.42 (s, 9H). ESI-MS: *m*/*z* = 433.15 ([M + H]^+^).

##### Synthesis of 4-(Azetidin-3-ylamino)-*N*-(2,6-dioxopiperidin-3-yl)-2-methoxybenzamide (**I34**)

The title compound was prepared according to general procedure D, using **I33** (800 mg, 0.185 mmol). The crude product was used without further purification. ESI-MS: *m*/*z* = 333.20 ([M + H]^+^).

##### Synthesis of 4-((1-(2-Azidoacetyl)­azetidin-3-yl)­amino)-*N*-(2,6-dioxopiperidin-3-yl)-2-methoxybenzamide (**X32**)

The title compound was prepared according to general procedure A, using **I34** (130 mg, 0.352 mmol), 2-azidoacetic acid (39 mg, 0.39 mmol), HATU (201 mg, 0.582 mmol) and DIPEA (245 μL, 1.41 mmol). The title compound was obtained as a colorless solid (140 mg, 96%). ^1^H NMR (400 MHz, DMSO-*d*_6_) δ: 10.86 (s, 1H), 8.38 (d, *J* = 6.9 Hz, 1H), 7.74 (d, *J* = 8.4 Hz, 1H), 6.99 (d, *J* = 5.7 Hz, 1H), 6.23–6.13 (m, 2H), 4.69 (dt, *J* = 12.5, 6.3 Hz, 1H), 4.48 (t, *J* = 7.6 Hz, 1H), 4.36–4.23 (m, 2H), 3.91 (s, 2H), 3.88 (s, 3H), 3.76 (dd, *J* = 9.1, 4.0 Hz, 1H), 2.89 (s, 1H), 2.75 (ddd, *J* = 17.2, 13.3, 5.7 Hz, 1H), 2.54–2.52 (m, 1H), 2.21–1.93 (m, 2H). ^13^C NMR (101 MHz, DMSO-*d*_6_) δ: 172.9, 172.7, 167.2, 164.4, 159.2, 151.3, 132.8, 109.5, 104.6, 95.0, 60.3, 56.8, 55.6, 54.9, 50.0, 47.9, 42.3, 31.0, 24.4. ESI-MS: *m*/*z* = 416.25 ([M + H]^+^).

##### Synthesis of 4-((1-(3-Azidopropanoyl)­azetidin-3-yl)­amino)-*N*-(2,6-dioxopiperidin-3-yl)-2-methoxybenzamide (**X33**)

The title compound was prepared according to general procedure A, using **I34** (130 mg, 0.352 mmol), 3-azidopropanoic acid (45 mg, 0.39 mmol), HATU (201 mg, 0.582 mmol) and DIPEA (245 μL, 1.41 mmol). The title compound was obtained as a colorless solid (149 mg, 99%). ^1^H NMR (400 MHz, DMSO-*d*_6_) δ: 10.86 (s, 1H), 8.38 (d, *J* = 7.0 Hz, 1H), 7.74 (d, *J* = 8.5 Hz, 1H), 6.98 (d, *J* = 6.1 Hz, 1H), 6.25–5.99 (m, 2H), 4.76–4.63 (m, 1H), 4.51 (t, *J* = 7.8 Hz, 1H), 4.36–4.28 (m, 1H), 4.25 (t, *J* = 9.4, 7.5 Hz, 1H), 3.91 (d, *J* = 4.7 Hz, 1H), 3.88 (s, 3H), 3.74–3.69 (m, 1H), 3.51 (t, *J* = 6.3 Hz, 2H), 2.75 (ddd, *J* = 17.2, 13.5, 5.7 Hz, 1H), 2.55–2.51 (m, 1H), 2.37 (t, *J* = 6.3 Hz, 2H), 2.20–1.94 (m, 2H). ^13^C NMR (101 MHz, DMSO-*d*_6_) δ: 172.9, 172.7, 169.8, 164.4, 159.2, 151.4, 132.8, 118.0, 109.4, 104.6, 95.0, 56.9, 55.6, 54.4, 50.1, 46.3, 41.7, 31.0, 30.2, 24.4. ESI-MS: *m*/*z* = 430.15 ([M + H]^+^).

##### Synthesis of 4-((1-(4-Azidobutanoyl)­azetidin-3-yl)­amino)-*N*-(2,6-dioxopiperidin-3-yl)-2-methoxybenzamide (**X34**)

The title compound was prepared according to general procedure A, using **I34** (130 mg, 0.352 mmol), 4-azidobutanoic acid (50 mg, 0.39 mmol), HATU (201 mg, 0.582 mmol) and DIPEA (245 μL, 1.41 mmol). The title compound was obtained as a colorless solid (127 mg, 77%). ^1^H NMR (400 MHz, DMSO-*d*_6_) δ: 10.86 (s, 1H), 8.38 (d, *J* = 7.0 Hz, 1H), 7.73 (d, *J* = 8.5 Hz, 1H), 6.96 (d, *J* = 6.2 Hz, 1H), 6.28–6.11 (m, 2H), 4.69 (quin, *J* = 6.2 Hz, 1H), 4.49 (t, *J* = 7.7 Hz, 1H), 4.33–4.25 (m, 1H), 4.25–4.18 (m, 1H), 3.88 (s, 3H), 3.87–3.84 (m, 2H), 3.67 (dd, *J* = 9.6, 4.8 Hz, 1H), 2.75 (ddd, *J* = 17.2, 13.4, 5.7 Hz, 1H), 2.52 (s, 1H), 2.20–1.99 (m, 5H), 1.73 (quin, *J* = 7.1 Hz, 2H). ^13^C NMR (101 MHz, DMSO-*d*_6_) δ: 172.9, 172.7, 171.4, 164.4, 159.2, 151.4, 132.8, 109.5, 104.6, 95.0, 56.7, 55.6, 54.3, 50.1, 50.0, 41.7, 31.0, 27.5, 24.5, 23.5. ESI-MS: *m*/*z* = 444.20 ([M + H]^+^).

##### Synthesis of *tert*-Butyl 4-(4-((2,6-Dioxopiperidin-3-yl)­carbamoyl)-3-methoxyphenyl)-piperazine-1-carboxylate (**I35**)

**I32** (1.50 g, 3.86 mmol), *tert*-butyl piperazine-1-carboxylate (1.44 g, 7.73 mmol), copper iodide (147 mg, 0.775 mmol), l-proline (178 mg, 1.55 mmol) and potassium carbonate (1.60 g, 11.6 mmol) were dissolved in dry DMSO (10 mL) and stirred at 80 °C for 18 h. The solvent was removed under reduced pressure and the crude product was purified by flash chromatography using acetonitrile/water as an eluent. The title compound was obtained as a colorless solid (415 mg, 24%). ^1^H NMR (400 MHz, DMSO-*d*_6_) δ: 10.87 (s, 1H), 8.44 (d, *J* = 7.0 Hz, 1H), 7.79 (d, *J* = 8.8 Hz, 1H), 6.60 (dd, *J* = 8.9, 2.2 Hz, 1H), 6.56 (d, *J* = 2.3 Hz, 1H), 4.79–4.61 (m, 1H), 3.92 (s, 3H), 3.50–3.41 (m, 4H), 3.31–3.27 (m, 4H), 2.82–2.67 (m, 1H), 2.57–2.51 (m, 1H), 2.19–1.96 (m, 2H), 1.42 (s, 9H). ESI-MS: *m*/*z* = 447.15 ([M + H]^+^).

##### Synthesis of *N*-(2,6-Dioxopiperidin-3-yl)-2-methoxy-4-(piperazin-1-yl)­benzamide (**I36**)

The title compound was prepared according to general procedure D, using **I35** (415 mg, 0.929 mmol). The crude product was used without further purification. ESI-MS: *m*/*z* = 347.05 ([M + H]^+^).

##### Synthesis of 4-(4-(2-Azidoacetyl)­piperazin-1-yl)-*N*-(2,6-dioxopiperidin-3-yl)-2-methoxy-benzamide (**X35**)

The title compound was prepared according to general procedure A, using **I36** (115 mg, 0.301 mmol), 2-azidoacetic acid (33 mg, 0.33 mmol), HATU (171 mg, 0.451 mmol) and DIPEA (157 μL, 0.902 mmol). The title compound was obtained as a colorless solid (78 mg, 60%). ^1^H NMR (400 MHz, DMSO-*d*_6_) δ: 10.87 (s, 1H), 8.45 (d, *J* = 7.0 Hz, 1H), 7.80 (d, *J* = 8.8 Hz, 1H), 6.61 (dd, *J* = 8.9, 2.2 Hz, 1H), 6.56 (d, *J* = 2.2 Hz, 1H), 4.70 (dt, *J* = 12.4, 6.3 Hz, 1H), 4.21 (s, 2H), 3.93 (s, 3H), 3.73–3.59 (m, 2H), 3.53–3.44 (m, 2H), 3.39–3.34 (m, 4H), 2.91–2.66 (m, 1H), 2.55–2.52 (m, 1H), 2.19–1.94 (m, 2H). ^13^C NMR (101 MHz, DMSO-*d*_6_) δ: 172.9, 172.6, 166.1, 164.2, 158.9, 154.0, 132.4, 110.9, 106.7, 97.8, 55.9, 50.1, 49.6, 46.8, 46.7, 43.5, 41.0, 31.0, 24.3. ESI-MS: *m*/*z* = 430.20 ([M + H]^+^).

##### Synthesis of 4-(4-(4-Azidobutanoyl)­piperazin-1-yl)-*N*-(2,6-dioxopiperidin-3-yl)-2-methoxybenzamide (**X36**)

The title compound was prepared according to general procedure A, using **I36** (200 mg, 0.721 mmol), 4-azidobutanoic acid (125 mg, 1.08 mmol), HATU (356 mg, 0.937 mmol) and DIPEA (377 μL, 2.16 mmol). The title compound was obtained as a colorless solid (156 mg, 58%). ^1^H NMR (400 MHz, DMSO-*d*_6_) δ: 10.86 (s, 1H), 8.44 (d, *J* = 7.0 Hz, 1H), 7.80 (d, *J* = 8.8 Hz, 1H), 6.61 (dd, *J* = 8.9, 2.2 Hz, 1H), 6.56 (d, *J* = 2.2 Hz, 1H), 4.70 (ddd, *J* = 12.4, 7.0, 5.6 Hz, 1H), 3.93 (s, 3H), 3.60 (q, *J* = 5.1 Hz, 4H), 3.37 (t, *J* = 6.9 Hz, 4H), 3.31–3.28 (m, 2H), 2.76 (ddd, *J* = 17.3, 13.4, 5.8 Hz, 1H), 2.56–2.51 (m, 1H), 2.44 (t, *J* = 7.2 Hz, 2H), 2.21–1.99 (m, 2H), 1.78 (quin, *J* = 7.1 Hz, 2H). ^13^C NMR (101 MHz, DMSO-*d*_6_) δ: 172.9, 172.6, 169.9, 164.2, 158.8, 154.1, 132.3, 110.9, 106.6, 97.7, 55.9, 50.2, 50.0, 47.1, 46.8, 44.2, 40.6, 31.0, 29.0, 24.3, 24.0. ESI-MS: *m*/*z* = 458.15 ([M + H]^+^).

##### Synthesis of (2*S*,4*R*)-1-((*S*)-2-(4-Azidobenzamido)-3,3-dimethylbutanoyl)-4-hydroxy-*N*-(4-(4-methylthiazol-5-yl)­benzyl)­pyrrolidine-2-carboxamide (**X37**)

The title compound was prepared according to general procedure A, using (2*S*,4*R*)-1-((*S*)-2-amino-3,3-dimethylbutanoyl)-4-hydroxy-*N*-(4-(4-methylthiazol-5-yl)­benzyl)­pyrrolidine-2-carboxamide (100 mg, 0.232 mmol), 4-azidobenzoic acid (38 mg, 0.23 mmol), HATU (115 mg, 0.302 mmol) and DIPEA (121 μL, 0.697 mmol). The title compound was obtained as a colorless solid (110 mg, 82%). ^1^H NMR (400 MHz, DMSO-*d*_6_) δ: 8.98 (s, 1H), 8.58 (t, *J* = 6.1 Hz, 1H), 8.05 (d, *J* = 9.0 Hz, 1H), 7.94 (d, *J* = 8.2 Hz, 2H), 7.44–7.36 (m, 4H), 7.18 (d, *J* = 8.2 Hz, 2H), 5.15 (d, *J* = 3.6 Hz, 1H), 4.77 (d, *J* = 9.0 Hz, 1H), 4.50–4.35 (m, 3H), 4.24 (dd, *J* = 15.9, 5.5 Hz, 1H), 3.73 (s, 2H), 2.45 (s, 3H), 2.16–1.85 (m, 2H), 1.03 (s, 9H). ^13^C NMR (101 MHz, DMSO-*d*_6_) δ: 171.91, 169.45, 165.60, 151.47, 147.7, 142.3, 139.4, 131.1, 130.6, 129.6, 128.6, 127.4, 118.6, 68.9, 58.8, 57.3, 56.4, 41.6, 37.9, 35.5, 26.5, 15.9. ESI-MS: *m*/*z* = 476.20 ([M + H]^+^).

##### Synthesis of (2*S*,4*R*)-1-((*S*)-2-(2-Azidoacetamido)-3,3-dimethylbutanoyl)-4-hydroxy-*N*-(4-(4-methylthiazol-5-yl)­benzyl)­pyrrolidine-2-carboxamide (**X38**)

The title compound was prepared according to general procedure A, using (2*S*,4*R*)-1-((*S*)-2-amino-3,3-dimethylbutanoyl)-4-hydroxy-*N*-(4-(4-methylthiazol-5-yl)­benzyl)­pyrrolidine-2-carboxamide (100 mg, 0.232 mmol), 2-azidoacetic acid (23 mg, 0.23 mmol), HATU (115 mg, 0.302 mmol) and DIPEA (121 μL, 0.697 mmol). The title compound was obtained as a colorless solid (105 mg, 88%). ^1^H NMR (400 MHz, DMSO-*d*_6_) δ: 8.98 (s, 1H), 8.60 (t, *J* = 6.0 Hz, 1H), 8.23 (d, *J* = 9.3 Hz, 1H), 7.45–7.35 (m, 4H), 5.17 (d, *J* = 3.5 Hz, 1H), 4.56 (d, *J* = 9.3 Hz, 1H), 4.48–4.40 (m, 2H), 4.36 (s, 1H), 4.21 (dd, *J* = 15.9, 5.4 Hz, 1H), 3.99–3.84 (m, 2H), 3.73–3.59 (m, 2H), 2.44 (s, 3H), 2.04 (d, *J* = 8.6 Hz, 1H), 1.95–1.86 (m, 1H), 0.95 (s, 9H). ^13^C NMR (101 MHz, DMSO-*d*_6_) δ: 171.8, 169.1, 167.3, 151.4, 147.7, 139.5, 131.1, 129.6, 128.6, 127.4, 68.9, 58.7, 56.5, 50.3, 41.6, 37.9, 35.4, 26.2, 15.9. ESI-MS: *m*/*z* = 514.20 ([M + H]^+^).

##### Synthesis of (2*S*,4*R*)-1-((*S*)-2-(4-Azidobutanamido)-3,3-dimethylbutanoyl)-4-hydroxy-*N*-(4-(4-methylthiazol-5-yl)­benzyl)­pyrrolidine-2-carboxamide (**X39**)

The title compound was prepared according to general procedure A, using (2*S*,4*R*)-1-((*S*)-2-amino-3,3-dimethylbutanoyl)-4-hydroxy-*N*-(4-(4-methylthiazol-5-yl)­benzyl)­pyrrolidine-2-carboxamide (100 mg, 0.232 mmol), 4-azidobutanoic acid (30 mg, 0.23 mmol), HATU (115 mg, 0.302 mmol) and DIPEA (121 μL, 0.697 mmol). The title compound was obtained as a colorless solid (114 mg, 91%). ^1^H NMR (400 MHz, DMSO-*d*_6_) δ: 8.97 (s, 1H), 8.56 (t, *J* = 6.1 Hz, 1H), 7.97 (d, *J* = 9.3 Hz, 1H), 7.46–7.27 (m, 4H), 5.14 (d, *J* = 3.5 Hz, 1H), 4.54 (d, *J* = 9.3 Hz, 1H), 4.47–4.39 (m, 2H), 4.36–4.32 (m, 1H), 4.21 (dd, *J* = 15.9, 5.4 Hz, 1H), 3.72–3.58 (m, 2H), 3.30 (td, *J* = 6.9, 1.1 Hz, 2H), 2.44 (s, 3H), 2.40–2.18 (m, 2H), 2.10–1.98 (m, 1H), 1.95–1.85 (m, 1H), 1.81–1.66 (m, 2H), 0.93 (s, 9H). ^13^C NMR (101 MHz, DMSO-*d*_6_) δ: 172.0, 171.3, 169.6, 151.5, 147.8, 139.5, 131.2, 129.7, 128.7, 127.4, 68.9, 58.7, 56.5, 56.4, 50.3, 41.7, 37.9, 35.2, 31.8, 26.4, 24.7, 15.9. ESI-MS: *m*/*z* = 542.35 ([M + H]^+^).

##### Synthesis of Methyl 4-(((*S*)-1-((2*S*,4*R*)-4-Hydroxy-2-((4-(4-methylthiazol-5-yl)­benzyl)­carbamoyl)­pyrrolidin-1-yl)-3,3-dimethyl-1-oxobutan-2-yl)­carbamoyl)­benzoate (**I37**)

The title compound was prepared according to general procedure A, using (2*S*,4*R*)-1-((*S*)-2-amino-3,3-dimethylbutanoyl)-4-hydroxy-*N*-(4-(4-methylthiazol-5-yl)­benzyl)­pyrrolidine-2-carboxamide (1.00 g, 2.32 mmol), 4-(methoxycarbonyl)­benzoic acid (418 mg, 2.32 mmol), HATU (1.15 g, 3.02 mmol) and DIPEA (1.21 mL, 6.97 mmol). The title compound was obtained as a colorless solid (1.29 g, 94%). ^1^H NMR (400 MHz, DMSO-*d*_6_) δ: 8.98 (s, 1H), 8.58 (t, *J* = 6.0 Hz, 1H), 8.29 (d, *J* = 9.1 Hz, 1H), 8.10–7.92 (m, 4H), 7.45–7.35 (m, 4H), 5.15 (d, *J* = 3.6 Hz, 1H), 4.78 (d, *J* = 9.1 Hz, 1H), 4.48–4.40 (m, 2H), 4.39–4.35 (m, 1H), 4.24 (dd, *J* = 15.8, 5.5 Hz, 1H), 3.88 (s, 3H), 3.73 (d, *J* = 3.0 Hz, 2H), 2.45 (s, 3H), 2.11–2.00 (m, 1H), 1.98–1.86 (m, 1H), 1.04 (s, 9H). ESI-MS: *m*/*z* = 615.20 ([M + Na]^+^).

##### Synthesis of 4-(((*S*)-1-((2*S*,4*R*)-4-Hydroxy-2-((4-(4-methylthiazol-5-yl)­benzyl)­carbamoyl)­pyrrolidin-1-yl)-3,3-dimethyl-1-oxobutan-2-yl)­carbamoyl)­benzoic Acid (**I38**)

**I37** (1.12 g, 1.83 mmol) and LiOH·H2O (308 mg, 7.34 mmol) were dissolved in MeOH (10 mL) and H_2_O (5 mL) and stirred at rt for 16 h. Afterward, the pH was brought to 1 through the addition of aqueous HCl (10%) and most of the organic solvent was removed under reduced pressure. The residue was partitioned between water and ethyl acetate and the aqueous phase was extracted with ethyl acetate (3x). The combined organic phase was washed with brine, dried over MgSO_4_, filtered, and volatiles were removed under reduced pressure to provide the title compound (1.03 g, 97%). ^1^H NMR (400 MHz, DMSO-*d*_6_) δ: 13.00 (s, 1H), 8.98 (s, 1H), 8.59 (t, *J* = 6.1 Hz, 1H), 8.24 (d, *J* = 9.1 Hz, 1H), 8.03–7.93 (m, 4H), 7.45–7.31 (m, 4H), 4.78 (d, *J* = 9.1 Hz, 1H), 4.51–4.35 (m, 3H), 4.24 (dd, *J* = 15.8, 5.5 Hz, 1H), 3.74 (d, *J* = 3.0 Hz, 2H), 2.45 (s, 3H), 2.09–2.02 (m, 1H), 1.97–1.91 (m, 1H), 1.04 (s, 9H). ESI-MS: *m*/*z* = 579.30 ([M + H]^+^).

##### Synthesis of *N* ^1^-(3-Azidopropyl)-*N* ^4^-((*S*)-1-((2*S*,4*R*)-4-hydroxy-2-((4-(4-methylthiazol-5-yl)­benzyl)­carbamoyl)­pyrrolidin-1-yl)-3,3-dimethyl-1-oxobutan-2-yl)­terephthalamide (**X40**)

The title compound was prepared according to general procedure A, using **I38** (180 mg, 0.311 mmol), 3-azidopropan-1-amine (31 mg, 0.31 mmol), HATU (154 mg, 0.404 mmol) and DIPEA (163 μL, 0.933 mmol). The title compound was obtained as a colorless solid (191 mg, 93%). ^1^H NMR (400 MHz, DMSO-*d*_6_) δ: 8.98 (s, 1H), 8.63 (dt, *J* = 24.2, 5.9 Hz, 2H), 8.17 (d, *J* = 9.0 Hz, 1H), 7.93 (q, *J* = 8.4 Hz, 4H), 7.46–7.32 (m, 4H), 5.18 (d, *J* = 3.6 Hz, 1H), 4.80 (d, *J* = 9.1 Hz, 1H), 4.53–4.36 (m, 3H), 4.25 (dd, *J* = 15.7, 5.5 Hz, 1H), 3.75 (s, 2H), 3.42 (t, *J* = 6.8 Hz, 2H), 3.35 (q, *J* = 6.7 Hz, 2H), 2.45 (s, 3H), 2.13–2.00 (m, 1H), 1.99–1.89 (m, 1H), 1.80 (quin, *J* = 6.8 Hz, 2H), 1.05 (s, 9H). ^13^C NMR (101 MHz, DMSO-*d*_6_) δ: 171.9, 169.4, 166.0, 165.7, 151.4, 147.8, 139.5, 136.8, 136.3, 131.2, 129.7, 128.7, 127.7, 127.5, 127.0, 68.9, 58.8, 57.4, 56.5, 48.6, 41.7, 38.2, 37.9, 36.7, 35.6, 28.3, 26.5, 15.9. ESI-MS: *m*/*z* = 661.35 ([M + H]^+^).

##### Synthesis of *N* ^1^-(4-Azidobutyl)-*N* ^4^-((*S*)-1-((2*S*,4*R*)-4-hydroxy-2-((4-(4-methylthiazol-5-yl)­benzyl)­carbamoyl)­pyrrolidin-1-yl)-3,3-dimethyl-1-oxobutan-2-yl)­terephthalamide (**X41**)

The title compound was prepared according to general procedure A, using **I38** (180 mg, 0.311 mmol), 4-azidobutan-1-amine (36 mg, 0.31 mmol), HATU (154 mg, 0.404 mmol) and DIPEA (163 μL, 0.933 mmol). The title compound was obtained as a colorless solid (190 mg, 90%). ^1^H NMR (400 MHz, DMSO-*d*_6_) δ: 8.98 (s, 1H), 8.60 (dt, *J* = 13.8, 5.9 Hz, 2H), 8.15 (d, *J* = 9.0 Hz, 1H), 7.98–7.85 (m, 4H), 7.46–7.37 (m, 4H), 5.15 (d, *J* = 3.6 Hz, 1H), 4.79 (d, *J* = 9.1 Hz, 1H), 4.50–4.37 (m, 3H), 4.25 (dd, *J* = 15.8, 5.6 Hz, 1H), 3.74 (d, *J* = 3.1 Hz, 2H), 3.38–3.35 (m, 2H), 3.31–3.26 (m, 2H), 2.45 (s, 3H), 2.12–2.02 (m, 1H), 1.98–1.86 (m, 1H), 1.59 (quin, *J* = 3.4 Hz, 4H), 1.04 (s, 9H). ^13^C NMR (101 MHz, DMSO-*d*_6_) δ: 171.8, 169.3, 165.9, 165.4, 151.4, 147.7, 139.4, 136.8, 136.2, 131.1, 129.6, 128.6, 127.6, 127.4, 126.9, 68.9, 58.8, 57.4, 56.4, 50.3, 41.6, 38.6, 37.9, 35.5, 26.5, 26.3, 25.8, 15.9. ESI-MS: *m*/*z* = 675.40 ([M + H]^+^).

##### Synthesis of *N* ^1^-(6-Azidohexyl)-*N* ^4^-((*S*)-1-((2*S*,4*R*)-4-hydroxy-2-((4-(4-methylthiazol-5-yl)­benzyl)­carbamoyl)­pyrrolidin-1-yl)-3,3-dimethyl-1-oxobutan-2-yl)­terephthalamide (**X42**)

The title compound was prepared according to general procedure A, using **I38** (180 mg, 0.311 mmol), 6-azidohexan-1-amine (44 mg, 0.31 mmol), HATU (154 mg, 0.404 mmol) and DIPEA (163 μL, 0.933 mmol). The title compound was obtained as a colorless solid (179 mg, 58%). ^1^H NMR (400 MHz, DMSO-*d*_6_) δ: 8.98 (s, 1H), 8.57 (q, *J* = 6.3 Hz, 2H), 8.15 (d, *J* = 9.0 Hz, 1H), 7.92 (q, *J* = 8.4 Hz, 4H), 7.53–7.34 (m, 4H), 5.15 (d, *J* = 3.6 Hz, 1H), 4.79 (d, *J* = 9.1 Hz, 1H), 4.53–4.35 (m, 3H), 4.25 (dd, *J* = 15.8, 5.5 Hz, 1H), 3.74 (s, 2H), 3.31–3.23 (m, 4H), 2.45 (s, 3H), 2.14–2.02 (m, 1H), 1.99–1.89 (m, 1H), 1.54 (quin, *J* = 6.2 Hz, 4H), 1.37–1.29 (m, 4H), 1.05 (s, 9H). ^13^C NMR (101 MHz, DMSO-*d*_6_) δ: 171.8, 169.3, 165.9, 165.3, 151.4, 147.7, 139.4, 136.9, 136.1, 131.1, 129.6, 128.6, 127.6, 127.4, 126.9, 68.9, 58.8, 57.4, 56.4, 50.5, 41.6, 37.9, 35.5, 28.8, 28.1, 26.5, 26.0, 25.8, 15.9. ESI-MS: *m*/*z* = 703.40 ([M + H]^+^).

##### Synthesis of *N* ^1^-(2-(2-(2-Azidoethoxy)­ethoxy)­ethyl)-*N* ^4^-((*S*)-1-((2*S*,4*R*)-4-hydroxy-2-((4-(4-methylthiazol-5-yl)­benzyl)­carbamoyl)­pyrrolidin-1-yl)-3,3-dimethyl-1-oxobutan-2-yl)­terephthalamide (**X43**)

The title compound was prepared according to general procedure B, using **I38** (300 mg, 0.518 mmol) and 1-(2-aminoethoxy)-2-(2-azidoethoxy)­ethane (99 mg, 0.57 mmol), 1-methyl-1*H*-imidazole (145 μL, 1.81 mmol) and TCFH (175 mg, 0.622 mmol). The title compound was obtained as a colorless solid (137 mg, 36%). ^1^H NMR (500 MHz, CDCl_3_) δ: 8.68 (s, 1H), 7.86–7.75 (m, 4H), 7.42–7.32 (m, 4H), 7.23 (t, *J* = 6.1 Hz, 1H), 6.86–6.80 (m, 2H), 4.79–4.70 (m, 2H), 4.63–4.53 (m, 2H), 4.35 (dd, *J* = 14.9, 5.3 Hz, 1H), 4.16 (d, *J* = 11.2 Hz, 1H), 3.70–3.62 (m, 12H), 3.36 (t, *J* = 4.9 Hz, 2H), 2.66–2.57 (m, 1H), 2.52 (s, 3H), 2.17–2.10 (m, 1H), 1.01 (s, 9H). ^13^C NMR (126 MHz, CDCl_3_) δ: 171.8, 170.5, 167.1, 166.6, 150.4, 148.6, 138.1, 137.8, 136.1, 131.6, 131.2, 129.7, 128.3, 127.5, 127.4, 70.6, 70.4, 70.3, 70.2, 69.9, 58.6, 58.1, 56.9, 50.7, 43.5, 40.0, 35.9, 35.5, 26.6, 16.2. ESI-MS: *m*/*z* = 735.4 ([M + H]^+^).

##### Synthesis of *N* ^1^-(2-(2-(2-(2-Azidoethoxy)­ethoxy)­ethoxy)­ethyl)-*N* ^4^-((*S*)-1-((2*S*,4*R*)-4-hydroxy-2-((4-(4-methylthiazol-5-yl)­benzyl)­carbamoyl)­pyrrolidin-1-yl)-3,3-dimethyl-1-oxobutan-2-yl)­terephthalamide (**X44**)

The title compound was prepared according to general procedure B, using **I38** (200 mg, 0.346 mmol) and 1-[2-(2-aminoethoxy)­ethoxy]-2-(2-azidoethoxy)­ethane (83 mg, 0.38 mmol), 1-methyl-1*H*-imidazole (96 μL, 1.2 mmol) and TCFH (116 mg, 0.415 mmol). The title compound was obtained as a colorless solid (199 mg, 74%). ^1^H NMR (500 MHz, CDCl_3_) δ: 8.68 (s, 1H), 7.91–7.64 (m, 4H), 7.42–7.33 (m, 4H), 7.24 (t, *J* = 5.9 Hz, 1H), 7.04 (t, *J* = 4.5 Hz, 1H), 6.90 (d, *J* = 8.8 Hz, 1H), 4.77–4.69 (m, 2H), 4.57 (dd, *J* = 14.8, 6.4 Hz, 2H), 4.36 (dd, *J* = 14.9, 5.3 Hz, 1H), 4.15 (dt, *J* = 11.5, 1.8 Hz, 1H), 3.73–3.57 (m, 16H), 3.33 (t, *J* = 5.0 Hz, 2H), 2.62–2.53 (m, 1H), 2.52 (s, 3H), 2.17–2.09 (m, 1H), 1.00 (s, 9H). ^13^C NMR (126 MHz, CDCl_3_) δ: 171.8, 170.6, 166.9, 166.6, 150.4, 148.6, 138.1, 137.8, 136.0, 131.6, 131.2, 129.7, 128.3, 127.5, 127.4, 70.8, 70.7, 70.6, 70.3, 70.3, 70.1, 69.8, 58.6, 58.0, 57.0, 50.7, 43.4, 40.0, 36.0, 35.6, 26.6, 16.2. ESI-MS: *m*/*z* = 779.4 ([M + H]^+^).

##### Synthesis of *tert*-Butyl 4-(((*S*)-1-((2*S*,4*R*)-4-Hydroxy-2-((4-(4-methylthiazol-5-yl)­benzyl)­carbamoyl)­pyrrolidin-1-yl)-3,3-dimethyl-1-oxobutan-2-yl)­amino)-4-oxobutanoate (**I39**)

The title compound was prepared according to general procedure A, using (2*S*,4*R*)-1-((*S*)-2-amino-3,3-dimethylbutanoyl)-4-hydroxy-*N*-(4-(4-methylthiazol-5-yl)­benzyl)­pyrrolidine-2-carboxamide (1.00 g, 2.32 mmol), 4-(*tert*-butoxy)-4-oxobutanoic acid (405 mg, 2.32 mmol), HATU (1.15 g, 3.02 mmol) and DIPEA (1.21 mL, 6.97 mmol). The title compound was obtained as a colorless solid (934 mg, 69%). ^1^H NMR (400 MHz, DMSO-*d*_6_) δ: 8.98 (s, 1H), 8.56 (t, *J* = 6.0 Hz, 1H), 7.93 (d, *J* = 9.4 Hz, 1H), 7.46–7.33 (m, 4H), 5.11 (d, *J* = 3.5 Hz, 1H), 4.54 (d, *J* = 9.3 Hz, 1H), 4.47–4.39 (m, 2H), 4.35 (s, 1H), 4.21 (dd, *J* = 15.9, 5.4 Hz, 1H), 3.70–3.54 (m, 2H), 2.44 (s, 3H), 2.41–2.28 (m, 4H), 2.06–1.98 (m, 1H), 1.95–1.85 (m, 1H), 1.37 (s, 9H), 0.93 (s, 9H). ESI-MS: *m*/*z* = 609.30 ([M + Na]^+^).

##### Synthesis of 4-(((*S*)-1-((2*S*,4*R*)-4-Hydroxy-2-((4-(4-methylthiazol-5-yl)­benzyl)­carbamoyl)-pyrrolidin-1-yl)-3,3-dimethyl-1-oxobutan-2-yl)­amino)-4-oxobutanoic Acid (**I40**)

The title compound was prepared according to general procedure D, using **I39** (467 mg, 0.796 mmol). The crude product was used without further purification. ESI-MS: *m*/*z* = 531.25 ([M + H]^+^).

##### Synthesis of *N* ^1^-(3-Azidopropyl)-*N* ^4^-((*S*)-1-((2*S*,4*R*)-4-hydroxy-2-((4-(4-methylthiazol-5-yl)­benzyl)­carbamoyl)­pyrrolidin-1-yl)-3,3-Dimethyl-1-oxobutan-2-yl)­succinamide (**X45**)

The title compound was prepared according to general procedure A, using **I40** (96 mg, 0.18 mmol), 3-azidopropan-1-amine (18 mg, 0.18 mmol), HATU (90 mg, 0.24 mmol) and DIPEA (95 μL, 0.54 mmol). The title compound was obtained as a colorless solid (99 mg, 89%). ^1^H NMR (400 MHz, DMSO-*d*_6_) δ: 8.98 (s, 1H), 8.56 (t, *J* = 6.0 Hz, 1H), 7.97–7.82 (m, 2H), 7.44–7.36 (m, 4H), 5.12 (d, *J* = 3.5 Hz, 1H), 4.52 (d, *J* = 9.3 Hz, 1H), 4.47–4.38 (m, 2H), 4.35 (s, 1H), 4.22 (dd, *J* = 15.8, 5.5 Hz, 1H), 3.71–3.55 (m, 2H), 3.36–3.34 (m, 3H), 3.08 (qd, *J* = 6.7, 2.6 Hz, 2H), 2.44 (s, 3H), 2.40–2.21 (m, 3H), 2.07–2.00 (m, 1H), 1.94–1.86 (m, 1H), 1.63 (quin, *J* = 6.8 Hz, 2H), 0.93 (s, 9H). ^13^C NMR (101 MHz, DMSO-*d*_6_) ^13^C NMR (101 MHz, DMSO) δ: 171.9, 171.4, 169.5, 151.4, 147.7, 139.5, 131.1, 129.6, 128.6, 127.4, 68.9, 58.7, 56.4, 41.6, 37.9, 35.8, 35.3, 30.9, 30.5, 28.4, 26.3. ESI-MS: *m*/*z* = 635.30 ([M + Na]^+^).

##### Synthesis of *tert*-Butyl 5-(((*S*)-1-((2*S*,4*R*)-4-Hydroxy-2-((4-(4-methylthiazol-5-yl)­benzyl)­carbamoyl)­pyrrolidin-1-yl)-3,3-dimethyl-1-oxobutan-2-yl)­amino)-5-oxopentanoate (**I41**)

The title compound was prepared according to general procedure A, using (2*S*,4*R*)-1-((*S*)-2-amino-3,3-dimethylbutanoyl)-4-hydroxy-*N*-(4-(4-methylthiazol-5-yl)­benzyl)­pyrrolidine-2-carboxamide (1.00 g, 2.32 mmol), 5-(*tert*-butoxy)-4-oxopentanoic acid (437 mg, 2.32 mmol), HATU (1.15 g, 3.02 mmol) and DIPEA (1.21 mL, 6.97 mmol). The title compound was obtained as a colorless solid (981 mg, 70%). ^1^H NMR (400 MHz, DMSO-*d*_6_) δ: 8.98 (s, 1H), 8.56 (t, *J* = 6.1 Hz, 1H), 7.90 (d, *J* = 9.3 Hz, 1H), 7.51–7.24 (m, 4H), 5.12 (d, *J* = 3.6 Hz, 1H), 4.54 (d, *J* = 9.3 Hz, 1H), 4.48–4.40 (m, 2H), 4.35 (s, 1H), 4.21 (dd, *J* = 15.8, 5.4 Hz, 1H), 3.70–3.53 (m, 2H), 2.44 (s, 3H), 2.32–2.21 (m, 1H), 2.21–2.11 (m, 3H), 2.02 (d, *J* = 8.6 Hz, 1H), 1.94–1.84 (m, 1H), 1.76–1.62 (m, 2H), 1.39 (s, 9H), 0.94 (s, 9H). ESI-MS: *m*/*z* = 623.35 ([M + Na]^+^).

##### Synthesis of 5-(((*S*)-1-((2*S*,4*R*)-4-Hydroxy-2-((4-(4-methylthiazol-5-yl)­benzyl)­carbamoyl)­pyrrolidin-1-yl)-3,3-dimethyl-1-oxobutan-2-yl)­amino)-5-oxopentanoic Acid (**I42**)

The title compound was prepared according to general procedure D, using **I41** (484 mg, 0.806 mmol). The crude product was used without further purification. ESI-MS: *m*/*z* = 545.30 ([M + H]^+^).

##### Synthesis of *N* ^1^-(4-Azidobutyl)-*N* ^5^-((*S*)-1-((2*S*,4*R*)-4-hydroxy-2-((4-(4-methylthiazol-5-yl)­benzyl)­carbamoyl)­pyrrolidin-1-yl)-3,3-dimethyl-1-oxobutan-2-yl)­glutaramide (**X46**)

The title compound was prepared according to general procedure A, using **I42** (143 mg, 0.263 mmol), 4-azidobutan-1-amine (30 mg, 0.26 mmol), HATU (130 mg, 0.341 mmol) and DIPEA (137 μL, 0.788 mmol). The title compound was obtained as a colorless solid (131 mg, 78%). ^1^H NMR (400 MHz, DMSO-*d*_6_) δ: 8.98 (s, 1H), 8.56 (t, *J* = 6.0 Hz, 1H), 7.87 (d, *J* = 9.3 Hz, 1H), 7.77 (t, *J* = 5.7 Hz, 1H), 7.50–7.32 (m, 4H), 5.13 (d, *J* = 3.6 Hz, 1H), 4.53 (d, *J* = 9.3 Hz, 1H), 4.49–4.40 (m, 2H), 4.35 (s, 1H), 4.22 (dd, *J* = 15.9, 5.5 Hz, 1H), 3.73–3.62 (m, 2H), 3.32 (m, 3H), 3.04 (qd, *J* = 6.8, 2.2 Hz, 2H), 2.44 (s, 3H), 2.29–2.10 (m, 2H), 2.07–1.99 (m, 3H), 1.95–1.85 (m, 1H), 1.75–1.63 (m, 2H), 1.56–1.38 (m, 3H), 0.94 (s, 9H). ^13^C NMR (101 MHz, DMSO-*d*_6_) ^13^C NMR (101 MHz, DMSO) δ: 171.9, 171.7, 171.6, 169.7, 151.4, 147.7, 139.5, 131.1, 129.6, 128.6, 127.4, 68.9, 58.7, 56.3, 50.3, 41.6, 37.9, 37.8, 35.1, 34.9, 34.3, 26.3, 25.7, 21.8, 15.9. ESI-MS: *m*/*z* = 641.35 ([M + H]^+^).

##### Synthesis of *N* ^1^-(6-Azidohexyl)-*N* ^5^-((*S*)-1-((2*S*,4*R*)-4-hydroxy-2-((4-(4-methylthiazol-5-yl)­benzyl)­carbamoyl)­pyrrolidin-1-yl)-3,3-dimethyl-1-oxobutan-2-yl)­glutaramide (**X47**)

The title compound was prepared according to general procedure A, using **I42** (143 mg, 0.263 mmol), 6-azidohexan-1-amine (37 mg, 0.26 mmol), HATU (130 mg, 0.341 mmol) and DIPEA (137 μL, 0.788 mmol). The title compound was obtained as a colorless solid (131 mg, 78%). ^1^H NMR (400 MHz, DMSO-*d*_6_) δ: 8.98 (s, 1H), 8.56 (t, *J* = 6.1 Hz, 1H), 7.87 (d, *J* = 9.3 Hz, 1H), 7.72 (t, *J* = 5.6 Hz, 1H), 7.47–7.34 (m, 4H), 5.13 (d, *J* = 3.5 Hz, 1H), 4.53 (d, *J* = 9.3 Hz, 1H), 4.47–4.39 (m, 2H), 4.35 (s, 1H), 4.22 (dd, *J* = 15.9, 5.5 Hz, 1H), 3.72–3.60 (m, 2H), 3.30 (t, *J* = 6.9 Hz, 2H), 3.05–2.96 (m, 2H), 2.44 (s, 3H), 2.28–2.10 (m, 2H), 2.04 (t, *J* = 7.7 Hz, 3H), 1.97–1.86 (m, 1H), 1.74–1.63 (m, 2H), 1.51 (quin, *J* = 6.9 Hz, 2H), 1.38 (quin, *J* = 7.3 Hz, 2H), 1.33–1.23 (m, 4H), 0.94 (s, 9H). ^13^C NMR (101 MHz, DMSO-*d*_6_) δ: 171.9, 171.7, 171.5, 169.7, 151.4, 147.7, 139.5, 131.1, 129.6, 128.6, 127.4, 68.8, 58.7, 56.3, 56.3, 50.5, 41.6, 38.3, 37.9, 35.1, 34.9, 34.3, 29.0, 28.1, 26.3, 25.9, 25.8, 21.8, 15.9. ESI-MS: *m*/*z* = 669.45 ([M + H]^+^).

##### Synthesis of *N* ^1^-(2-(2-(2-(2-Azidoethoxy)­ethoxy)­ethoxy)­ethyl)-*N* ^5^-((*S*)-1-((2*S*,4*R*)-4-hydroxy-2-((4-(4-methylthiazol-5-yl)­benzyl)­carbamoyl)­pyrrolidin-1-yl)-3,3-dimethyl-1-oxobutan-2-yl)­glutaramide (**X48**)

The title compound was prepared according to general procedure B, using **I42** (100 mg, 0.184 mmol) and 1-[2-(2-aminoethoxy)­ethoxy]-2-(2-azidoethoxy)­ethane (60 mg, 0.27 mmol), 1-methyl-1*H*-imidazole (51 μL, 0.64 mmol) and TCFH (61 mg, 0.22 mmol). The title compound was obtained as a colorless solid (128 mg, 94%). ^1^H NMR (500 MHz, CDCl_3_) δ: 8.68 (s, 1H), 7.41–7.31 (m, 5H), 6.82 (t, *J* = 5.6 Hz, 1H), 6.76 (d, *J* = 8.3 Hz, 1H), 4.74 (t, *J* = 8.2 Hz, 1H), 4.59 (dd, *J* = 15.0, 6.7 Hz, 1H), 4.50 (t, *J* = 3.1 Hz, 1H), 4.47 (d, *J* = 8.3 Hz, 1H), 4.33 (dd, *J* = 14.9, 5.1 Hz, 1H), 4.19 (d, *J* = 11.5 Hz, 1H), 3.67–3.61 (m, 12H), 3.59–3.50 (m, 4H), 3.48–3.42 (m, 1H), 3.38 (dd, *J* = 5.5, 4.5 Hz, 3H), 2.60–2.53 (m, 1H), 2.31–2.12 (m, 5H), 2.10–2.02 (m, 1H), 1.97–1.90 (m, 2H), 0.94 (s, 9H). ^13^C NMR (126 MHz, CDCl_3_) δ: 174.1, 172.9, 172.9, 170.6, 150.4, 148.6, 138.2, 131.7, 131.1, 129.6, 128.2, 70.7, 70.5, 70.2, 69.6, 58.5, 58.3, 56.9, 50.8, 43.4, 39.4, 36.0, 34.7, 34.5, 34.4, 29.8, 26.5, 22.2, 16.2. ESI-MS: *m*/*z* = 745.4 ([M + H]^+^).

##### Synthesis of 2-((*S*)-4-(4-Chlorophenyl)-2,3,9-trimethyl-6*H*-thieno­[3,2-*f*]­[1,2,4]­triazolo­[4,3-*a*]­[1,4]­diazepin-6-yl)-*N*-(3-(1-(6-((2-(2,6-dioxopiperidin-3-yl)-1,3-dioxoisoindolin-5-yl)­amino)­hexyl)-1*H*-1,2,3-triazol-4-yl)­propyl)­acetamide (**P1**)

The title compound was prepared according to general procedure E, using **A3** (5.9 mg, 13 μmol), **X15** (5.0 mg, 13 μmol), CuSO_4_·5H_2_O (0.94 mg, 3.8 μmol) and sodium ascorbate (0.75 mg, 3.8 μmol). The title compound was obtained as a yellow solid (9.1 mg, 84%). ^1^H NMR (400 MHz, DMSO-*d*_6_) δ: 11.05 (s, 1H), 8.27 (t, *J* = 5.7 Hz, 1H), 7.86 (s, 1H), 7.55 (d, *J* = 8.3 Hz, 1H), 7.50–7.33 (m, 4H), 7.07 (t, *J* = 5.0 Hz, 1H), 6.93 (s, 1H), 6.82 (dd, *J* = 8.5, 2.1 Hz, 1H), 5.02 (dd, *J* = 12.9, 5.4 Hz, 1H), 4.51 (dd, *J* = 8.4, 5.8 Hz, 1H), 4.30 (t, *J* = 7.0 Hz, 2H), 3.31–3.14 (m, 4H), 3.15–2.96 (m, 3H), 2.96–2.78 (m, 1H), 2.66 (t, *J* = 7.6 Hz, 2H), 2.59 (s, 3H), 2.56–2.53 (m, 1H), 2.39 (s, 3H), 2.03–1.95 (m, 1H), 1.86–1.70 (m, 4H), 1.59 (s, 3H), 1.56–1.48 (m, 2H), 1.44–1.33 (m, 2H), 1.31–1.20 (m, 2H). ^13^C NMR (101 MHz, DMSO-*d*_6_) δ: 172.8, 170.1, 169.5, 167.7, 167.1, 163.0, 154.4, 146.4, 136.7, 135.1, 134.2, 132.2, 130.6, 130.1, 129.8, 129.6, 128.4, 125.1, 121.7, 115.8, 53.9, 49.1, 48.6, 42.3, 38.0, 37.7, 30.9, 29.7, 29.0, 28.1, 25.8, 25.6, 22.5, 22.2, 14.0, 12.6, 11.3. ESI-MS: *m*/*z* = 864.25 ([M + H]^+^).

##### Synthesis of 2-((*S*)-4-(4-Chlorophenyl)-2,3,9-trimethyl-6*H*-thieno­[3,2-*f*]­[1,2,4]­triazolo­[4,3-*a*]­[1,4]­diazepin-6-yl)-*N*-((1-(6-((2-(2,6-dioxopiperidin-3-yl)-1,3-dioxoisoindolin-5-yl)­amino)­hexyl)-1*H*-1,2,3-triazol-4-yl)­methyl)­acetamide (**P2**)

The title compound was prepared according to general procedure E, using **A1** (5.5 mg, 13 μmol), **X15** (5.0 mg, 13 μmol), CuSO_4_·5H_2_O (0.94 mg, 3.8 μmol) and sodium ascorbate (0.75 mg, 3.8 μmol). The title compound was obtained as a yellow solid (10.4 mg, 99%). ^1^H NMR (400 MHz, DMSO-*d*_6_) δ: 1.05 (s, 1H), 8.73 (t, *J* = 5.7 Hz, 1H), 7.94 (s, 1H), 7.55 (d, *J* = 8.4 Hz, 1H), 7.46 (d, *J* = 8.5 Hz, 2H), 7.37 (d, *J* = 8.6 Hz, 2H), 7.07 (t, *J* = 5.3 Hz, 1H), 6.93 (d, *J* = 2.1 Hz, 1H), 6.82 (dd, *J* = 8.3, 1.9 Hz, 1H), 5.02 (dd, *J* = 12.9, 5.4 Hz, 1H), 4.52 (t, *J* = 7.1 Hz, 1H), 4.35 (d, *J* = 5.6 Hz, 2H), 4.31 (t, *J* = 7.1 Hz, 2H), 3.27 (t, *J* = 7.3 Hz, 2H), 3.12 (q, *J* = 6.2 Hz, 2H), 2.94–2.76 (m, 1H), 2.59 (s, 3H), 2.54 (s, 2H), 2.39 (s, 3H), 2.02–1.94 (m, 1H), 1.79 (quin, *J* = 7.4 Hz, 2H), 1.61 (s, 3H), 1.53 (quin, *J* = 6.9 Hz, 2H), 1.46–1.32 (m, 2H), 1.31–1.20 (m, 2H). ^13^C NMR (101 MHz, DMSO-*d*_6_) δ: ^13^C NMR (101 MHz, DMSO) δ: 172.8, 170.1, 169.6, 167.7, 167.1, 163.0, 155.0, 154.4, 149.8, 145.0, 136.7, 135.2, 134.2, 132.2, 130.6, 130.2, 129.8, 129.5, 128.4, 125.1, 122.7, 115.8, 53.9, 49.2, 48.6, 42.3, 40.4, 37.5, 34.2, 30.9, 29.6, 28.0, 25.9, 25.6, 22.2, 14.0, 12.6, 11.3. ESI-MS: *m*/*z* = 836.25 ([M + H]^+^).

##### Synthesis of 2-((*S*)-4-(4-Chlorophenyl)-2,3,9-trimethyl-6*H*-thieno­[3,2-*f*]­[1,2,4]­triazolo­[4,3-*a*]­[1,4]­diazepin-6-yl)-*N*-(3-(1-(6-((2-(2,6-dioxopiperidin-3-yl)-1,3-dioxoisoindolin-4-yl)­amino)­hexyl)-1*H*-1,2,3-triazol-4-yl)­propyl)­acetamide (**P3**)

The title compound was prepared according to general procedure E, using **A3** (5.9 mg, 13 μmol), **X6** (5.0 mg, 13 μmol), CuSO_4_·5H_2_O (0.94 mg, 3.8 μmol) and sodium ascorbate (0.75 mg, 3.8 μmol). The title compound was obtained as a yellow solid (6.2 mg, 57%). ^1^H NMR (400 MHz, DMSO-*d*_6_) δ: 11.08 (s, 1H), 8.26 (t, *J* = 5.7 Hz, 1H), 7.85 (s, 1H), 7.56 (dd, *J* = 8.6, 7.1 Hz, 1H), 7.50–7.36 (m, 4H), 7.06 (d, *J* = 8.6 Hz, 1H), 7.00 (d, *J* = 7.0 Hz, 1H), 6.51 (t, *J* = 5.9 Hz, 1H), 5.04 (dd, *J* = 12.8, 5.4 Hz, 1H), 4.51 (dd, *J* = 8.5, 5.8 Hz, 1H), 4.29 (t, *J* = 7.0 Hz, 2H), 3.29–3.14 (m, 5H), 3.14–3.06 (m, 1H), 2.93–2.77 (m, 1H), 2.66 (t, *J* = 7.7 Hz, 2H), 2.59 (s, 3H), 2.55–2.51 (m, 2H), 2.39 (s, 3H), 2.05–1.98 (m, 1H), 1.84–1.74 (m, 4H), 1.59 (s, 3H), 1.53 (quin, *J* = 7.3 Hz, 2H), 1.35 (quin, *J* = 7.3 Hz, 2H), 1.25 (quin, *J* = 7.2 Hz, 2H). ^13^C NMR (101 MHz, DMSO-*d*_6_) δ: 172.8, 170.1, 169.5, 168.9, 167.3, 163.0, 155.1, 149.8, 146.3, 136.7, 136.2, 135.1, 132.2, 130.6, 130.1, 129.8, 129.5, 128.4, 121.7, 117.1, 110.3, 109.0, 53.9, 49.1, 48.5, 41.7, 38.0, 37.6, 30.9, 29.6, 29.0, 28.4, 25.7, 25.5, 22.5, 22.1, 14.0, 12.6, 11.2. ESI-MS: *m*/*z* = 864.20 ([M + H]^+^).

##### Synthesis of (2*S*,4*R*)-1-((*S*)-2-(4-(4-(2-(2-((*S*)-4-(4-Chlorophenyl)-2,3,9-trimethyl-6*H*-thieno­[3,2-*f*]­[1,2,4]­triazolo­[4,3-*a*]­[1,4]­diazepin-6-yl)­acetamido)­ethyl)-1*H*-1,2,3-triazol-1-yl)­benzamido)-3,3-dimethylbutanoyl)-4-hydroxy-*N*-(4-(4-methylthiazol-5-yl)­benzyl)­pyrrolidine-2-carboxamide (**P4**)

The title compound was prepared according to general procedure E, using **A2** (3.9 mg, 8.7 μmol), **X37** (5.0 mg, 8.7 μmol), CuSO_4_·5H_2_O (0.65 mg, 2.6 μmol) and sodium ascorbate (0.52 mg, 2.6 μmol). The title compound was obtained as a colorless solid (4.5 mg, 50%). ^1^H NMR (400 MHz, DMSO-*d*_6_) δ: 8.98 (s, 1H), 8.79 (s, 1H), 8.58 (t, *J* = 6.1 Hz, 1H), 8.39 (t, *J* = 5.7 Hz, 1H), 8.21 (d, *J* = 9.0 Hz, 1H), 8.09 (d, *J* = 8.8 Hz, 2H), 7.99 (d, *J* = 8.8 Hz, 2H), 7.47–7.35 (m, 7H), 5.16 (d, *J* = 3.6 Hz, 1H), 4.80 (d, *J* = 9.1 Hz, 1H), 4.52 (t, *J* = 7.2 Hz, 1H), 4.46 (q, *J* = 7.2, 6.2 Hz, 1H), 4.43–4.37 (m, 1H), 4.24 (dd, *J* = 15.8, 5.6 Hz, 1H), 3.74 (d, *J* = 3.0 Hz, 2H), 3.52–3.45 (m, 2H), 3.44–3.40 (m, 2H), 3.28–3.23 (m, 2H), 2.91 (t, *J* = 6.9 Hz, 2H), 2.58 (s, 3H), 2.45 (s, 3H), 2.40 (s, 3H), 2.11–2.00 (m, 1H), 1.97–1.90 (m, 1H), 1.60 (s, 3H), 1.05 (s, 9H). ^13^C NMR (101 MHz, DMSO-*d*_6_) δ: 171.9, 169.7, 169.3, 165.5, 163.1, 155.1, 151.4, 149.9, 147.7, 145.9, 139.4, 138.6, 136.7, 135.2, 133.5, 132.2, 131.1, 130.7, 130.1, 129.8, 129.6, 129.5, 129.5, 128.7, 128.4, 127.4, 120.9, 119.0, 68.9, 58.8, 57.4, 56.4, 53.8, 41.6, 39.5, 38.2, 37.9, 37.7, 35.5, 26.5, 25.5, 15.9, 14.0, 12.6, 11.3. ESI-MS: *m*/*z* = 514.30 ([M+2H]^+2^).

##### Synthesis of 2-((*S*)-4-(4-Chlorophenyl)-2,3,9-trimethyl-6*H*-thieno­[3,2-*f*]­[1,2,4]­triazolo­[4,3-*a*]­[1,4]­diazepin-6-yl)-*N*-((1-(6-((2-(2,6-dioxopiperidin-3-yl)-1,3-dioxoisoindolin-4-yl)­amino)­hexyl)-1*H*-1,2,3-triazol-4-yl)­methyl)­acetamide (**P5**)

The title compound was prepared according to general procedure E, using **A1** (5.5 mg, 13 μmol), **X6** (5.0 mg, 13 μmol), CuSO_4_·5H_2_O (0.94 mg, 3.8 μmol) and sodium ascorbate (0.75 mg, 3.8 μmol). The title compound was obtained as a yellow solid (10.1 mg, 96%). ^1^H NMR (400 MHz, DMSO-*d*_6_) δ: 11.08 (s, 1H), 8.72 (t, *J* = 5.8 Hz, 1H), 7.94 (s, 1H), 7.56 (t, *J* = 7.8 Hz, 1H), 7.45 (d, *J* = 8.2 Hz, 2H), 7.37 (d, *J* = 8.2 Hz, 2H), 7.06 (d, *J* = 8.6 Hz, 1H), 7.00 (d, *J* = 7.0 Hz, 1H), 6.51 (t, *J* = 6.0 Hz, 1H), 5.04 (dd, *J* = 12.8, 5.4 Hz, 1H), 4.52 (dd, *J* = 8.1, 6.3 Hz, 1H), 4.35 (d, *J* = 5.7 Hz, 2H), 4.30 (t, *J* = 7.2 Hz, 2H), 3.29–3.22 (m, 4H), 2.94–2.81 (m, 1H), 2.59 (s, 3H), 2.55–2.52 (m, 2H), 2.39 (s, 3H), 2.06–1.98 (m, 1H), 1.78 (quin, *J* = 6.9 Hz, 2H), 1.61 (s, 3H), 1.54 (quin, *J* = 7.2 Hz, 2H), 1.37–1.33 (m, 2H), 1.31–1.23 (m, 2H). ^13^C NMR (101 MHz, DMSO-*d*_6_) δ: 172.8, 170.0, 169.5, 168.9, 167.3, 163.0, 155.0, 149.8, 146.4, 144.9, 136.7, 136.2, 135.2, 132.1, 130.6, 130.2, 129.8, 129.5, 128.4, 122.7, 117.1, 110.3, 109.0, 53.9, 49.2, 48.5, 41.7, 37.5, 34.2, 30.9, 29.6, 28.4, 25.6, 25.5, 22.1, 14.0, 12.6, 11.2. ESI-MS: *m*/*z* = 836.20 ([M + H]^+^).

##### Synthesis of 4-(4-(2-(4-(2-(2-((*S*)-4-(4-Chlorophenyl)-2,3,9-trimethyl-6*H*-thieno­[3,2-*f*]­[1,2,4]­triazolo­[4,3-*a*]­[1,4]­diazepin-6-yl)­acetamido)­ethyl)-1*H*-1,2,3-triazol-1-yl)­acetyl)­piperazin-1-yl)-*N*-(2,6-dioxopiperidin-3-yl)-2-methoxybenzamide (**P6**)

The title compound was prepared according to general procedure E, using **A2** (5.3 mg, 12 μmol), **X35** (5.0 mg, 12 μmol), CuSO_4_·5H_2_O (0.87 mg, 3.5 μmol) and sodium ascorbate (0.69 mg, 3.5 μmol). The title compound was obtained as a colorless solid (5.7 mg, 56%). ^1^H NMR (400 MHz, DMSO-*d*_6_) δ: 10.87 (s, 1H), 8.45 (d, *J* = 7.0 Hz, 1H), 8.36 (t, *J* = 5.5 Hz, 1H), 7.86 (s, 1H), 7.81 (d, *J* = 8.8 Hz, 1H), 7.49 (d, *J* = 8.3 Hz, 2H), 7.42 (d, *J* = 8.2 Hz, 2H), 6.63 (d, *J* = 9.0 Hz, 1H), 6.59 (s, 1H), 5.51 (s, 2H), 4.71 (dt, *J* = 12.5, 6.2 Hz, 1H), 4.52 (t, *J* = 7.1 Hz, 1H), 3.94 (s, 3H), 3.69 (t, *J* = 5.2 Hz, 2H), 3.62 (t, *J* = 5.5 Hz, 2H), 3.45 (s, 2H), 3.41–3.36 (m, 3H), 3.27–3.21 (m, 2H), 2.84 (t, *J* = 7.3 Hz, 2H), 2.76 (ddd, *J* = 18.3, 13.5, 5.6 Hz, 1H), 2.60 (s, 3H), 2.56–2.51 (m, 2H), 2.41 (s, 3H), 2.17–2.01 (m, 2H), 1.63 (s, 3H). ^13^C NMR (101 MHz, DMSO-*d*_6_) δ: 172.9, 172.6, 169.5, 164.5, 164.2, 163.0, 158.9, 155.1, 154.0, 149.8, 144.0, 136.7, 135.2, 132.4, 132.2, 130.7, 130.1, 129.8, 129.5, 128.4, 124.0, 111.0, 106.7, 97.8, 55.9, 53.8, 50.5, 50.0, 46.9, 46.7, 43.7, 41.1, 38.4, 37.6, 31.2, 31.0, 28.3, 25.5, 24.3, 22.0, 14.0, 12.6, 11.3. ESI-MS: *m*/*z* = 881.25 ([M + H]^+^).

##### Synthesis of (2*S*,4*R*)-1-((*S*)-2-(4-(4-((4-(2-((*S*)-4-(4-Chlorophenyl)-2,3,9-trimethyl-6*H*-thieno­[3,2-*f*]­[1,2,4]­triazolo­[4,3-*a*]­[1,4]­diazepin-6-yl)­acetyl)­piperazin-1-yl)­methyl)-1*H*-1,2,3-triazol-1-yl)­benzamido)-3,3-dimethylbutanoyl)-4-hydroxy-*N*-(4-(4-methylthiazol-5-yl)­benzyl)­pyrrolidine-2-carboxamide (**P7**)

The title compound was prepared according to general procedure E, using **A4** (4.4 mg, 8.7 μmol), **X37** (5.0 mg, 8.7 μmol), CuSO_4_·5H_2_O (0.65 mg, 2.6 μmol) and sodium ascorbate (0.52 mg, 2.6 μmol). The title compound was obtained as a colorless solid (5.0 mg, 53%). ^1^H NMR (400 MHz, DMSO-*d*_6_) δ: 8.98 (s, 1H), 8.87 (s, 1H), 8.58 (t, *J* = 6.1 Hz, 1H), 8.25 (d, *J* = 9.0 Hz, 1H), 8.11 (d, *J* = 8.7 Hz, 2H), 8.03 (d, *J* = 8.7 Hz, 2H), 7.48 (d, *J* = 8.7 Hz, 2H), 7.45–7.38 (m, 6H), 5.16 (d, *J* = 3.6 Hz, 1H), 4.81 (d, *J* = 9.1 Hz, 1H), 4.56 (t, *J* = 6.7 Hz, 1H), 4.46 (q, *J* = 7.5, 6.9 Hz, 1H), 4.40 (dd, *J* = 14.4, 5.0 Hz, 2H), 4.24 (dd, *J* = 15.9, 5.6 Hz, 1H), 3.75 (d, *J* = 3.0 Hz, 3H), 3.68 (s, 2H), 3.60 (dd, *J* = 16.4, 7.2 Hz, 1H), 3.55–3.44 (m, 1H), 3.40 (dd, *J* = 16.3, 6.3 Hz, 1H), 2.59 (s, 3H), 2.55–2.51 (m, 2H), 2.48–2.45 (m, 4H), 2.45 (s, 3H), 2.41 (s, 3H), 2.13–2.00 (m, 1H), 1.98–1.89 (m, 1H), 1.62 (s, 3H), 1.06 (s, 9H). ^13^C NMR (101 MHz, DMSO-*d*_6_) δ: 171.9, 169.3, 168.1, 165.5, 162.8, 155.2, 151.4, 149.76 147.7, 139.4, 138.5, 136.7, 135.1, 132.1, 131.1, 130.7, 130.1, 129.8, 129.5, 128.6, 128.4, 127.4, 119.2, 68.9, 58.8, 57.5, 56.4, 54.1, 41.6, 37.9, 35.5, 34.7, 26.6, 15.9, 14.0, 12.6, 11.2. ESI-MS: *m*/*z* = 541.80 ([M+2H]^+2^).

##### Synthesis of (*S*)-2-(4-(4-Chlorophenyl)-2,3,9-trimethyl-6*H*-thieno­[3,2-*f*]­[1,2,4]­triazolo­[4,3-*a*]­[1,4]­diazepin-6-yl)-*N*-(3-(1-(4-(4-(2-(3-(2,4-dioxotetrahydropyrimidin-1­(2*H*)-yl)-2-methylphenoxy)­acetyl)­piperazine-1-carbonyl)­phenyl)-1*H*-1,2,3-triazol-4-yl)­propyl)­acetamide (**P8**)

The title compound was prepared according to general procedure E, using **A3** (4.7 mg, 10 μmol), **X24** (5.0 mg, 10 μmol), CuSO_4_·5H_2_O (0.76 mg, 3.1 μmol) and sodium ascorbate (0.61 mg, 3.1 μmol). The title compound was obtained as a colorless solid (9.3 mg, 95%). ^1^H NMR (400 MHz, DMSO-*d*_6_) δ: 10.32 (s, 1H), 8.65 (s, 1H), 8.32 (t, *J* = 5.7 Hz, 1H), 7.97 (d, *J* = 8.7 Hz, 2H), 7.66 (d, *J* = 8.6 Hz, 2H), 7.48–7.37 (m, 4H), 7.17 (t, *J* = 8.1 Hz, 1H), 6.97–6.78 (m, 2H), 4.91 (s, 2H), 4.52 (dd, *J* = 8.3, 6.0 Hz, 1H), 3.82–3.72 (m, 1H), 3.69–3.38 (m, 8H), 3.32–3.17 (m, 5H), 2.83–2.74 (m, 3H), 2.67 (dt, *J* = 16.6, 5.5 Hz, 1H), 2.59 (s, 3H), 2.40 (s, 3H), 2.05 (s, 3H), 1.88 (quin, *J* = 7.1 Hz, 2H), 1.60 (s, 3H). ^13^C NMR (101 MHz, DMSO-*d*_6_) δ: ^13^C NMR (126 MHz, DMSO) δ: 170.7, 169.6, 168.2, 166.1, 163.0, 156.6, 155.1, 151.7, 149.8, 147.9, 141.7, 137.4, 135.3, 135.2, 132.2, 130.7, 130.1, 129.8, 129.5, 128.8, 128.4, 126.5, 124.2, 120.3, 119.7, 110.7, 66.5, 53.9, 44.6, 40.4, 38.0, 37.7, 31.0, 28.8, 22.5, 14.0, 12.6, 11.3, 10.8. ESI-MS: *m*/*z* = 957.15 ([M + H]^+^).

##### Synthesis of 5-((6-Azidohexyl)­amino)-2-(1-methyl-2,6-dioxopiperidin-3-yl)­isoindoline-1,3-dione (**X15 neg**)

**X15** (20 mg, 50 μmol), cesium carbonate (25 mg, 75 μmol) and iodomethane (3.8 μL, 60 μmol) were dissolved in dry DMF (1 mL) and the reaction mixture was stirred at rt for 18 h. The solution was diluted with saturated aqueous NH_4_Cl (10 mL) and the aqueous phase was extracted with EtOAc (4 × 10 mL). The combined organic phase was dried over MgSO_4_, filtered, and concentrated under reduced pressure. The crude product was purified by flash chromatography using acetonitrile/water as an eluent to obtain the title compound as a yellow solid (19.7 mg, 95%). ^1^H NMR (400 MHz, DMSO-*d*_6_) δ: 7.56 (d, *J* = 8.3 Hz, 1H), 7.10 (t, *J* = 5.3 Hz, 1H), 6.94 (s, 1H), 6.85 (d, *J* = 8.4 Hz, 1H), 5.09 (dd, *J* = 13.2, 5.3 Hz, 1H), 3.35–3.34 (m, 2H), 3.15 (q, *J* = 6.6 Hz, 2H), 3.01 (s, 3H), 2.98–2.87 (m, 1H), 2.74 (d, *J* = 17.0 Hz, 1H), 2.56 (t, *J* = 16.9 Hz, 1H), 2.07–1.96 (m, 1H), 1.63–1.51 (m, 4H), 1.44–1.33 (m, 4H). ^13^C NMR (101 MHz, DMSO-*d*_6_) δ: 171.8, 169.9, 167.7, 167.1, 154.5, 134.2, 125.1, 115.8, 99.2, 50.6, 49.2, 42.4, 31.1, 28.2, 28.1, 26.6, 26.0, 25.9, 21.4. ESI-MS: *m*/*z* = 273.10 ([M + H]^+^).

##### Synthesis of 2-((*S*)-4-(4-Chlorophenyl)-2,3,9-trimethyl-6*H*-thieno­[3,2-*f*]­[1,2,4]­triazolo­[4,3-*a*]­[1,4]­diazepin-6-yl)-*N*-(3-(1-(6-((2-(2,6-dioxopiperidin-3-yl)-1,3-dioxoisoindolin-5-yl)­amino)­hexyl)-1*H*-1,2,3-triazol-4-yl)­propyl)­acetamide (**P1 neg**)

The title compound was prepared according to general procedure E, using **A3** (10 mg, 21 μmol), **X15 neg** (8.8 mg, 21 μmol), CuSO_4_·5H_2_O (1.6 mg, 6.4 μmol) and sodium ascorbate (1.3 mg, 6.4 μmol). The title compound was obtained as a yellow solid (15.7 mg, 83%). ^1^H NMR (400 MHz, DMSO-*d*_6_) δ: 8.27 (t, *J* = 5.6 Hz, 1H), 7.86 (s, 1H), 7.55 (d, *J* = 8.4 Hz, 1H), 7.46–7.37 (m, 4H), 7.08 (t, *J* = 5.3 Hz, 1H), 6.93 (d, *J* = 2.1 Hz, 1H), 6.82 (dd, *J* = 8.4, 2.1 Hz, 1H), 5.09 (dd, *J* = 13.0, 5.4 Hz, 1H), 4.51 (dd, *J* = 8.4, 5.8 Hz, 1H), 4.30 (t, *J* = 7.0 Hz, 2H), 3.30–3.23 (m, 2H), 3.22–3.17 (m, 2H), 3.16–3.06 (m, 3H), 3.00 (s, 3H), 2.99–2.87 (m, 1H), 2.74 (dt, *J* = 17.2, 3.7 Hz, 1H), 2.66 (t, *J* = 7.7 Hz, 2H), 2.59 (s, 3H), 2.39 (s, 3H), 2.05–1.95 (m, 1H), 1.86–1.71 (m, 4H), 1.59 (s, 3H), 1.54 (quin, *J* = 14.4, 7.2 Hz, 2H), 1.37 (quin, *J* = 7.1 Hz, 2H), 1.25 (quin, *J* = 14.1, 6.9 Hz, 2H). ^13^C NMR (101 MHz, DMSO-*d*_6_) ^13^C NMR (101 MHz, DMSO-*d*_6_) δ: 171.9, 170.0, 169.6, 167.7, 167.2, 163.1, 155.1, 154.5, 149.9, 146.4, 136.8, 135.2, 134.2, 132.3, 130.7, 130.1, 129.8, 128.5, 125.1, 121.8, 115.8, 53.9, 49.2, 49.1, 42.4, 38.1, 37.7, 31.2, 29.7, 29.1, 28.0, 26.6, 25.9, 25.6, 22.6, 21.5. ESI-MS: *m*/*z* = 878.30 ([M + H]^+^).

### HiBiT End Point Detection for BRD4 Degradation

Endogenously BRD4 HiBiT-tagged HEK293T (HEK293T^BRD4‑HiBiT^) cells were obtained as a kind gift from Promega Corp. To measure degradation, 10 μL of a total concentration of 2.5 × 10^5^ cells/ml in DMEM medium were seeded into white small volume 384 well plates (Greiner, 784 075) and allowed to settle overnight. Subsequently, the PROTACs were added to the seeded cells, using an Echo acoustic dispenser (Labcyte) and the plate was incubated for the indicated time at 37 °C and 5% CO_2_. After incubation, HiBiT Lytic detection reagent was prepared by dilution of LgBiT protein (1:100) and lytic substrate (1:50) in Lytic detection buffer (Promega, N3040). For detection, 10 μL of the prepared mix was added to the treated cells and incubated for 10 min at rt. Readout was carried out in a PHERAStar FSX plate reader (BMG Labtech) using the LUM plus optical module. Degradation data were then plotted with GraphPad Prism 9 software using a normalized 3-parameter curve fit with the following equation: *Y* = 100/(1 + 10̂(*X* – LogIC_50_))

### HiBiT End Point Detection for WDR5 and AurkA Degradation

MV4-11^AURORA‑A‑HiBiT^ or MV4-11^WDR5‑HiBiT^ cells were seeded in a black 96-well cell culture microplate (Greiner) and treated for 6 h with the test compounds at concentrations of 50 nM, 200 nM or 1 μM. JB301 and AD122 were used at concentrations of 150 nM and 1 μM, respectively. The control cells were treated with dimethyl sulfoxide (DMSO; Roth). The Nano-Glo HiBiT Lytic Detection System (Promega) was used for the assay. The luminescence was measured using the Infinite M Plex, multimode microplate reader (TECAN).

### Nanoluciferase Live Cell Measurement for WDR5 and AurkA Degradation

MV4-11^AURORA‑A‑Nluc(Kless)^ or MV4-11^WDR5‑Nluc(Kless)^ cells were seeded in a black 96-well cell culture microplate (Greiner) at a density of 200 000 cells/mL in Opti-MEM reduced serum medium, without phenol red (Thermo Fisher Scientific), supplemented with 10% FBS (Capricorn Scientific), 15 mM HEPES pH 7.2. The Nano-Glo Endurazine live cell substrate (Promega) was diluted and added to the cells according to the manufactureŕs instructions. Prior to the addition of the test compounds, the cells were incubated for 3 h at 37 °C in 5% CO_2_. Finally, the cells were treated with 1 μM of the PROTACs and the kinetic measurement was done at 15 min intervals during 12 h at 37 °C. JB301 was used at a concentration of 150 nM and AD122 at 1 μM. DMSO served as a vehicle control. The luminescence was measured using the Infinite M Plex, multimode microplate reader (TECAN).

### HiBiT End Point Detection for sEH Degradation

The degradation of sEH induced by PROTACs was evaluated using the previously established sEH-HiBiT lytic assay, following the published protocol.^25^ Briefly, HeLa cells stably expressing the sEH-HiBiT fusion protein (hereafter referred to as HeLa^sEH HiBiT^) were cultured in growth medium DMEMsup. Cells were maintained at 37 °C in a 5% CO_2_ atmosphere. For assay setup, cells were harvested in growth medium, adjusted to a density of 4 × 10^5^ cells/mL, and seeded into 384 well tissue culture plates at 50 μL per well (equivalent to 2000 cells per well) using a Multidrop Combi dispenser (Thermo Fisher Scientific). Plates were sealed with AeraSeal semipermeable film and incubated for 24 h at 37 °C, 5% CO_2_. For compound screening, crude PROTACs were tested at final concentrations of 200 nM and 1 μM. Stock solutions were prepared in DMSO at 40 μM and 200 μM in a 96-deep-well plate, then diluted with growth medium to 2.2 μM and 11 μM (final DMSO concentration 5.5%). From these dilutions, 5 μL was added to each well in triplicate, yielding a final assay volume of 55 μL and a final DMSO concentration of 0.5%. Plates were centrifuged for 1 min at 300 rpm, resealed, and incubated for 6 or 18 h at 37 °C and 5% CO_2_. Following treatment, cells were washed four times with DPBS using a HydroSpeed plate washer (Tecan), leaving 10 μL of residual volume in each well. Cell lysis was carried out by adding 1 μL of Mammalian Lysis Buffer, followed by centrifugation for 1 min at 300 rpm and a 10 min incubation at rt. In the meantime, the Nano-Glo substrate mix was freshly prepared using 6760 μL of Nano-Glo HiBiT Extracellular Buffer, 135 μL of Nano-Glo HiBiT Extracellular Substrate, and 68 μL of LgBiT Protein (all from the Nano-Glo HiBiT Extracellular Detection System Kit, Promega, calculated for 616 wells). After lysis, 10 μL of the substrate mix was added to each well, followed by centrifugation (1 min at 300 rpm) and a 10 min incubation at rt. Luminescence was then measured using a Spark Multimode Microplate Reader (Tecan). The average luminescence of each triplicate was normalized to the DMSO control and plotted against the respective azide using Prism 7.0 (GraphPad Software).

## Supplementary Material





## Notes

Supplementary Figure S1 (optimized reaction conditions and used devices), Supplementary Figure S2 (conversion of the CLICK reaction of CuAAC chemistry for sEH, WDR5 and AurkA), Supplementary Figure S3 (Fit of the NanoLuc time dependent live cell measurement monitoring the degradation of AurkA), Supplementary Figure S4 (NanoLuc time dependent live cell measurement monitoring the degradation of AurkA), Supplementary Figure S5 (Cell viability of HEK293T cells after 48 h), Supplementary Figure S6 (cell viability test of crude BRD4 PROTACs), Supplementary Figure S7 (Cell viability of crude AurkA PROTACs), Supplementary Figure S8 (Dose response curves of P1 and its negative control), Supplementary Figure S9 (Rescue experiments for various BRD4 PROTACs), Supplementary Figure S10 (Cell viability test of the purified BRD4 PROTACs), Supplementary Figure S11 (Solubility limit assay data of the purified BRD4 PROTACs), Supplementary Figure S12 (Metabolic stability data of the purified BRD4 PROTACs) and Supplementary Table S1 with a more detailed description of the targeted proteins and cell handling, as well as NMR spectra and LC–MS spectra of selected purified PROTACs (PDF)
Molecular formula strings and data of the purified PROTACs (CSV)

## Abbreviations

A: alkyne
AurkA: Aurora kinase A
BA: benzamide
BRD4: Bromodomain-containing protein 4
CuAAC: azide–alkyne cycloaddition
D2B: direct-to-biology
HTS: high-throughput synthesis
PDHU: dihydrouracil
POI: protein of interest
PROTACs: Proteolysis Targeting Chimeras
sEH: soluble epoxide hydrolase
Thal: thalidomide
TPD: targeted protein degradation
WDR5: WD repeat-containing protein 5
