# Supplementary material for: Click. Screen. Degrade. A Miniaturized D2B Workflow for Rapid PROTAC Discovery
Source: J Med Chem. 2026 Jan 23;69(3):2599–624. doi: 10.1021/acs.jmedchem.5c02543 (PMC12910640; doi:10.1021/acs.jmedchem.5c02543)
Supplement: Supplementary file 1 [file jm5c02543_si_001.pdf]

Click. Screen. Degrade. A Miniaturized D2B Workflow for rapid PROTAC Discovery

Marko Mitrović[1,2,ǂ], Francesco Aleksy Greco[1,2,3,ǂ], Yiliam Cruz García[4], Aleksandar Lučić[1,2], Lasse Hoffmann[1,2], Rohit Chander[1], Julia Schönfeld[1], Nick Liebisch[1], Saran Aswathaman Sivashanmugam[1,2], Martin Peter Schwalm[1,2,3], Markus Egner[5], Max Lewandowski[5], Daniel Merk[5], Viktoria Morasch[1,2], Elmar Wolf[4], Susanne Müller[1,2], Thomas Hanke[1,2], Ewgenij Proschak[1,6], Kerstin Hiesinger[1,*], and Stefan Knapp[1,2,3,*]

[1] Institute of Pharmaceutical Chemistry, Goethe University Frankfurt, Max-von-Laue-Str. 9, 60438 Frankfurt am Main, Germany

[2] Structural Genomics Consortium (SGC), Buchmann Institute for Molecular Life Sciences (BMLS), Max-von-Laue-Str. 15, 60438 Frankfurt am Main, Germany

[3] German Cancer Research Center (DKFZ), Im Neuenheimer Feld 280, 69120 Heidelberg, Germany

[4] Institute of Biochemistry, University of Kiel, Rudolf-Höber-Str. 1, 24118 Kiel, Germany

[5] Department of Pharmacy, Ludwig-Maximilians-Universität (LMU) München, 81377 Munich, Germany

[6] Fraunhofer Institute for Translational Medicine and Pharmacology (ITMP), Theodor-Stern-Kai 7, 60596 Frankfurt/Main, Germany

ǂ These authors contributed equally

*Correspondence: hiesinger@pharmchem.uni-frankfurt.de, knapp@pharmchem.uni-frankfurt.de

Supplementary Information

Supplementary Figures and Tables 3

Supplementary Figure S1. (Related to Figure 4A). Reaction scheme, conditions and setup for the synthesis of 192 unique BRD4 PROTACs. 3

Supplementary Figure S2. (Related to Figure 5C). Color-coded heat-map indicating overall conversion rates (in %) of CuAAC chemistry of sEH ligands A5-8, WDR5 ligands A9-12 and AurkA ligands A13-16 with various selected azides 4

Supplementary Figure S3. (Related to Figure 6E) NanoLuc time dependent live cell measurement monitoring the degradation of AurkA. 5

Supplementary Figure S4. (Related to Figure 6E). NanoLuc time dependent live cell measurement monitoring the degradation of AurkA. 6

Supplementary Figure S5. Cell viability of HEK293T after 48 h. 7

Supplementary Figure S6. Cell viability test of various crude BRD4 PROTACs after 24 h. 7

Supplementary Figure S7. Cell viability test of the crude AurkA PROTACs after 24 h. 8

Supplementary Figure S8. Dose response curves for the purified PROTAC P1 and the corresponding negative control in the HEK293T$^{BRD4\text{-}HiBiT}$ cells. 8

Supplementary Figure S9. Rescue experiments of the various resynthesized PROTACs in the HEK293T$^{BRD4\text{-}HiBiT}$ cells. 9

Supplementary Figure S10. Cell viability test of the purified BRD4 PROTACs after 24 h. 10

Supplementary Figure S11. Solubility limit assay data of the purified BRD4 PROTACS. 10

Supplementary Figure S12. Metabolic stability data of the purified BRD4 PROTACS. 11

Supplementary Table S1. Various properties of target proteins BRD4, sEH, WDR5 and AurkA selected for this study. 12

Cell Systems and Cell Handling..13

Cell lines 15

Cells for WDR5 and AurkA degradation 15

Cells for sEH degradation 16

Cell Viability Assay 18

Solubility Limit Assay 18

Microsomal Stability assay in Liver Microsomes 18

NMR Spectra and LC-MS Spectra, related to compounds in Figure 4G 20

Supplementary Figures and Tables

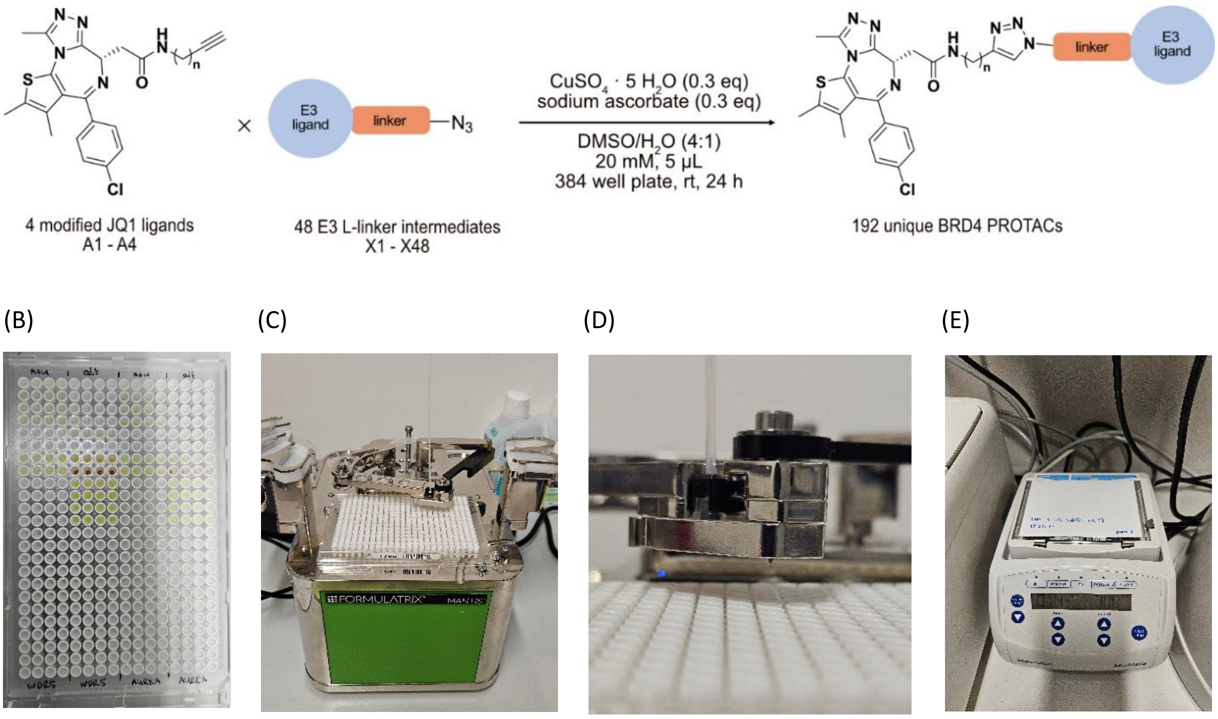

Supplementary Figure S1. (Related to Figure 4A). Reaction scheme, conditions and setup for the synthesis of 192 unique BRD4 PROTACs.

(A) Optimized reaction conditions for the CuAAC chemistry using modified BRD4-targeting ligands A1-A4 and azides X1-48 to obtain 192 unique BRD4 PROTACs. The equivalent of alkyne and azide was set to 1 eq., the equivalent of $CuSO_4 \cdot 5H_2O$ and sodium ascorbate to 0.3 eq., with the reaction temperature at rt and the reaction time at 24 h (plate shaker: 300 rpm). (B) 384 well plate with pipetted azide stock solutions. (C) MANTIS® Liquid Dispenser in action (zoomed out). (D) MANTIS® Liquid Dispenser in action (zoomed in). (E) Plate shaker with attached sealed 384 well plate at 300 rpm.

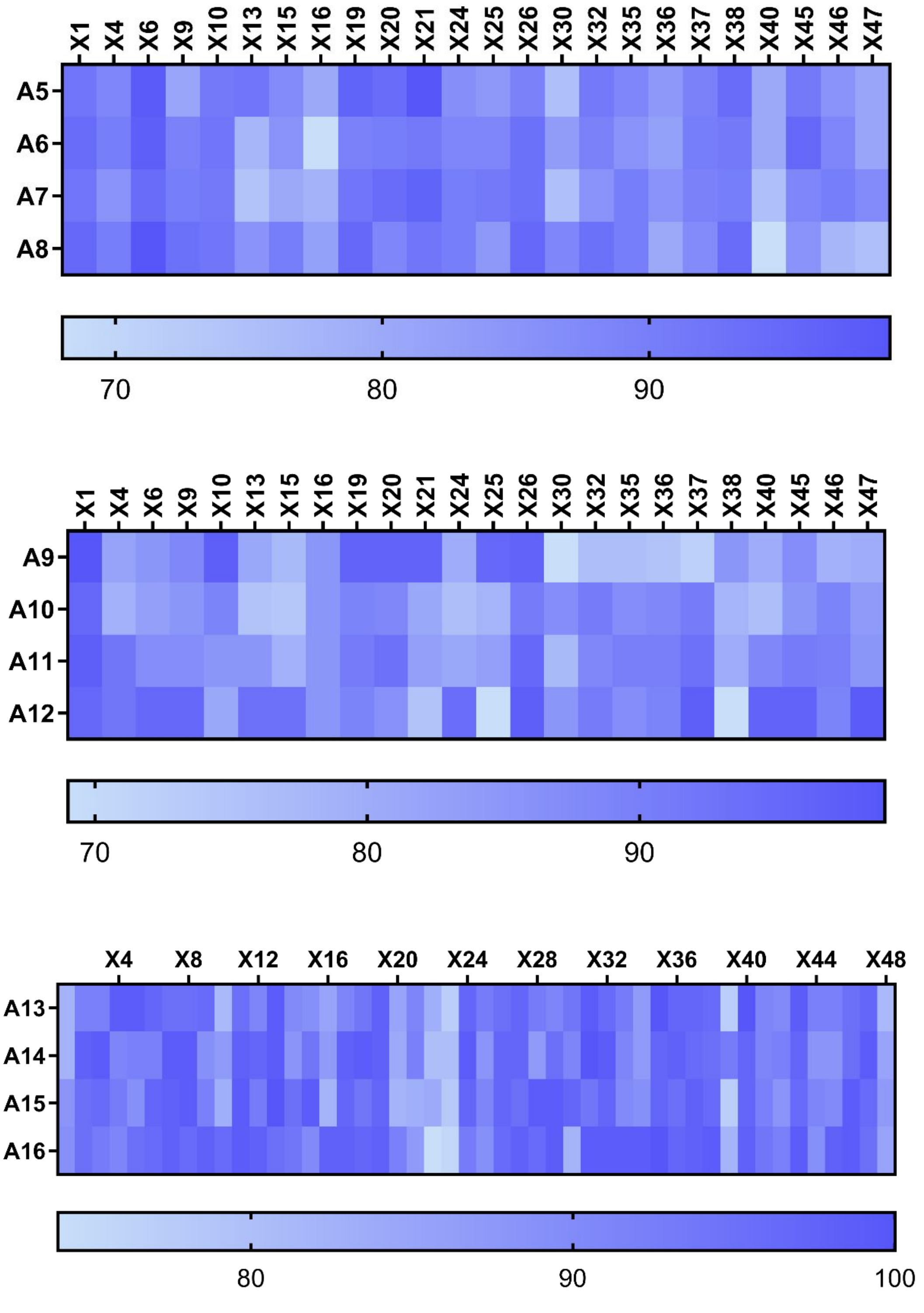

Supplementary Figure S2. (Related to Figure 5C). Color-coded heat-map indicating overall conversion rates (in %) of CuAAC chemistry of sEH ligands A5-8, WDR5 ligands A9-12 and AurkA ligands A13-16 with various selected azides.

Conversion of the CuAAC chemistry for the combination of ligands A5-16 and various selected azides based on HPLC UV data. The average conversion rate for the combination of WDR5-targeting ligand A12 with the respective azides amounts to 90.3%.

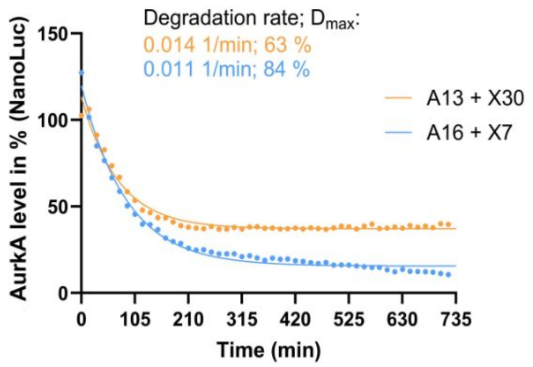

One phase decay	A16 + X7	A13 + X30
Y0	119,9	113,3
Plateau	15,63	37,18
K	0,01136	0,01390
Half Life	61,03	49,87
Tau	88,05	71,94
Span	104,3	76,16

Supplementary Figure S3. (Related to Figure 6E) NanoLuc time dependent live cell measurement monitoring the degradation of AurkA.

Treatment of MV4-11$^{WDR5\text{-}Nluc(Kless)}$ cells with crude reaction mixtures of PROTACs A16 + X7 and A13 + X30 at a PROTAC concentration of 1 µM. The kinetic measurement was done at 15 min intervals during 12 h at 37 °C. The initial phase of each concentration-dependent degradation curve was fitted using a one-component exponential decay model in GraphPad Prism. From this analysis, the best fit parameters K (degradation rate) and plateau (minimum remaining fraction) were determined.

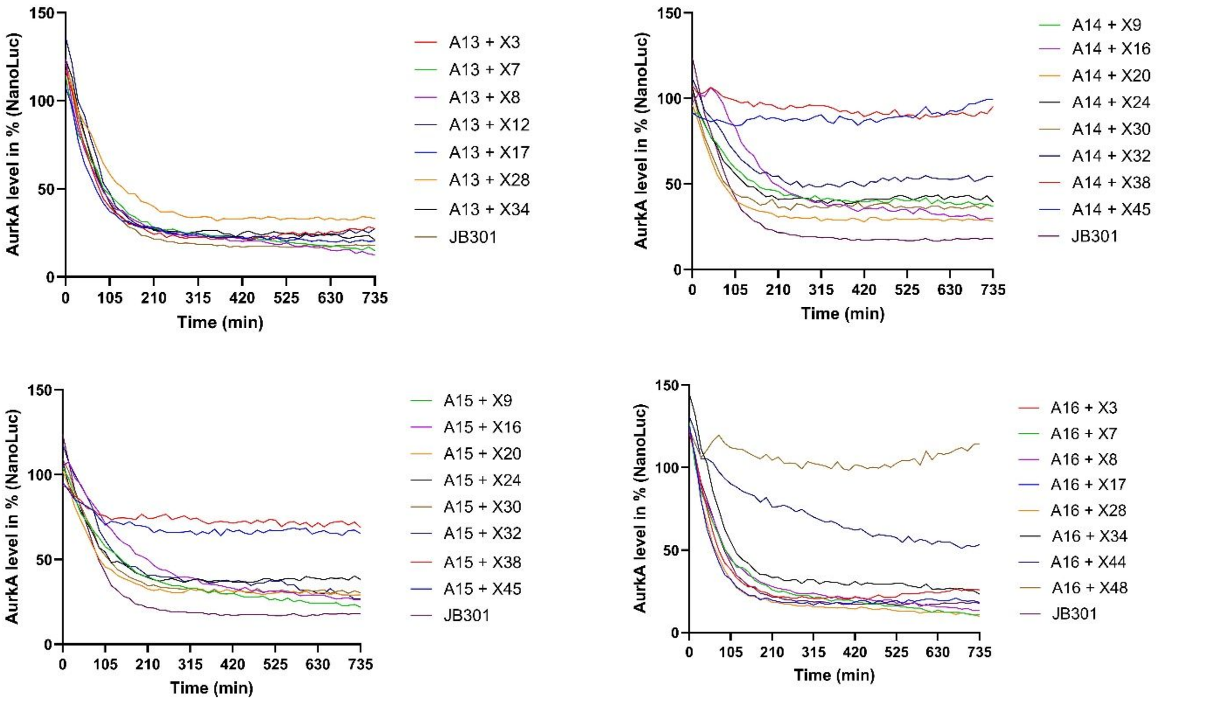

Supplementary Figure S4. (Related to Figure 6E). NanoLuc time dependent live cell measurement monitoring the degradation of AurkA.

Treatment of MV4-11$^{WDR5\text{-}Nluc(Kless)}$ cells with crude reaction mixtures of different PROTACs at a PROTAC concentration of 1 µM. The kinetic measurement was done at 15 min intervals during 12 h at 37 °C. JB301 was used a positive control at a concentration of 150 nM.

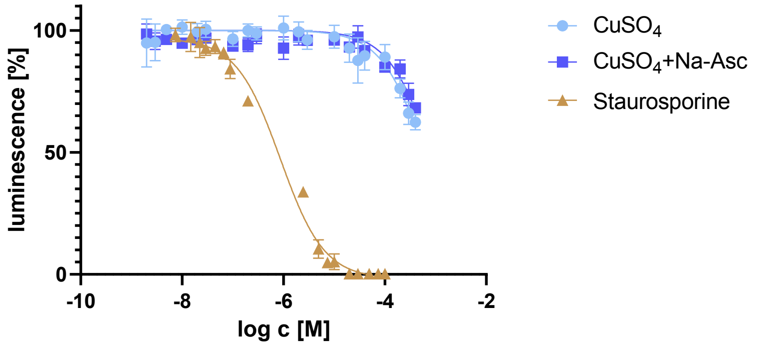

Supplementary Figure S5. Cell viability of HEK293T after 48 h.

HEK293T cells were treated with a dilution series of the additives of the CuAAC reaction. After 48 h the CellTiter-Glo® 2.0 kit was added and the luminesce was measured. Staurosporine served as death control.

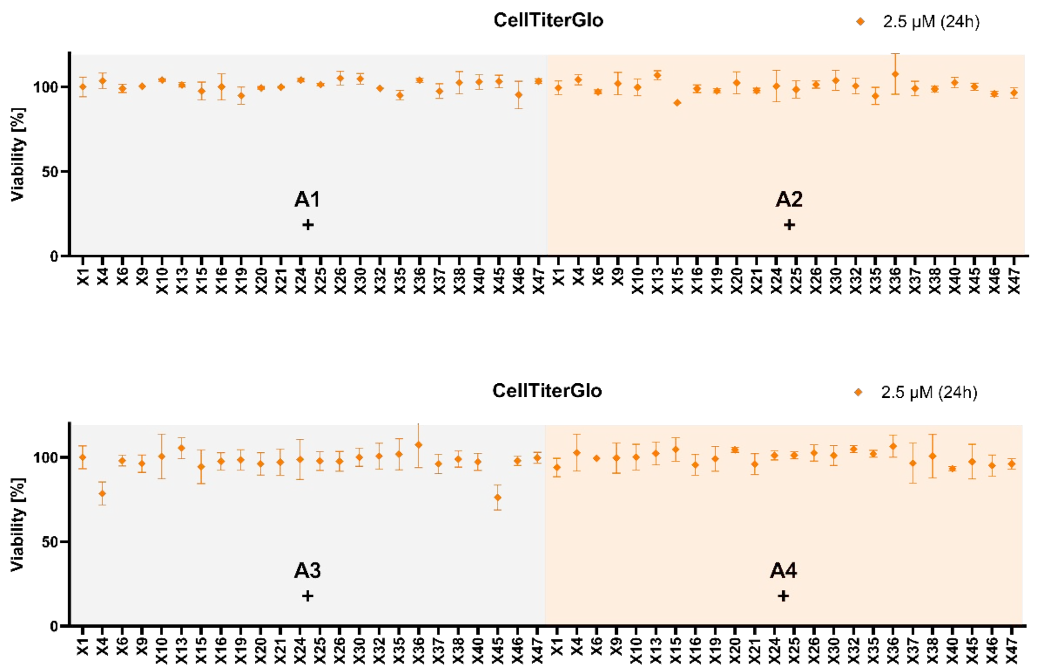

Supplementary Figure S6. Cell viability test of various crude BRD4 PROTACs after 24 h.

HEK293T$^{BRD4\text{-}HiBiT}$ cells were treated with the various BRD4 PROTACs (final concentration 2.5 µM). After 24 h the CellTiter-Glo® 2.0 kit was added and the luminesce was measured.

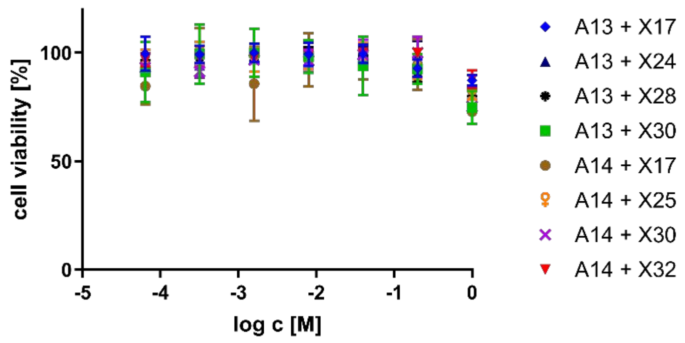

Supplementary Figure S7. Cell viability test of the crude AurkA PROTACs after 24 h.

MV4-11$^{AURORA\text{-}A\text{-}HiBiT}$ cells were treated with a dilution series of the crude AurkA PROTACs. After 24 h the cells were incubated with the alamarBlue™ reagent and the fluorescence was detected.

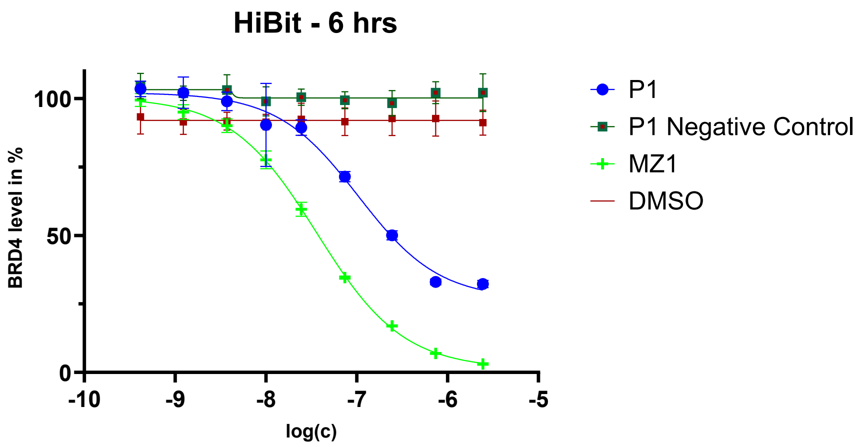

Supplementary Figure S8. Dose response curves for the purified PROTAC P1 and the corresponding negative control in the HEK293T$^{BRD4\text{-}HiBiT}$ cells.

HEK293T$^{BRD4\text{-}HiBiT}$ cells were treated with a dilution series of each PROTAC for 6 hours. No BRD4 degradation was observed for the negative control, confirming that the degradation is E3 ligase dependent.

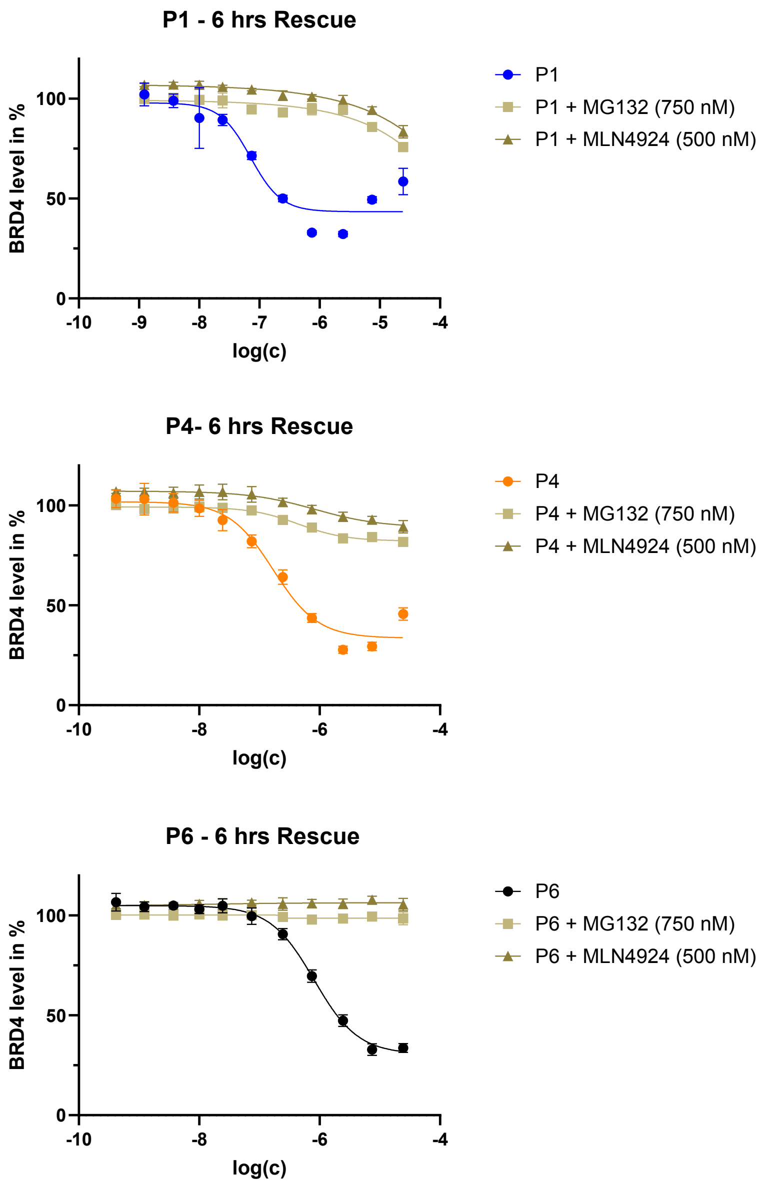

Supplementary Figure S9. Rescue experiments of the various resynthesized PROTACs in the HEK293T$^{BRD4\text{-}HiBiT}$ cells.

HEK293T$^{BRD4\text{-}HiBiT}$ cells were treated with a dilution series of each PROTAC, both in the presence and absence of the controls MG132 (750 nM) and MLN4924 (500 nM). No BRD4 degradation was observed in the presence of these controls, confirming that the degradation is proteasome-dependent.

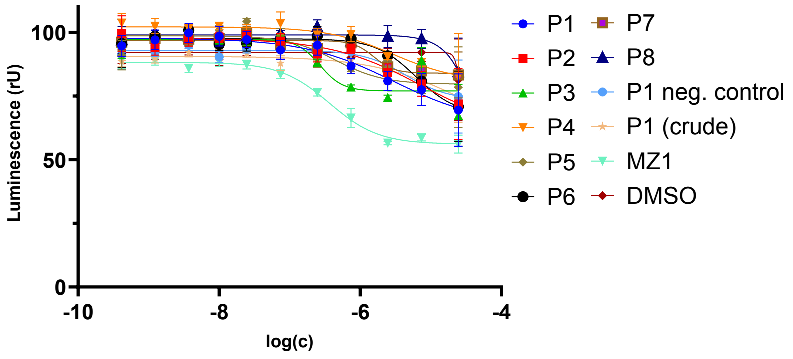

Supplementary Figure S10. Cell viability test of the purified BRD4 PROTACs after 24 h.

HEK293T$^{BRD4\text{-}HiBiT}$ cells were treated with a dilution series of each PROTAC. After 24 h the CellTiter-Glo® 2.0 kit was added and the luminesce was measured.

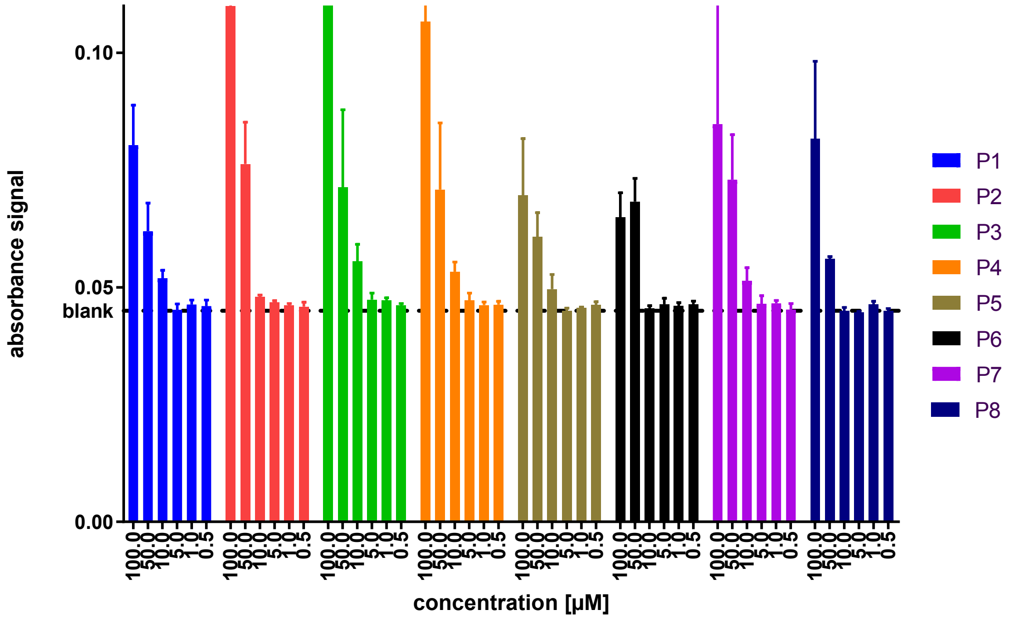

Supplementary Figure S11. Solubility limit assay data of the purified BRD4 PROTACS.

A dilution series of each PROTAC was diluted with PBS buffer and the absorbance was detected at 600 nm.

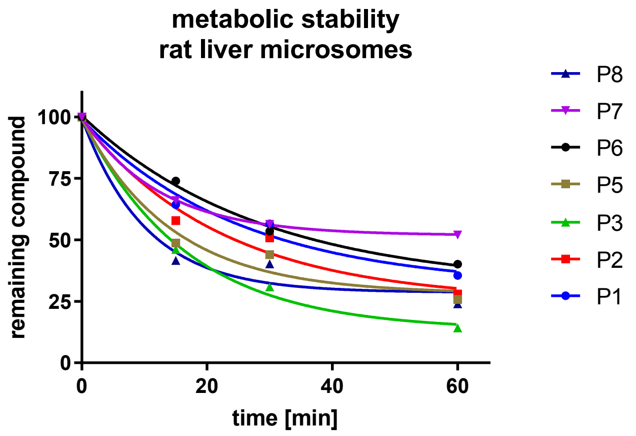

Supplementary Figure S12. Metabolic stability data of the purified BRD4 PROTACS.

Rat liver microsomes were incubated with each PROTAC and the remaining compound was detected by HPLC UV detection.

POI	Mass (Da)	protein family	function	subcellular localization
BRD4	152,219	bromodomain and extra terminal domain family	Chromatin reader protein; key role in transmission of epigenetic memory	Nucleoplasm
sEH	62,616	Epoxide hydrolase	Mainly: metabolism of lipid mediators	Cytosol, peroxisome
WDR5	36,588	WD-repeat proteins	Contributes to histone modification	Nucleoplasm
AurkA	45,823	Serine/threonine-protein kinase	Regulation of cell cycle progression	Mainly: centrosome and basal body

Supplementary Table S1. Various properties of target proteins BRD4, sEH, WDR5 and AurkA selected for this study.

Cell Systems and Cell Handling

Reagents	Supplier	Cat. No.
DMEM (1X) medium with phenol red	Thermo Fisher Scientific	Cat #41965-039
Corning® Fetal Bovine Serum	Corning	Cat# 35-079-CV
Penicillin-Streptomycin (10.000 U/ml)	Gibco	Cat#15140122
Natriumpyruvat (100 mM)	Gibco	Cat# 11360070
NuncTM white polystyrole, flat bottom	Thermo Fisher Scientific	Cat# 164610
semipermeable AeraSeal™ film	Sigma-Aldrich	Cat# A9224
96-Deepwell plate	nerbe plus	Cat# 04-072-0500
Mammalian Lysis Buffer	Promega	Cat# G9381
DMEM (1X) medium	Thermo Fisher Scientific (Gibco)	Cat#41966-029
RPMI 1640 medium	Thermo Fisher Scientific (Gibco)	Cat# 21875-091
Opti-MEM™ Reduced Serum Medium, no phenol red	Thermo Fisher Scientific (Gibco)	Cat#11058-021
Fetal Bovine Serum	Capricorn Scientific	Cat#FBS-11A
Penicillin-Streptomycin (10.000 U/ml)	Sigma-Aldrich	Cat#P4333-100mL
Polyethylenimine (PEI)	Sigma-Aldrich	Cat#408727
Puromycin	InvivoGen	Cat#ant-pr-1
Phusion High-Fidelity DNA Polymerase	Thermo Fisher Scientific	Cat#F530L
MluI-HF	New England Biolabs	Cat#R3198S
AgeI-HF	New England Biolabs	Cat#R3552L
BamHI-HF	New England Biolabs	Cat#R3136S
JB301	Bozilovic et al.[24]	N/A
AD122	Dölle et al.[21]	N/A
**Assay Kits**		

Nano-Glo® HiBiT Extracellular Detection System	Promega	Cat#N2420
Nano-Glo® HiBiT Lytic Detection System	Promega	Cat#N3040
Nano-Glo® Endurazine™ Live Cell Substrate	Promega	Cat#N2571
Advanced TC- 96 Well Cell Culture Microplate, black	Greiner	Cat#655986
white 384-well LDV plates	Greiner	Cat#784075
**Used oligonucleotides**		
Nluc(kless)_f (forward primer) TACGCGTCATATGACTAGTGGGA	This paper	N/A
Nluc(kless)_r (reverse primer) CGGATCCTCACGCCAGAATGCGTTCGCA	This paper	N/A
AURORA-A_f (forward primer) CCACCGGTATGGACCGATCT	This paper	N/A
AURORA-A_r (reverse primer) GTACGCGTAGACTGTTTGCTAGCTGATTCTTTGTTTTG	This paper	N/A
WDR5_f (forward primer) CCACCGGTATGGCGACGG	This paper	N/A
WDR5_r (reverse primer) GTACGCGTGCAGTCACTCTTCCACAGTTTAATTGTT	This paper	N/A
**Recombinant DNA**		
psPAX2 (plasmid)	D. Trono	Addgene # 12260
pMD2.G (plasmid)	D. Trono	Addgene # 12259
pRRL-PGK-Puro (plasmid)	E. Wolf	N/A
pRRL-PGK-Hygro-HiBiT-AURORA-A (plasmid)	Adhikari et al.[20]	N/A
pRRL-PGK-Hygro-HiBiT-WDR5 (plasmid)	Dölle et al.[21]	N/A
pRRL-Puro-C-term-Luc-FKBP12(Kless) (plasmid)	Adhikari et al.[20]	N/A
pRRL-PGK-Puro-Nluc(Kless) (plasmid)	This paper	N/A
pRRL-PGK-Puro-AURORA-A-Nluc(Kless) (plasmid)	This paper	N/A
pRRL-PGK-Puro-WDR5-Nluc(Kless) (plasmid)	This paper	N/A
**Software**		

GraphPad Prism 7, 9 + 10	GraphPad Software	https://www.graphpad.com/
BioRender for the TOC	BioRender	https://www.biorender.com/

Cell lines

HEK293 (female, fetus) cells were regularly tested for mycoplasma infection. Cells were grown in DMEM medium supplemented with 10% fetal bovine serum (FBS) and 1% Penicillin/Streptomycin (100 U/ml penicillin and 100 mg/ml streptomycin) at 37 °C and 5% CO_2. Human MV4-11 (male, acute monocytic leukemia) cells were regularly tested for mycoplasma infection. Cells were grown in RPMI-1640 medium (Thermo Fisher Scientific) supplemented with 10% FBS and 1% Penicillin/Streptomycin (100 U/ml penicillin and 100 mg/ml streptomycin) at 37 °C in 5% CO_2. HeLa cells were stably transfected with sEH-HiBiT fusion protein and regularly tested for mycoplasma infection. Cells were grown in DMEM medium supplemented with 10% FBS, 1% Penicillin/Streptomycin (100 U/ml penicillin and 100 mg/ml streptomycin) and 1 mM sodium pyruvate at 37 °C and 5% CO_2.

Cells for WDR5 and AurkA degradation

The mutated nanoluciferase (K55R, K77R, K80R, K91R, K125R, K126R and K138R), referred to as Nluc (Kless), was cloned by PCR amplification of the vector pRRL-Puro-C-term-Luc-FKBP12 (Kless) using the forward primer TACGCGTCATATGACTAGTGGGA and the reverse primer CGGATCCTCACGCCAGAATGCGTTCGCA. The amplified product was inserted into the pRRL-PGK-Puro entry vector using MluI/BamHI restriction sites to yield pRRL-PGK-Puro-Nluc (Kless). AURORA-A-Nluc (Kless) was cloned by amplification of the vector pRRL-PGK-Hygro-HiBiT-AURORA-A, containing full-length AURORA-A, via PCR using the forward and reverse primers CCACCGGTATGGACCGATCT and GTACGCGTAGACTGTTTGCTAGCTGATTCTTTGTTTTG, respectively. pRRL-PGK-Puro-AURORA-A-Nluc (Kless) was generated through insertion of the PCR product into the pRRL-PGK-Puro-Nluc (Kless) entry vector using AgeI/MluI restriction sites. WDR5-Nluc (Kless) was cloned by PCR amplification of the vector pRRL-PGK-Hygro-HiBiT-WDR5 containing full-length WDR5 using the forward primer CCACCGGTATGGCGACGG and the reverse primer GTACGCGTGCAGTCACTCTTCCACAGTTTAATTGTT. The PCR product was inserted into the pRRL-PGK-Puro-Nluc (Kless) entry vector using AgeI/MluI restriction sites to obtain pRRL-PGK-Puro-WDR5 Nluc (Kless). The PCR reactions were performed using the Phusion High-Fidelity DNA Polymerase (Thermo Fisher Scientific).

Stable MV4-11$^{\text{AURORA-A-NLuc(Kless)}}$ and MV4-11$^{\text{WDR5-NLuc(Kless)}}$ cells were generated using lentiviral infection. The lentivirus was produced by transfecting HEK293T cells with the plasmids psPAX2, pMD2.G, and

pRRL-PGK-Puro-AURORA-A-Nluc (Kless) or the pRRL-PGK-Puro-WDR5-Nluc (Kless) using polyethyleneimine (PEI, Sigma). The virus-containing supernatant was filtered and used to infect MV4-11 cells, which were subsequently selected with puromycin (InvivoGen) at a final concentration of 2 µg/mL after 48 h of infection.

Cells for sEH degradation

To generate the HeLa-sEH-HiBiT cell line, HeLa cells were stably transfected with the construct hsEH_aa1-aa555_Linker-HiBiT_pSB-hPGK using the Sleeping Beauty transposon system (10.1002/biot.201400821). This construct, assembled via Gibson cloning, encodes the human soluble epoxide hydrolase (sEH; amino acids 1–555) fused at its C-terminus to the HiBiT peptide, driven by the human PGK promoter. Initially, an intermediate construct, hsEH_aa1-aa555_Linker-HiBiT_pSBtet, was created by inserting the hsEH_aa1-aa555_Linker-HiBiT sequence into the pSBtet-bla vector. This version enables stable expression of the fusion protein under the control of a doxycycline-inducible tetOn promoter. Subsequently, the tetOn promoter was replaced with the constitutive hPGK promoter to produce the final construct, hsEH_aa1-aa555_Linker-HiBiT_pSB-hPGK. Primers for DNA amplification were obtained from Eurofins. All PCR reactions were carried out using Q5® High-Fidelity DNA Polymerase following the manufacturer's instructions. Each PCR product underwent digestion with DpnI at 37 °C for 1 h, followed by enzyme inactivation at 80 °C for 20 min, and subsequent purification using the GeneJET PCR Purification Kit according to the supplier's protocol. The hsEH-Linker-HiBiT insert was generated through a two-step process. In PCR 1, a dsDNA fragment encoding the Linker-HiBiT sequence was amplified from a plasmid containing the codon-optimized CDS for the linker (SSGNSGGSSG) and HiBiT (VSGWRLFKKIS) sequences for Homo sapiens. A forward primer (5'-CCACCGGTGGTCTCAAAGATGAGCAGCGGCAACAGC-3') and a reverse primer (5'-TCGATGGAAGCTTGGCCTGACAGGCCTCAGCTGATCTTCTTGAACAGCCG-3') were used to add a 21 bp overlap with the C-terminus of the hsEH CDS at the 5' end, and a 29 bp region at the 3' end containing a TGA stop codon and overlap with the pSBtet-Bla vector backbone near the SfiI site downstream of the MCS. The reaction conditions were: initial denaturation at 98 °C for 1 min; 25 cycles of 98 °C for 20 s, 66 °C for 20 s, and 72 °C for 30 s; with a final extension at 72 °C for 5 min. To generate the final hsEH_aa1-aa555_Linker-HiBiT insert, PCR 2 was performed as a fusion PCR using both the PCR 1 product and the published sEH construct from Hahn et al. (10.1002/cmdc.201100433) as templates. The same reverse primer as in PCR 1 and a forward primer (5'-TACCCTCGAAAGGCCTCTGAGGCCACCATGACGCTGCGCGC-3') were used. This forward primer introduced a 27 bp overlap with the 5' SfiI site region of pSBtet-Bla to facilitate Gibson Assembly. Reaction conditions were: 98 °C for 1 min; 25 cycles of 98 °C for 40 s, 65 °C for 20 s, and 72 °C for 1 min 30 s; with a final extension at 72 °C for 5 min. For PCR 3, the entire pSBtet-Bla vector backbone was

amplified using the forward primer (5'-TGAGGCCTGTCAGGCCAAGCTTCCATCGA-3') and reverse primer (5'-CATGGTGGCCTCAGAGGCCTTTCGAGGGTA-3'). Cycling conditions were: 98 °C for 1 min; 25 cycles of 98 °C for 20 s, 66 °C for 20 s, and 72 °C for 5 min; with a final extension at 72 °C for 8 min. The final hsEH_aa1-aa555_Linker-HiBiT_pSBtet construct was assembled using the NEBuilder® HiFi DNA Assembly Cloning Kit as per the manufacturer's instructions. The 5 µl assembly reaction contained approximately 40 ng of insert DNA (PCR 2) and 52 ng of vector backbone (PCR 3). For the final construct hsEH_aa1-aa555_Linker-HiBiT_pSB-hPGK, the hPGK promoter fragment was generated in PCR 4 using pLKO.1-puro-shNM as template with the forward primer (5'-GGTCCGCTATCTAGACGAGTAGCAGAGATCCACTTTGGCC-3') and reverse primer (5'-GGCAAAAGAGTTGGAATTGGCCCTGGGGAGAGAGGTCGG-3'). Reaction conditions: 98 °C for 30 s; 25 cycles of 98 °C for 10 s, 62 °C for 30 s, and 72 °C for 30 s; final extension at 72 °C for 4 min. In PCR 5, the intermediate construct (hsEH_aa1-aa555_Linker-HiBiT_pSBtet) was amplified with forward primer (5'-GCCAATTCCAACTCTTTTGCCTTATACC-3') and reverse primer (5'-ACTCGTCTAGATAGCGGACC-3') to generate a linearized plasmid excluding the tet-On promoter region. Conditions were: 98 °C for 2 min; 30 cycles of 98 °C for 40 s, 62 °C for 20 s, and 72 °C for 5 min; with a final extension at 72 °C for 8 min. The final construct was assembled using the NEBuilder® HiFi DNA Assembly Cloning Kit as recommended, with 5 µl assembly reactions containing ~13 ng of the hPGK promoter fragment (PCR 4) and ~66 ng of the linearized vector (PCR 5). HeLa cells were seeded two days prior to transfection in 6-well plates at a density of 4 × 10^5 cells per well in 3 mL of DMEM (1X) medium with phenol, supplemented with 10% Corning® Fetal Bovine Serum, 100 U/mL penicillin and 100 µg/mL streptomycin, and 1 mM sodium pyruvate. This medium is hereafter referred to as DMEMsup. On the day of transfection, each well was washed with 2 mL PBS and then incubated with 1 mL Opti-MEM™ medium. Transfection was performed using the Lipofectamine™ 3000 protocol with minor adjustments. 500 µL of Opti-MEM™ was mixed with 4.2 µg of hsEH_aa1-aa555_Linker-HiBiT_pSB-hPGK plasmid, 0.2 µg of pSB100x transposase plasmid, and 8.8 µL of P3000™ reagent. Separately, 500 µL of Opti-MEM™ was combined with 4 µL of Lipofectamine™ 3000. The two mixtures were then combined and incubated at rt for 10 min before being added to a single well of the 6-well plate. Cells were incubated with the transfection mixture at 37 °C and 5% CO_2 for 4 h, after which the medium was replaced with 2 mL of DMEMsup. 24 h post-transfection, cells were transferred to a 75 cm² tissue culture flask and cultured in selection medium, consisting of 50 mL DMEMsup supplemented with 25 µL of a 10 mg/mL blasticidin stock solution (final concentration: 5 µg/mL). After 4 days under selection, cells were transferred to a 175 cm² flask and maintained in selection medium for an additional 10–14 days to establish stably transfected cell lines.

Cell Viability Assay

The effect of the compounds on cell viability for BRD4 was determined using the CellTiter-Glo® 2.0 Cell Viability Assay (Promega: G9241) following manufacturer's protocol. 10 µL of HEK293T cells were seeded at a cell density of $2 \cdot 10^5$ cells/mL into individual wells of a white 384-well plate (Greiner: 781207) and the cells were allowed to equilibrate for 1 h at 37 °C and 5% CO_2. After equilibration, the compounds were titrated at various concentrations using an Echo acoustic dispenser (Labcyte) and the cells were incubated for 24 h at 37 °C and 5% CO_2. Equal volume of CellTiter-Glo® 2.0 reagent (10 µL) was added to each well and cells were incubated for 10 min at rt. Filtered luminescence was measured on a PHERAstar plate reader (BMG Labtech) and data was evaluated using GraphPad Prism 9 software employing a normalized curve it with the following equation: $Y = Bottom + (Top-Bottom)/(1 + 10^{(LogEC50-X)*HillSlope})$.

The effect of the compounds on cell viability for AurkA was determined using the alamarBlue™ Cell Viability Assay. For the assay, cells were plated in 96-well formats and exposed to the specified concentrations of each compound or to the vehicle control. After a 24 hour treatment period, the alamarBlue™ reagent was added. Fluorescence signals (550 nm excitation, 600 nm emission) or absorbance readings (570 nm with a 600 nm reference) were subsequently recorded using a Tecan Spark multimode plate reader.

Solubility Limit Assay

The aqueous solubility of compounds P1–P8 was evaluated using an assay that detects changes in light absorption resulting from compound precipitation and light scattering. A dilution series of each compound was prepared in DMSO (range: 100 µM – 0.5 µM), and 0.5 µL of the corresponding stock solution was dispensed into a transparent, flat-bottom 96-well plate (in triplicate). Subsequently, 99.5 µL of DBPS buffer (Gibco™ Dulbecco's Phosphate Buffered Saline, 1×, pH 7.0–7.3, Thermo Fisher Scientific), supplemented with 0.01% aqueous Triton X-100, was added to each well, yielding a final DMSO concentration of 0.5%. Absorbance at 600 nm was measured for each well using a Tecan Spark Multimode Plate Reader. The absorbance values of the compound dilution series were compared with the DMSO vehicle control in Microsoft Excel, and the point at which absorbance increased was used to determine the solubility limit.

Microsomal Stability assay in Liver Microsomes

The solubilized test compound (5 µL, final concentration 10 µM) was pre-incubated at 37 °C in 432 µL of phosphate buffer (0.1 M, pH 7.4) together with a 50 µL NADPH regenerating system (30 mM glucose 6 phosphate, 4 U/mL glucose 6 phosphate dehydrogenase, 10 mM NADP, 30 mM $MgCl_2$). After 5 min,

the reaction was started by the addition of 13 µL of microsome mix in a shaking water bath at 37 °C. For the experiments, liver microsomes from Sprague-Dawley rats (Gibco™; 20 mg protein/mL) were used. The reaction was stopped by adding 500 µL of ice cold methanol at 0, 15, 30, and 60 min (N=2). The samples were centrifuged at 10000g for 5 min at 4 °C, and the test compound was quantified from the supernatants via HPLC: a 1260 Infinity II LC System consisting of the multisampler G7167A, the column compartment G7116A, the multicolumn thermostat G7116A, the flexible pump G7104C and the diode array detector HS G7117C by the company Agilent Technologies was used for this purpose. An ACE UltraCore Super C18 column (150 x 3.0 mm) from Avantor was used as the stationary phase and a gradient of H_2O and ACN with 0.1% formic acid served as the mobile phase. UV detection took place at wavelengths of 254, 280 and 320 nm; injection volume 10 µL. The following gradient was used: 0 min: 5% B - 2 min: 80% B – 5 min: 95% B - 7 min: 95% B (flow rate of 0.6 mL/min). Control samples were used to check the test compound's stability in the reaction mixture: controls were examined without NADPH, which is needed for the enzymatic activity of the Microsomes, after 0 and 60 min of incubation. The amounts of the test compound were quantified by an external calibration curve.

NMR Spectra and LC-MS Spectra, related to compounds in Figure 4G

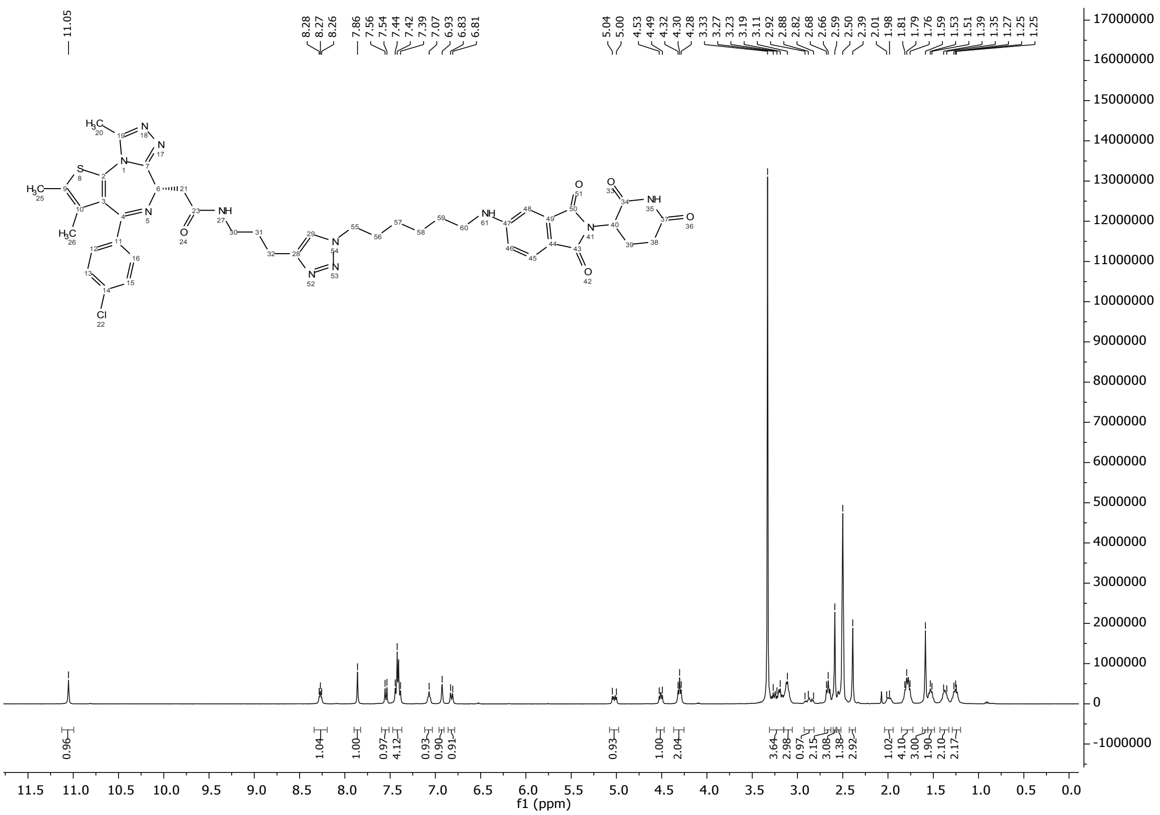

^{1}H NMR spectrum of **P1**, related to compound **P1** in Figure 4G

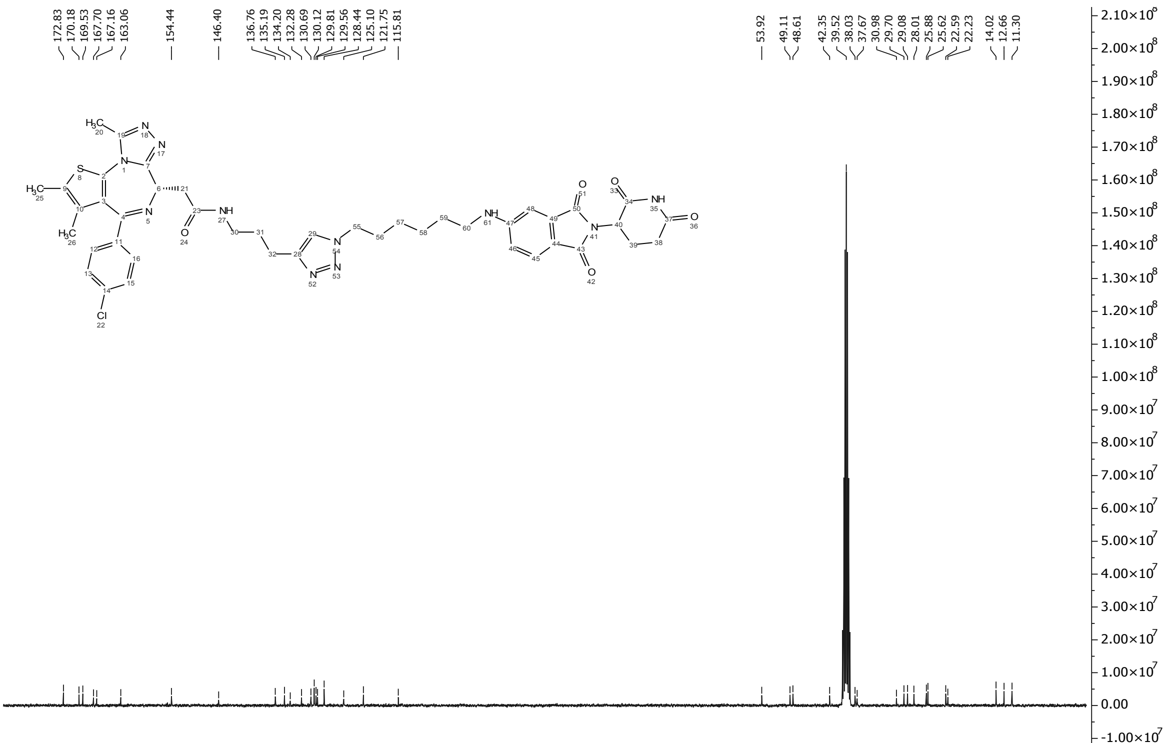

^{13}C NMR spectrum of **P1**, related to compound **P1** in Figure 4G

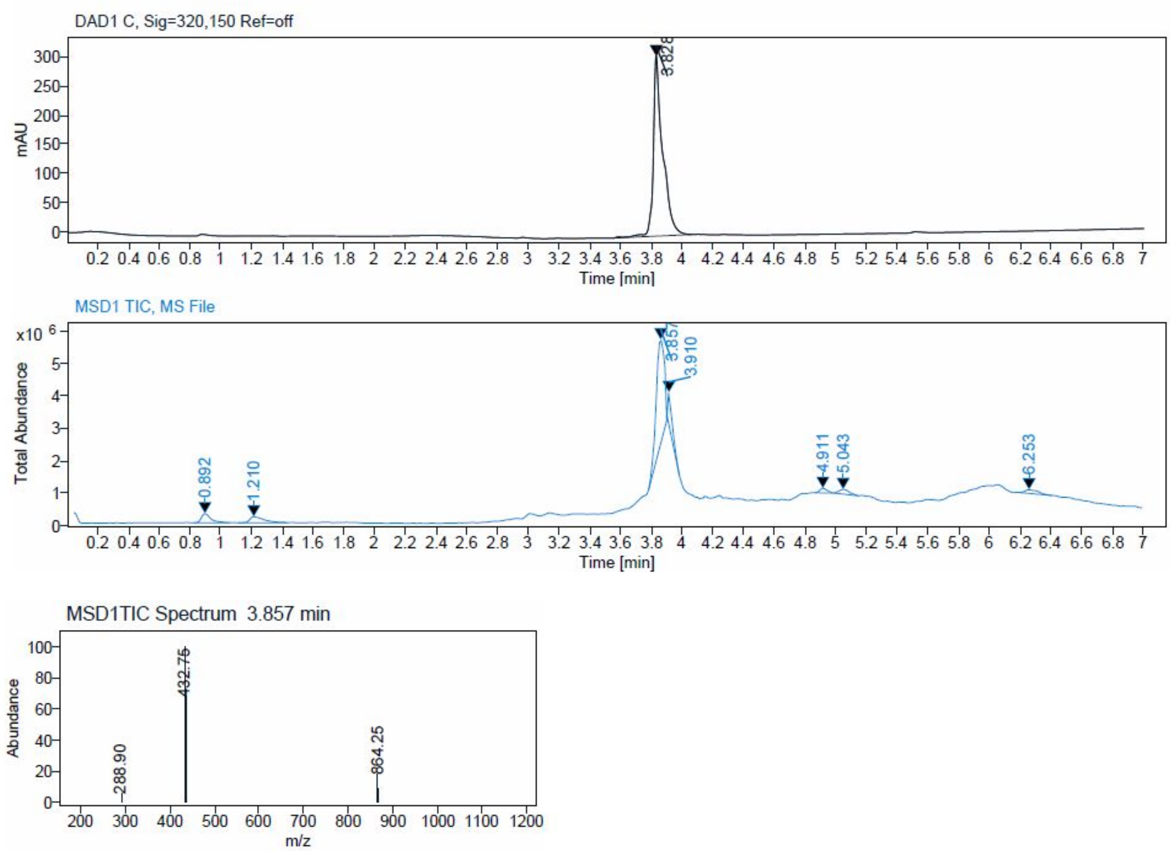

LC-MS spectrum of **P1**, related to compound **P1** in Figure 4G

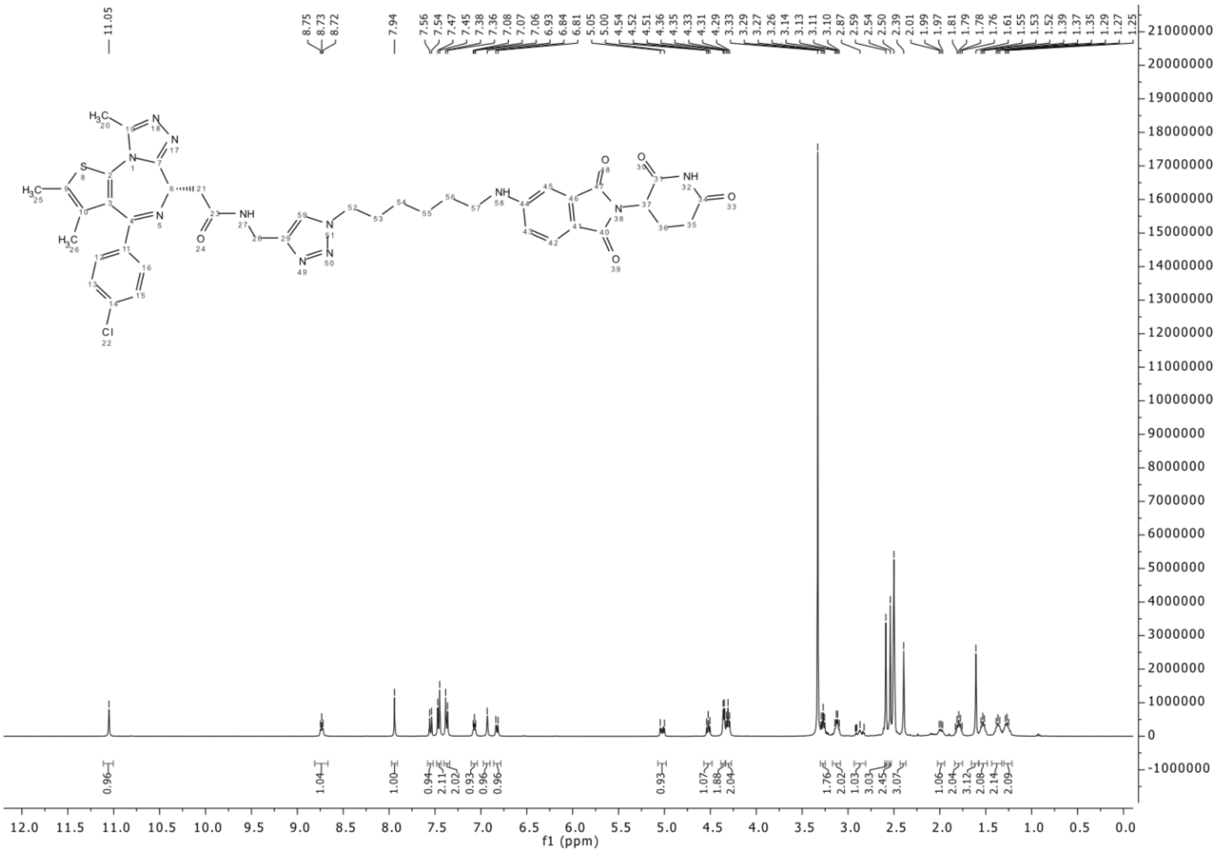

^{1}H NMR spectrum of **P2**, related to compound **P2** in Figure 4G

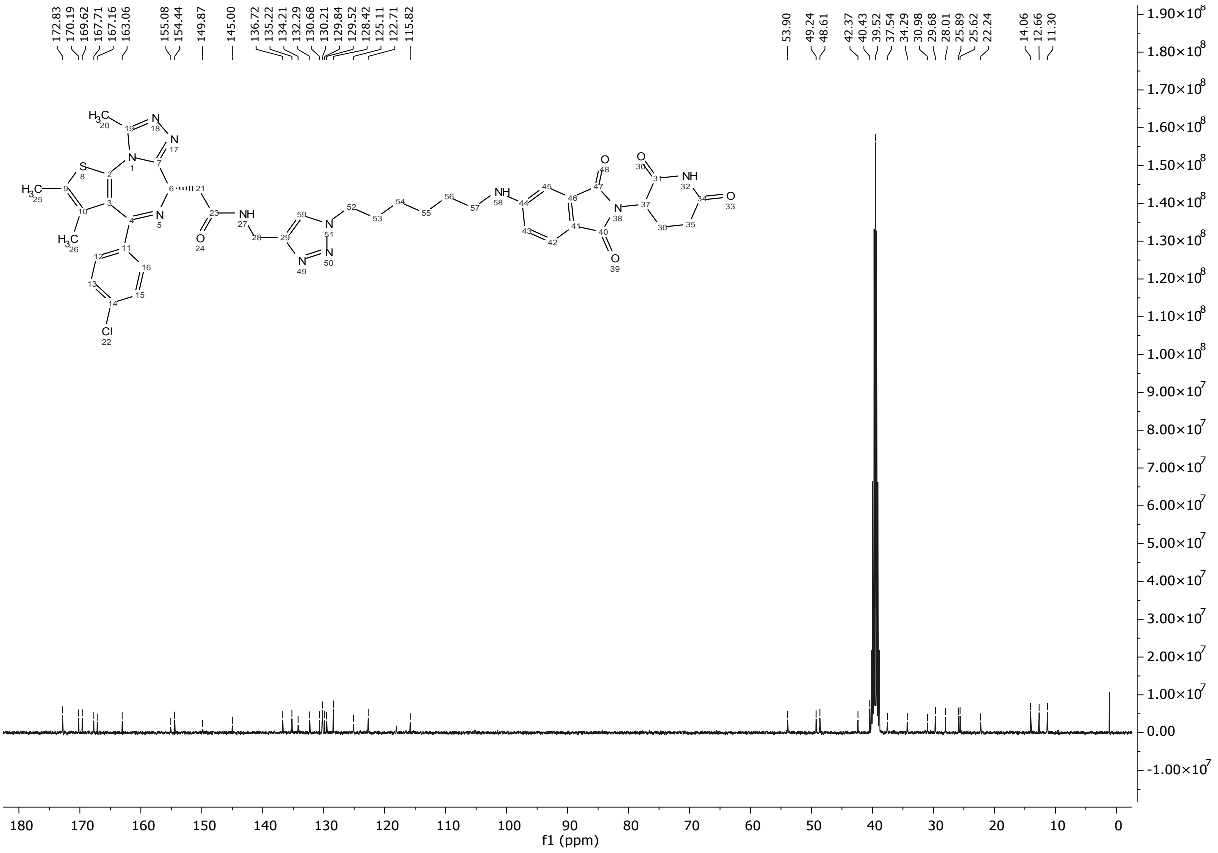

^{13}C NMR spectrum of **P2**, related to compound **P2** in Figure 4G

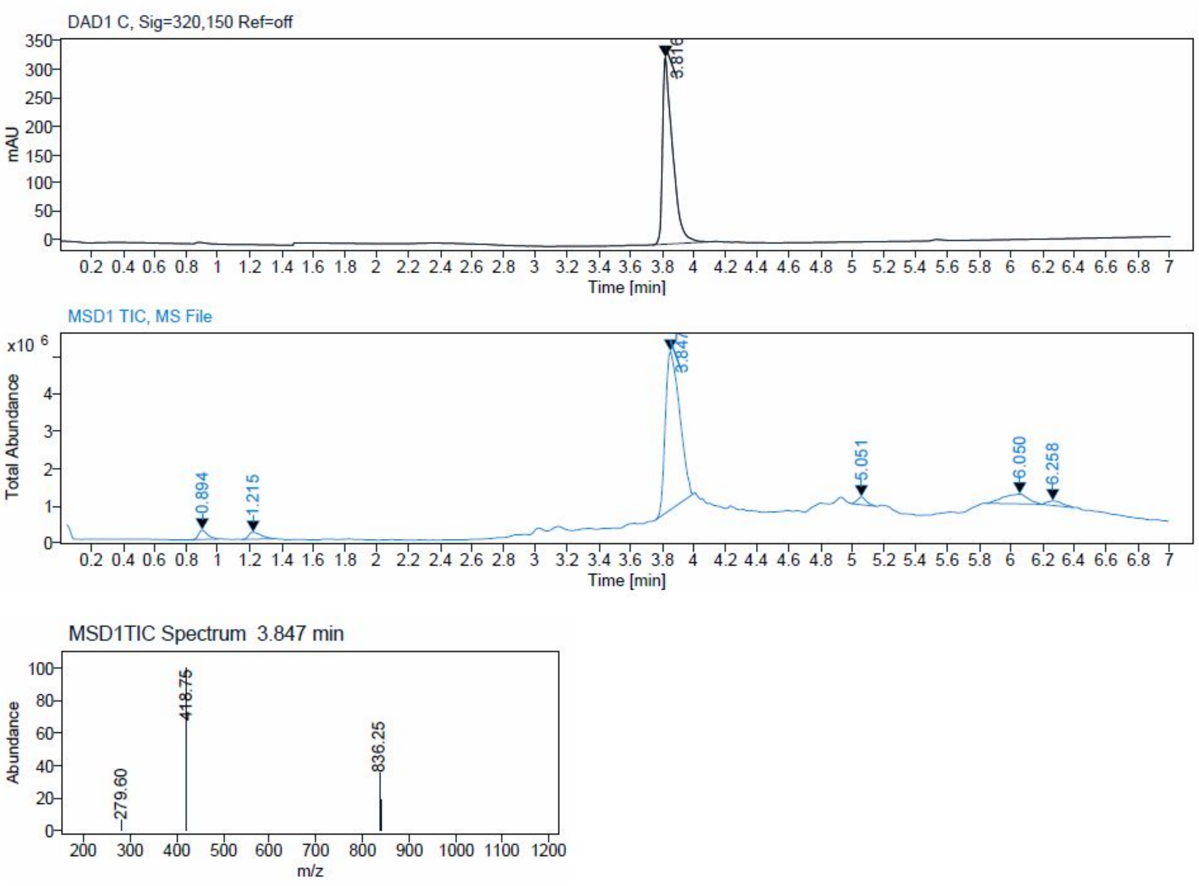

LC-MS spectrum of **P2**, related to compound **P2** in Figure 4G

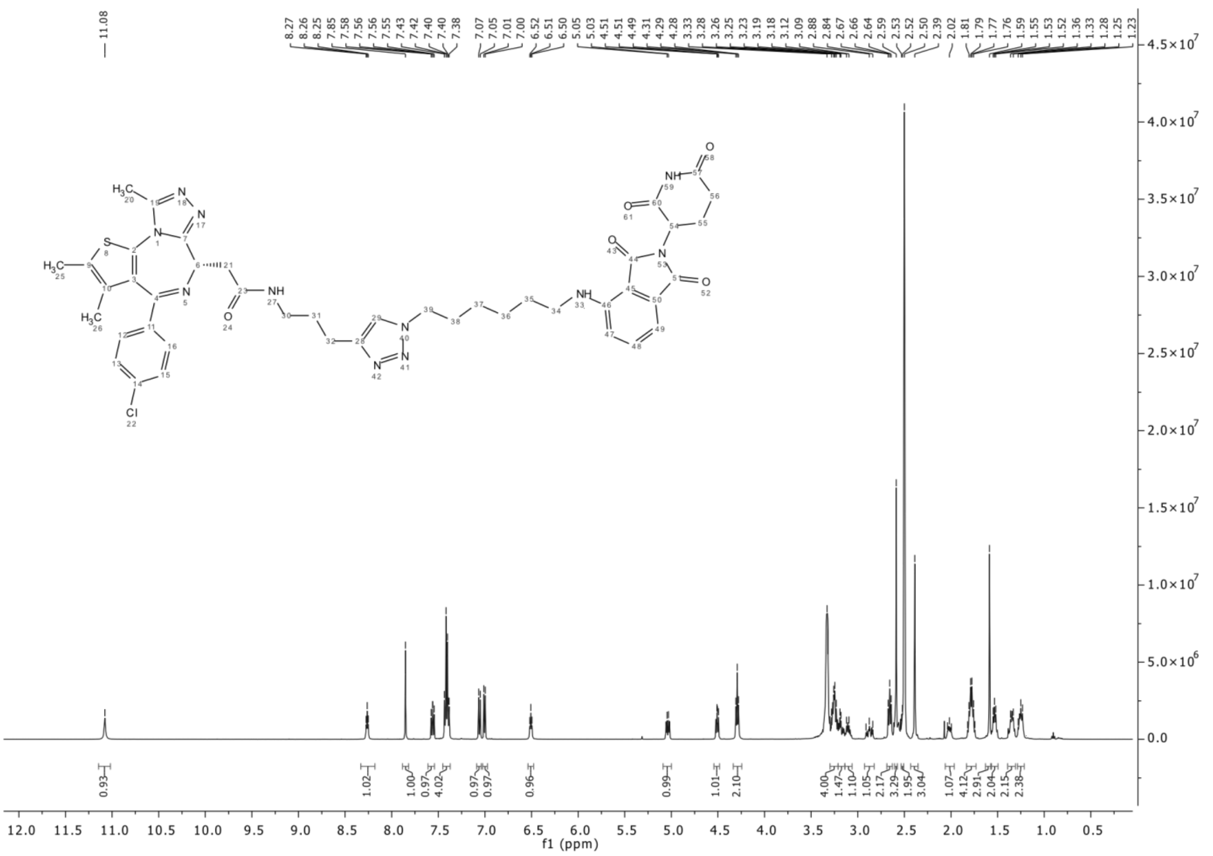

^{1}H NMR spectrum of **P3**, related to compound **P3** in Figure 4G

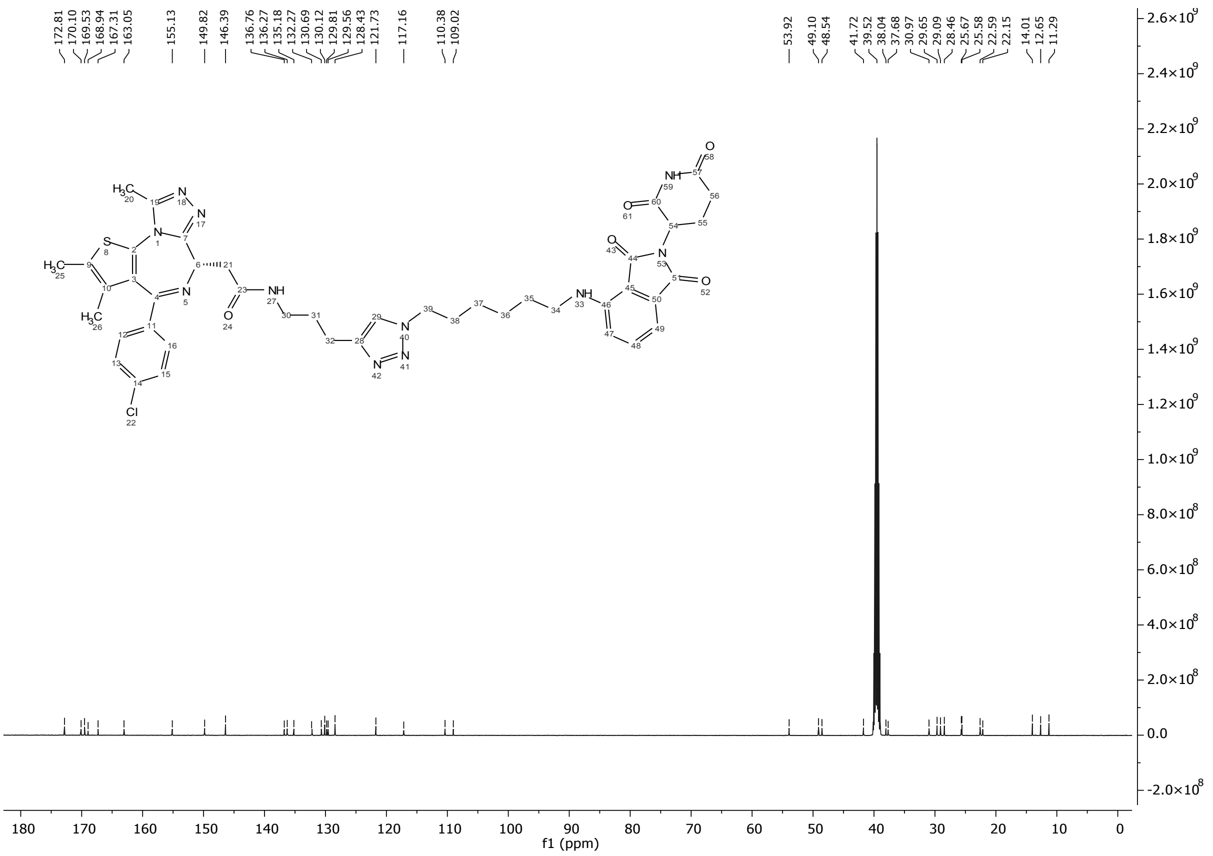

^{13}C NMR spectrum of **P3**, related to compound **P3** in Figure 4G

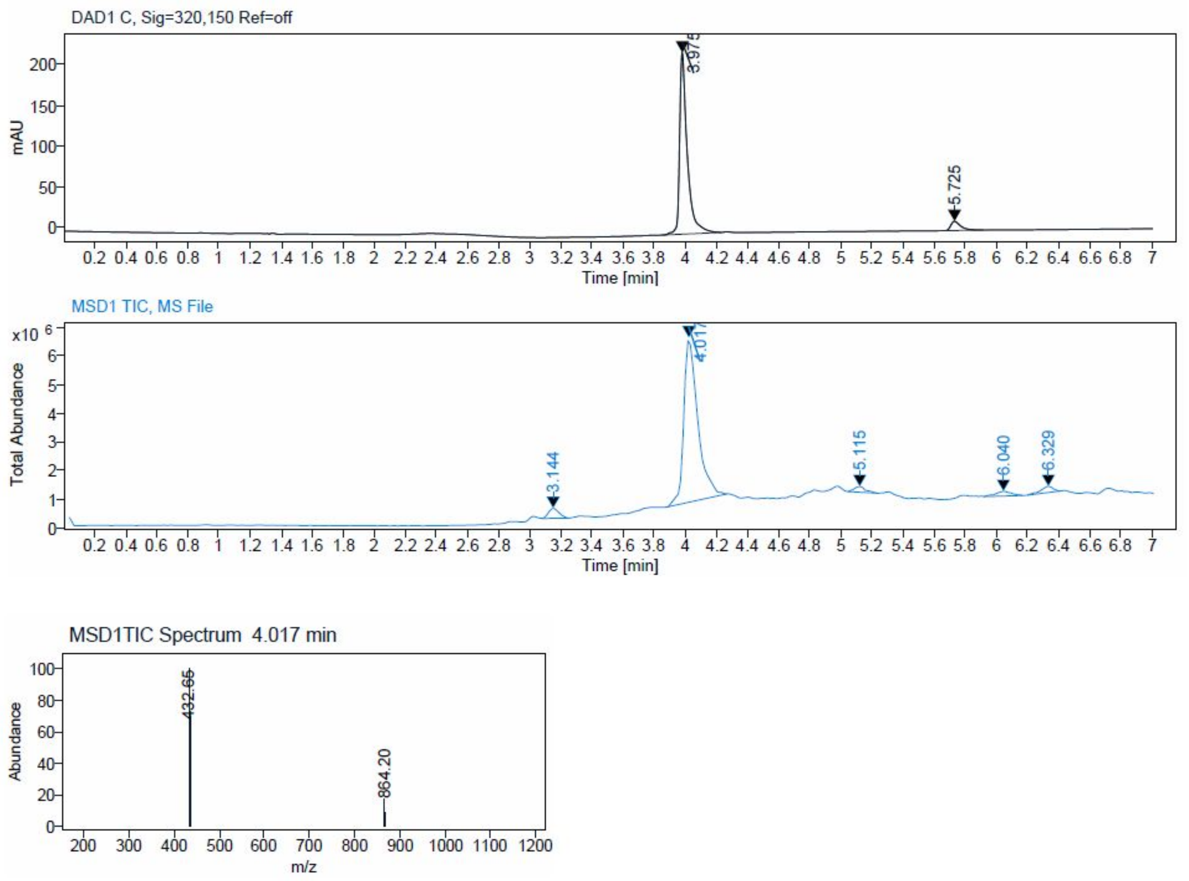

LC-MS spectrum of **P3**, related to compound **P3** in Figure 4G

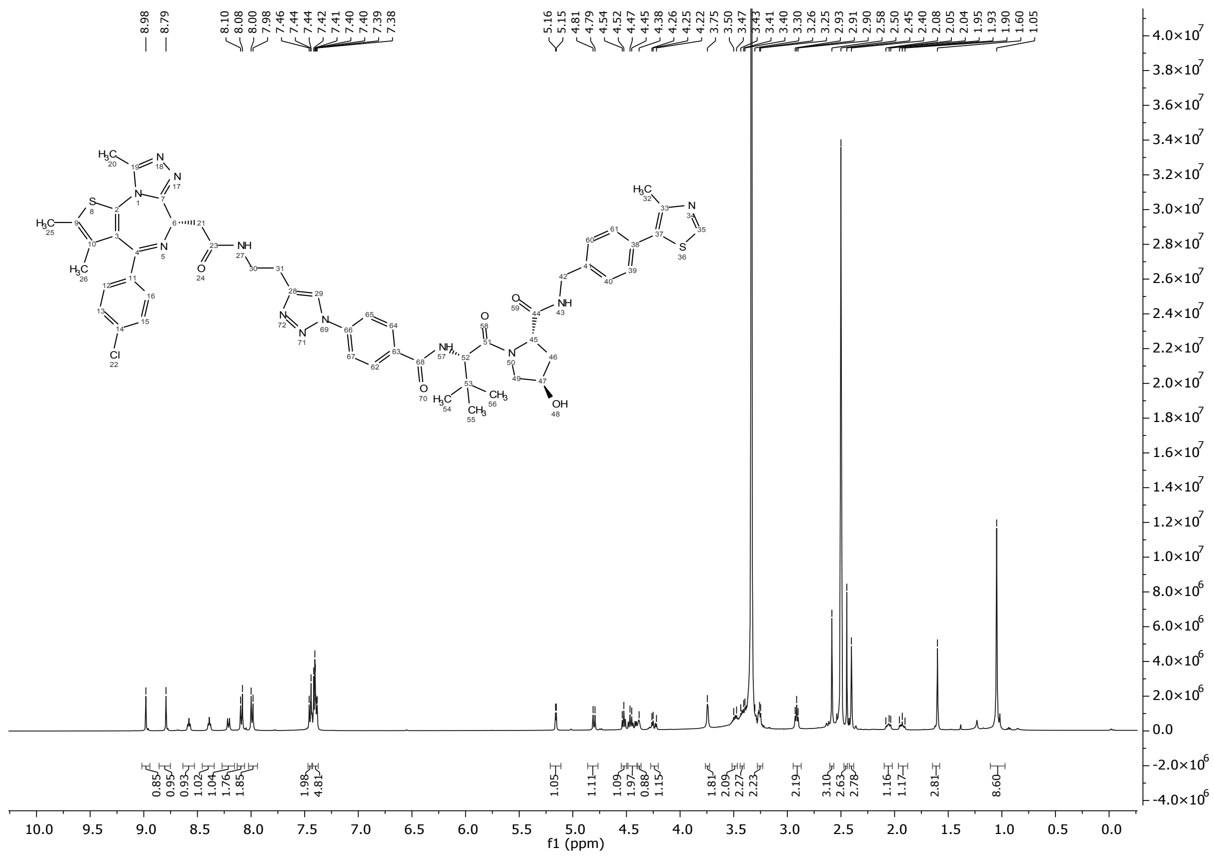

^{1}H NMR spectrum of **P4**, related to compound **P4** in Figure 4G

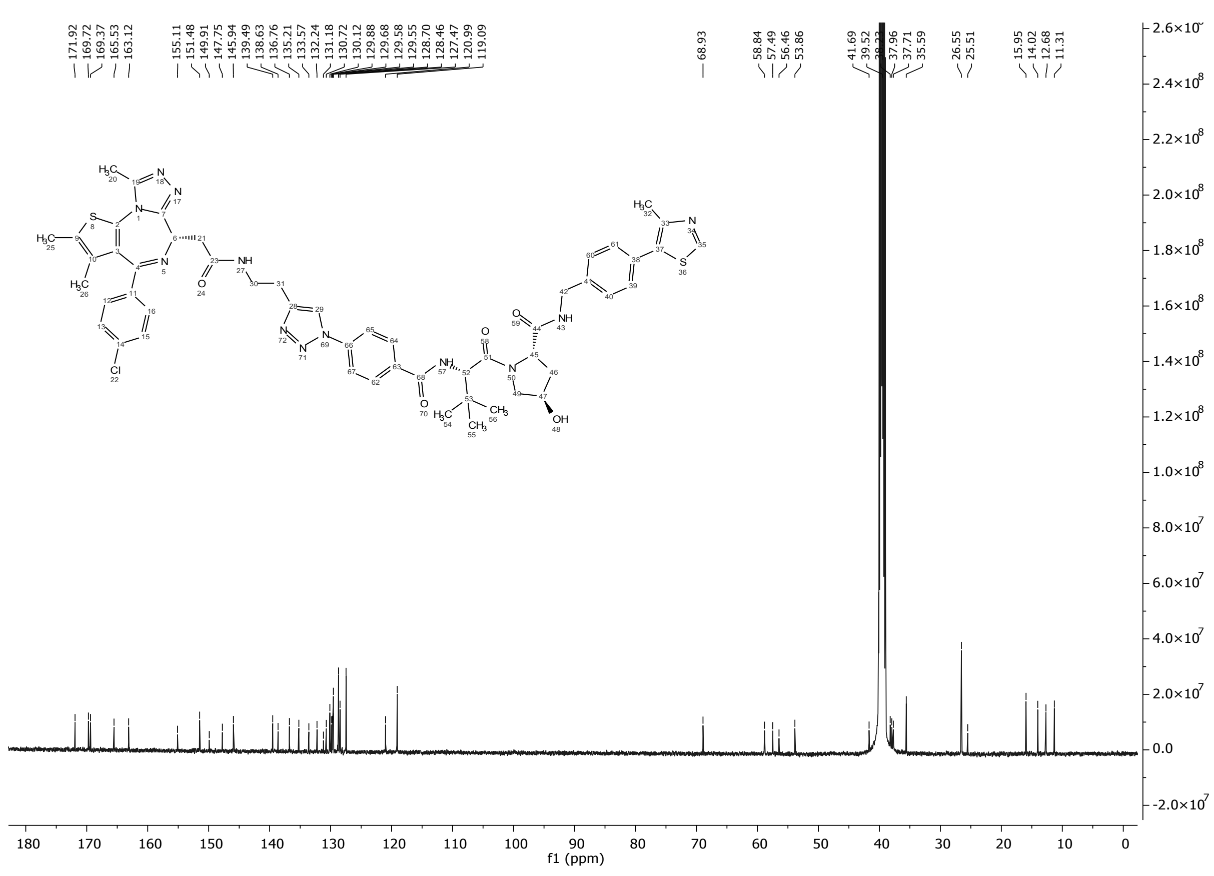

^{13}C NMR spectrum of **P4**, related to compound **P4** in Figure 4G

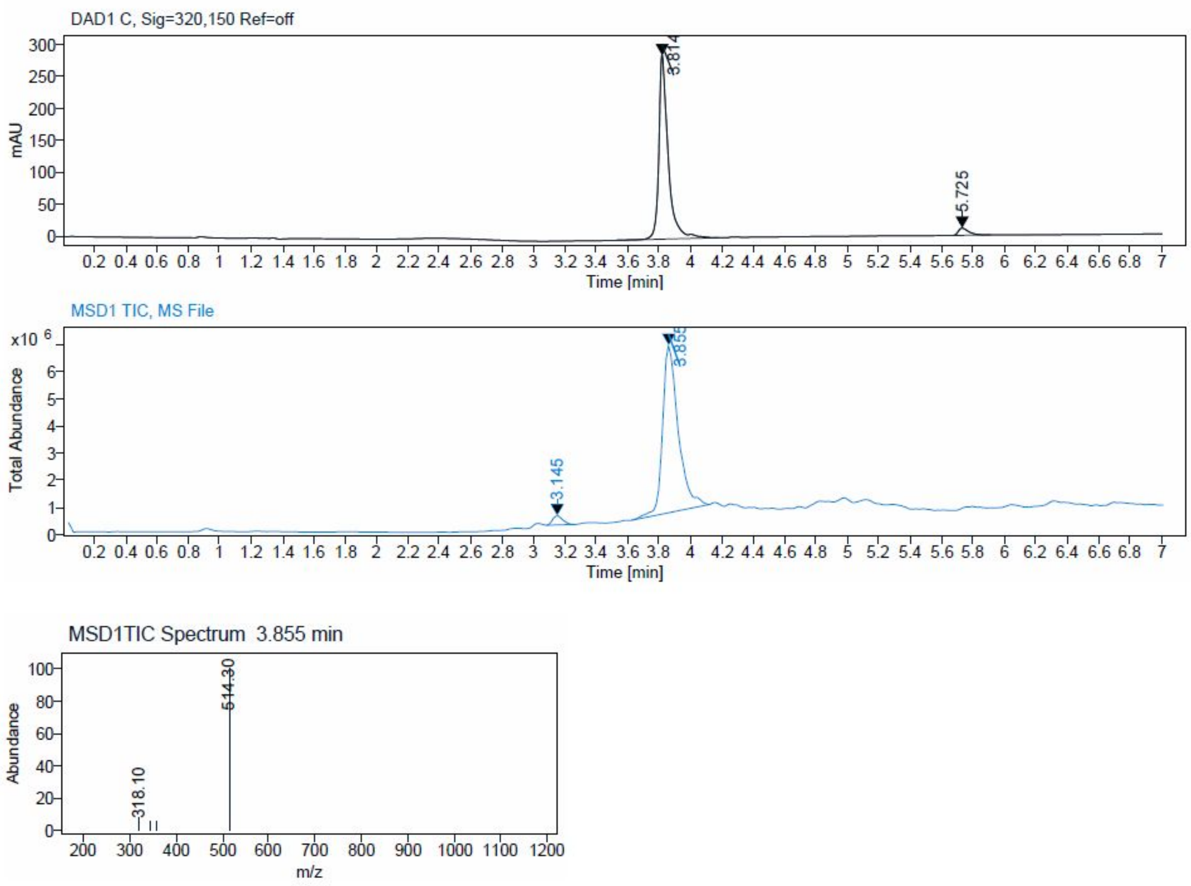

LC-MS spectrum of **P4**, related to compound **P4** in Figure 4G

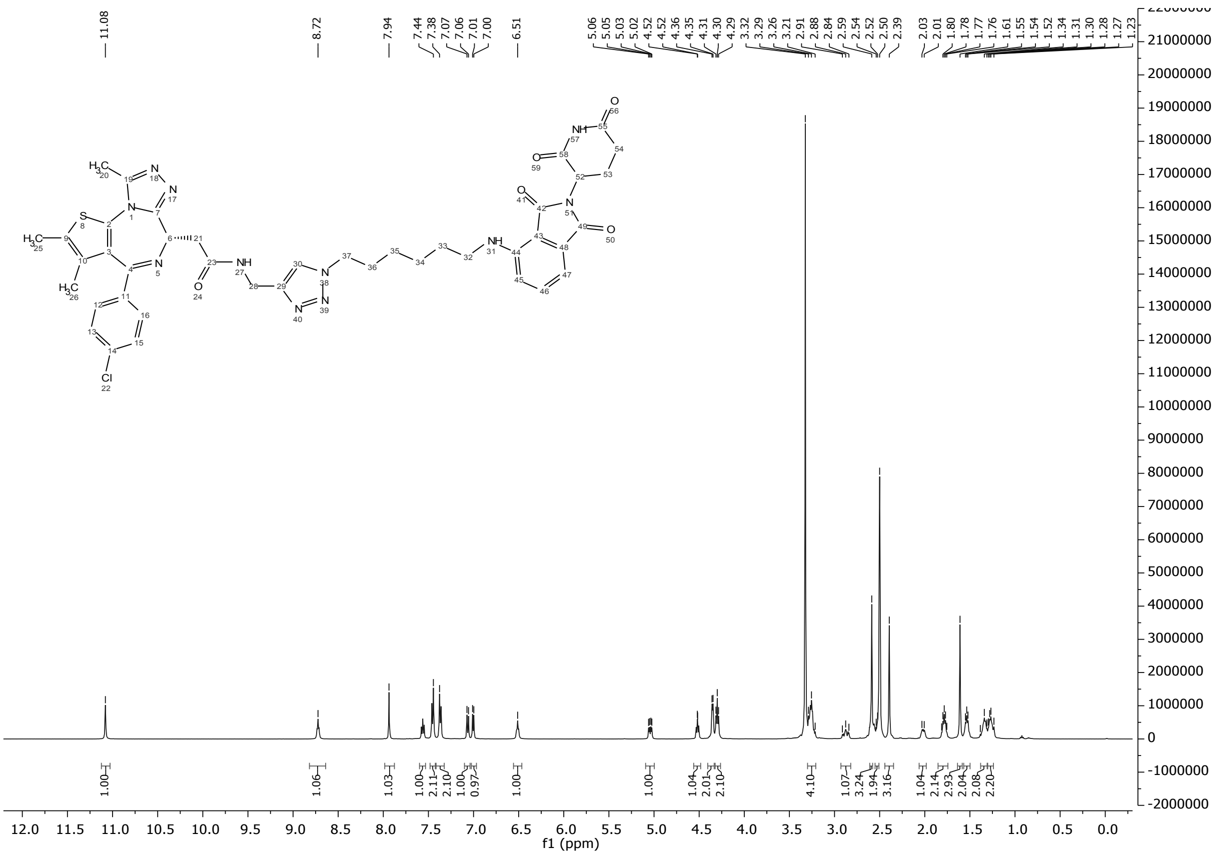

^{1}H NMR spectrum of **P5**, related to compound **P5** in Figure 4G

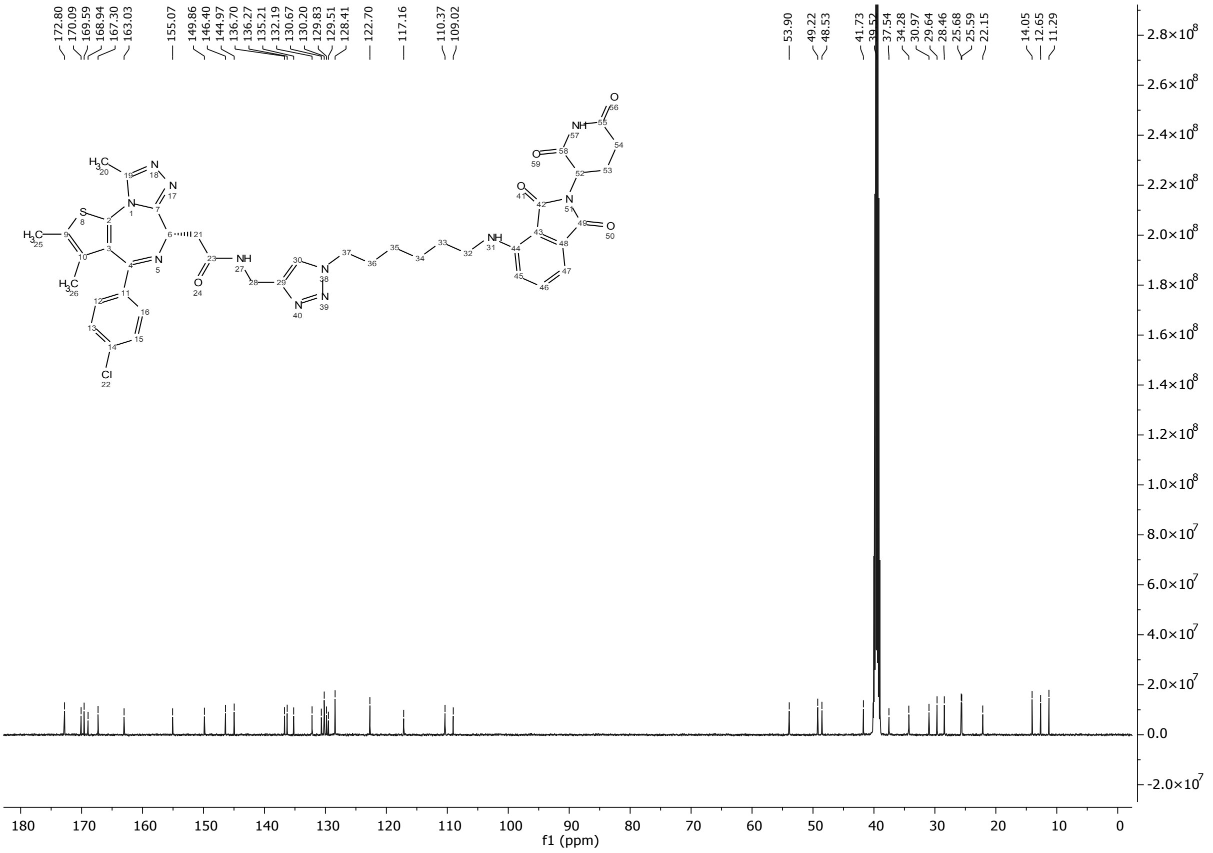

^{13}C NMR spectrum of **P5**, related to compound **P5** in Figure 4G

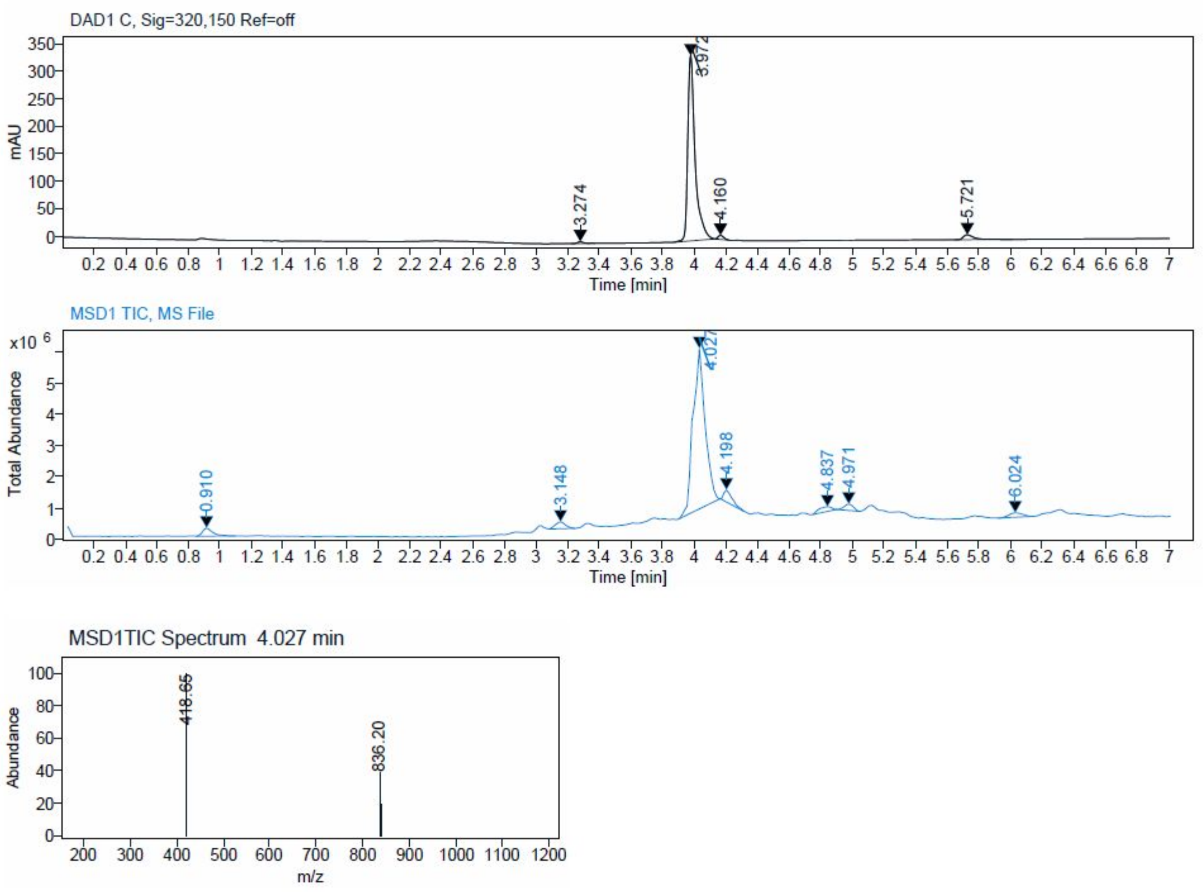

LC-MS spectrum of **P5**, related to compound **P5** in Figure 4G

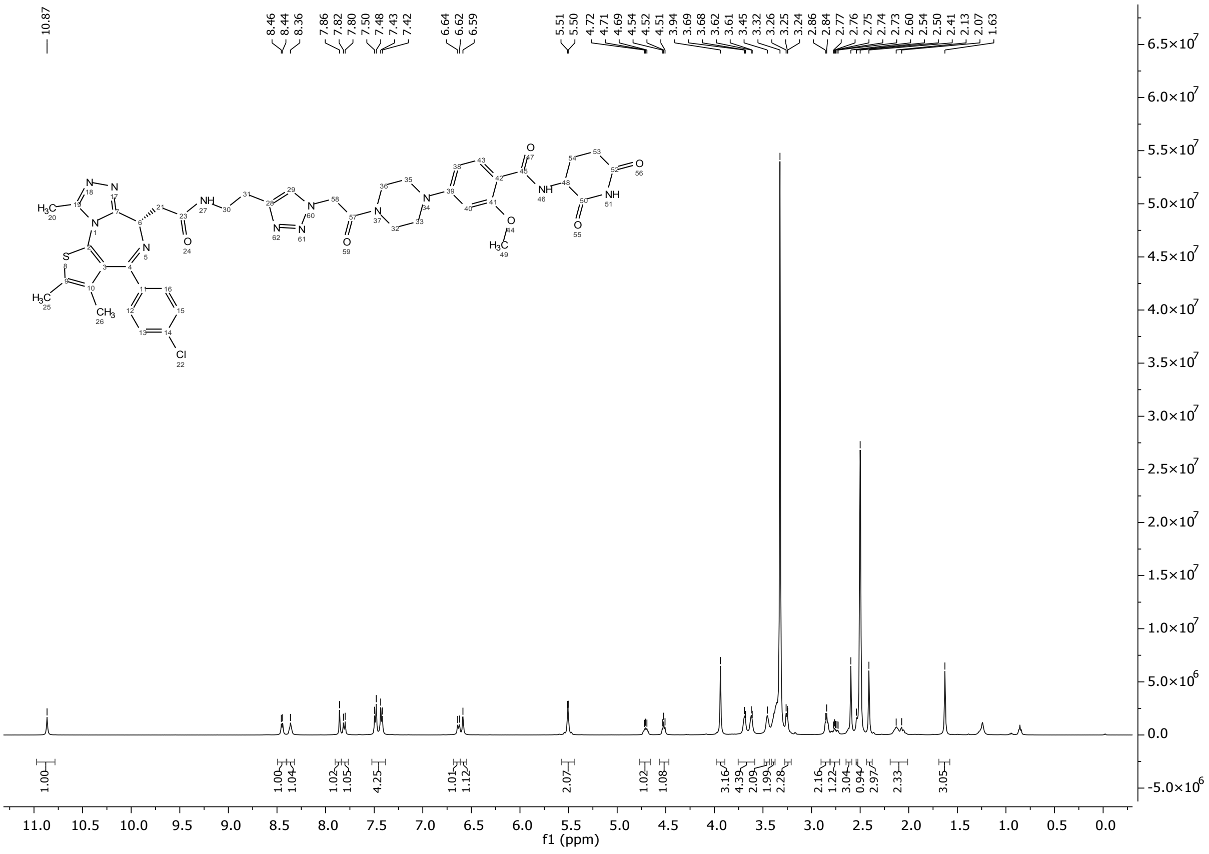

^{1}H NMR spectrum of **P6**, related to compound **P6** in Figure 4G

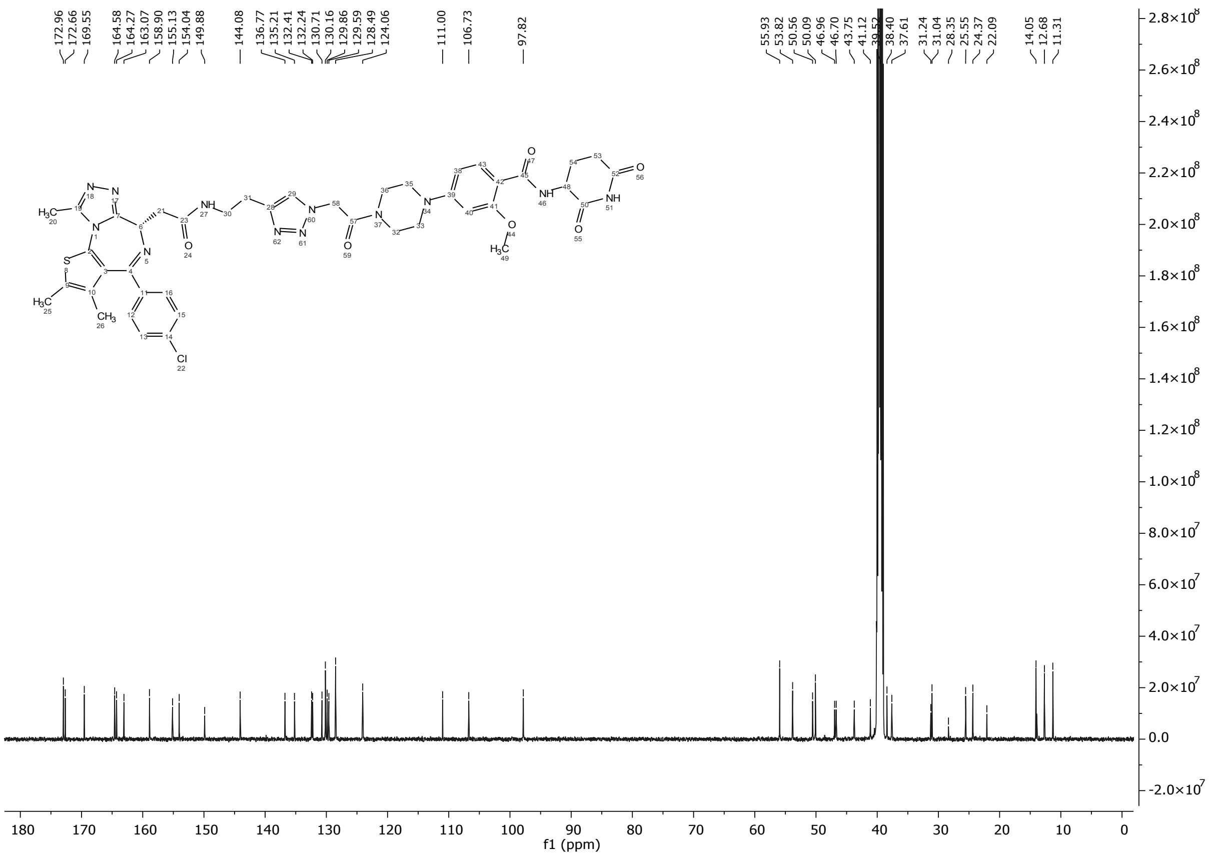

^{13}C NMR spectrum of **P6**, related to compound **P6** in Figure 4G

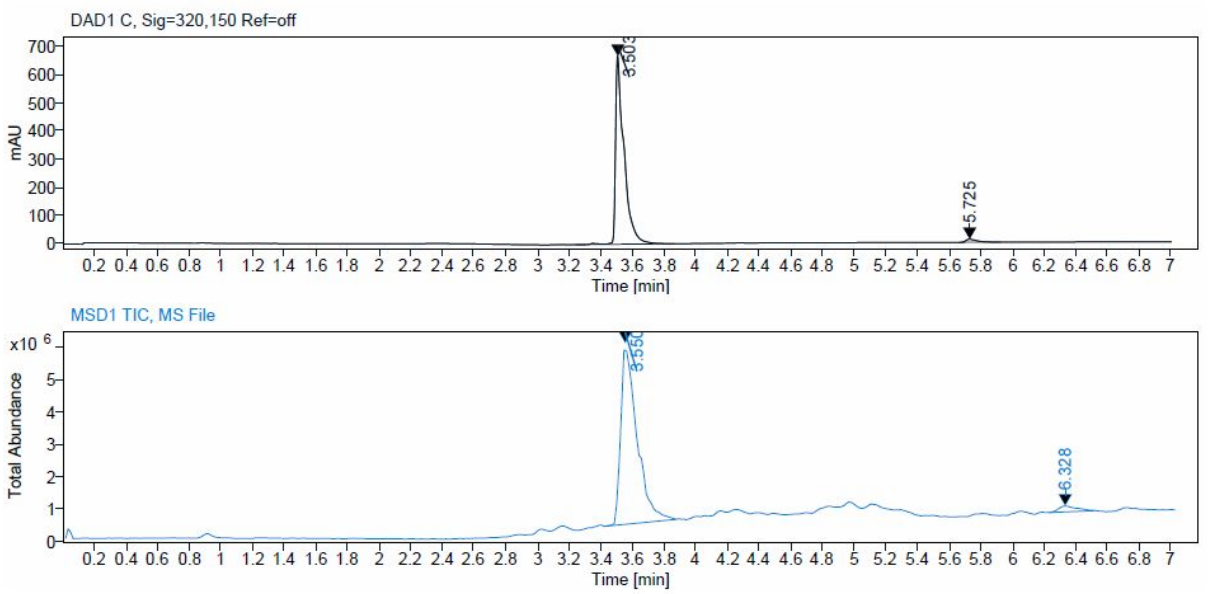

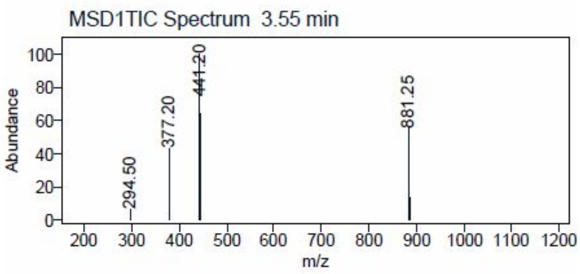

LC-MS spectrum of **P6**, related to compound **P6** in Figure 4G

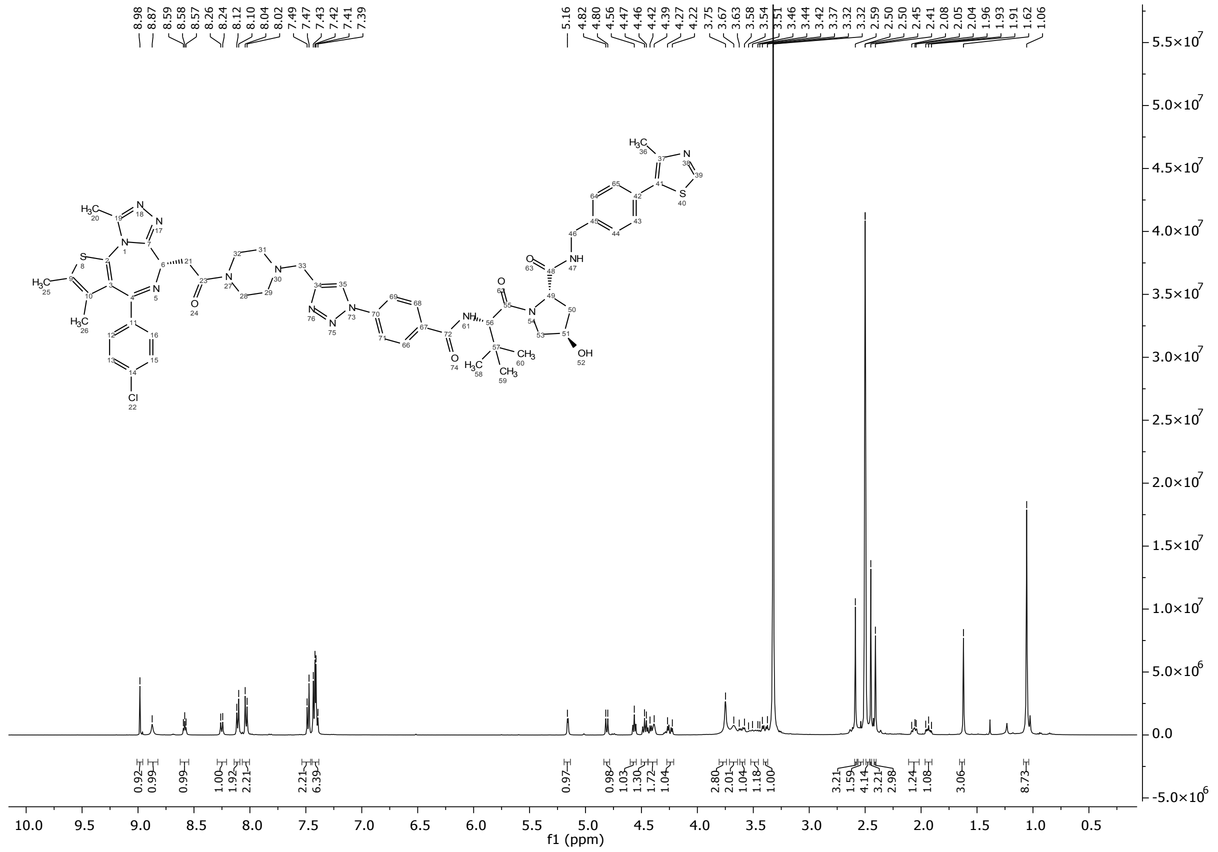

^{1}H NMR spectrum of **P7**, related to compound **P7** in Figure 4G

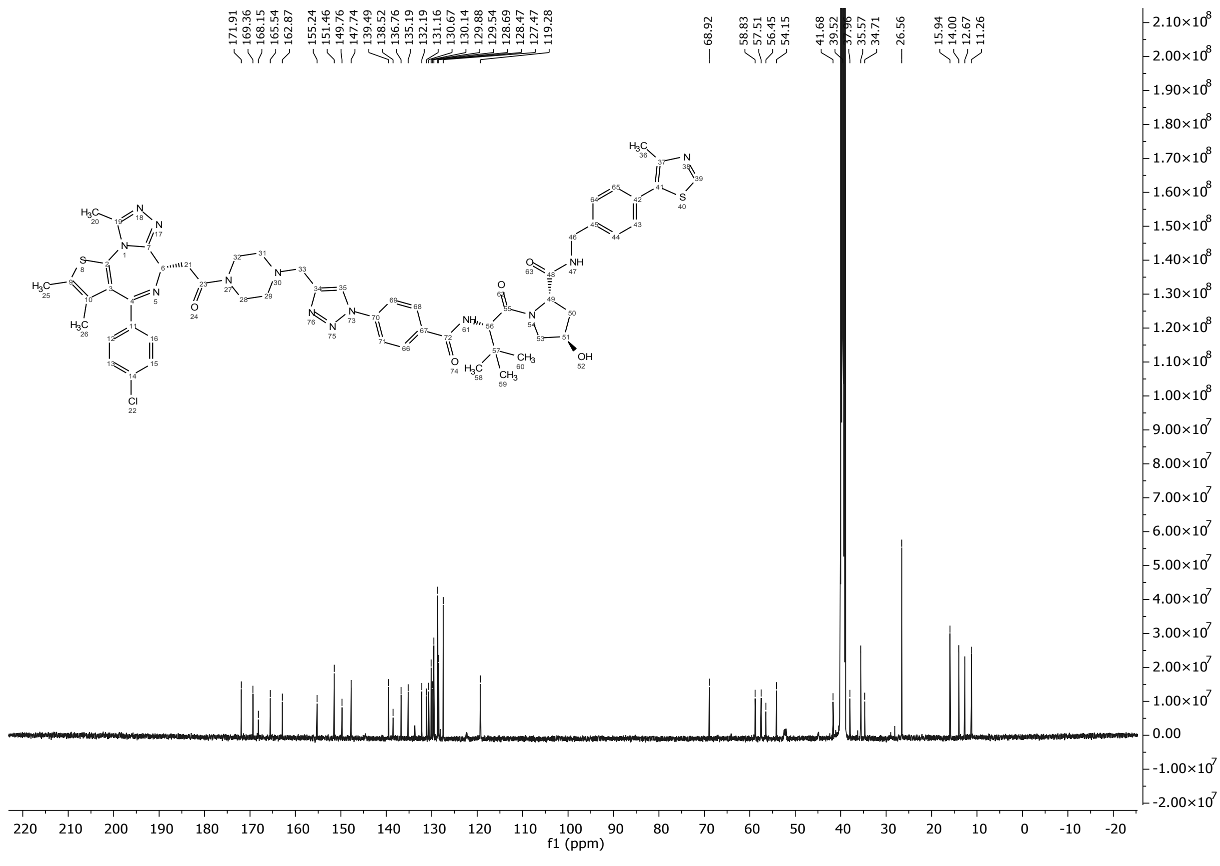

^{13}C NMR spectrum of **P7**, related to compound **P7** in Figure 4G

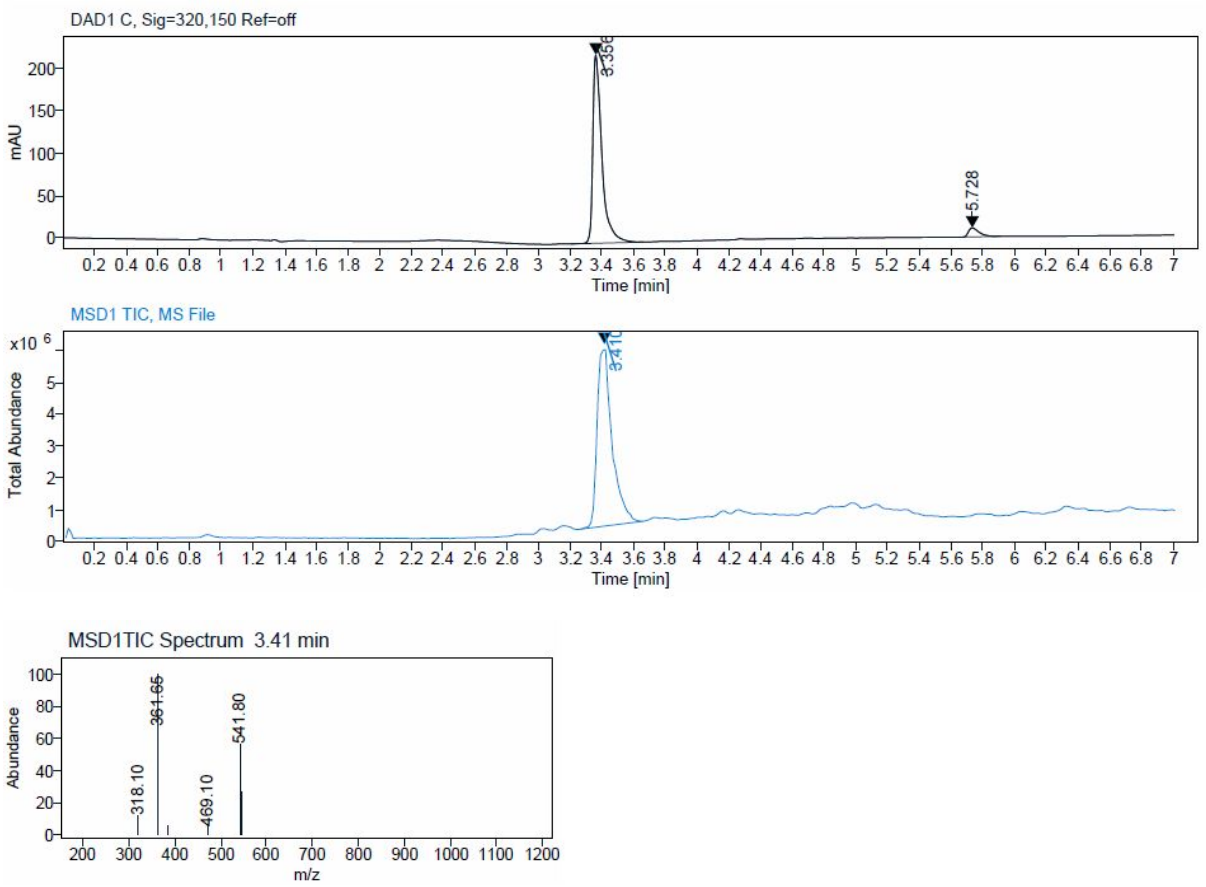

LC-MS spectrum of **P7**, related to compound **P7** in Figure 4G

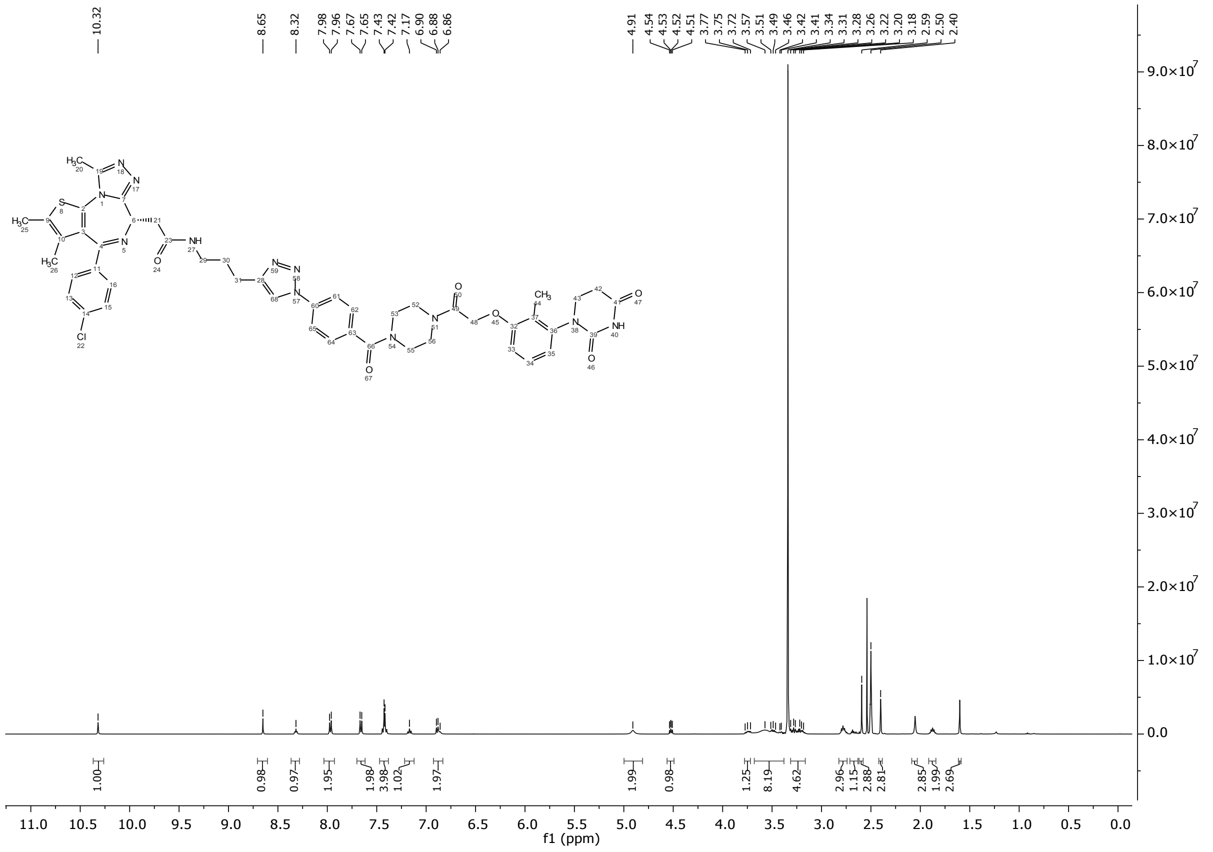

^{1}H NMR spectrum of **P8**, related to compound **P8** in Figure 4G

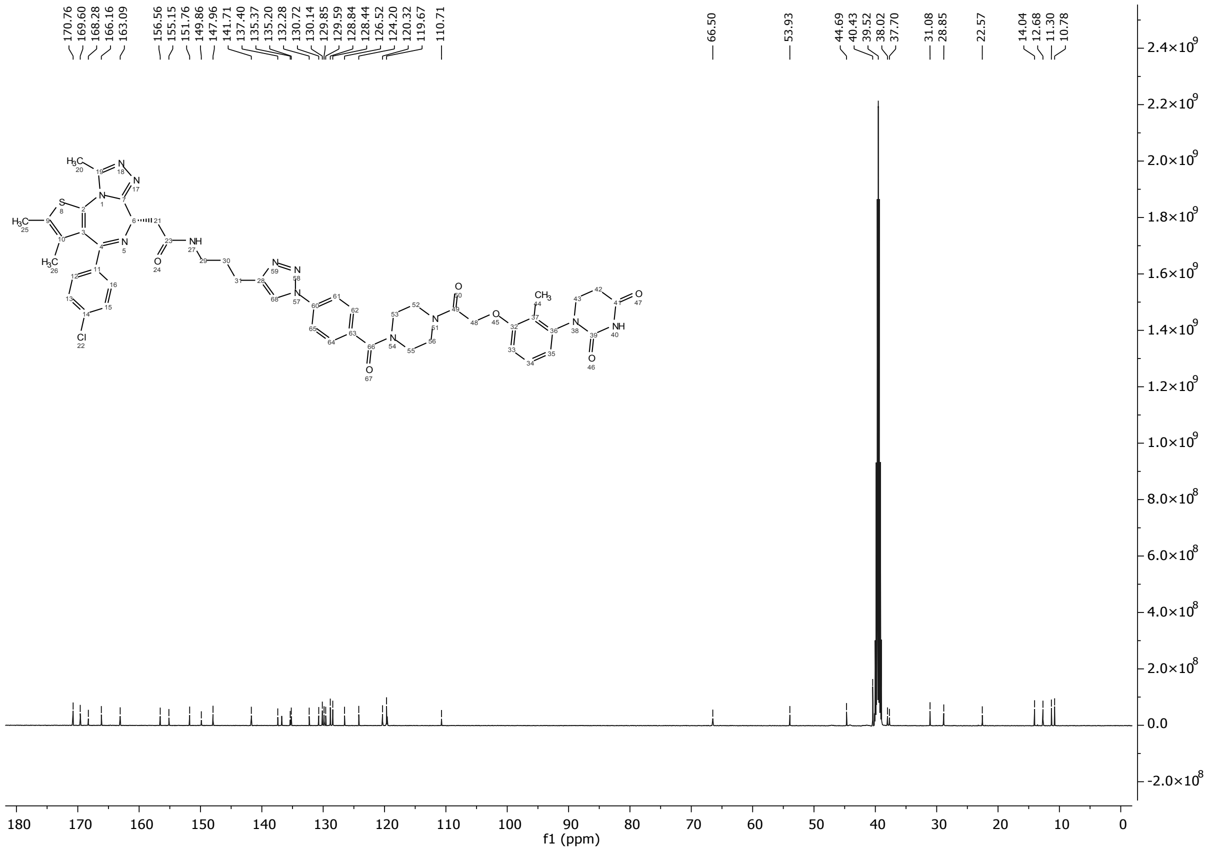

^{13}C NMR spectrum of **P8**, related to compound **P8** in Figure 4G

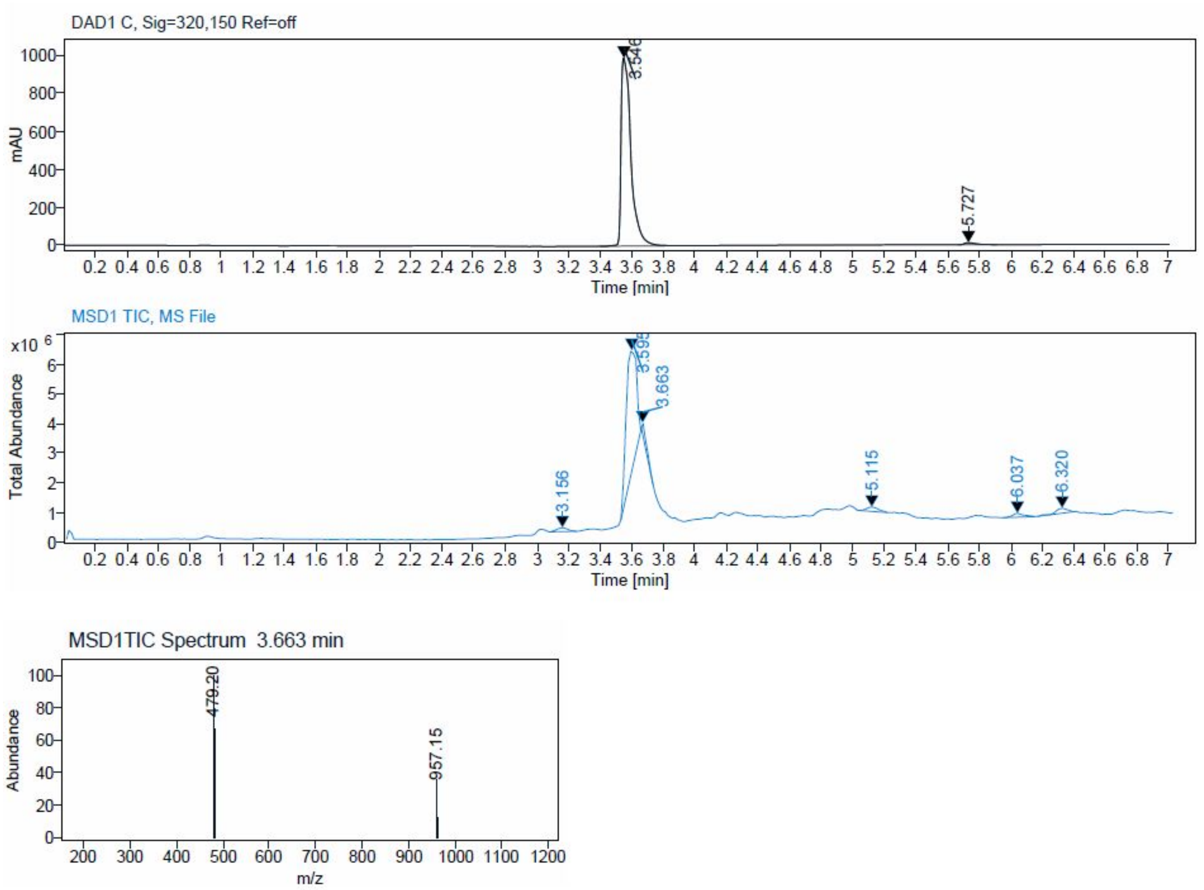

LC-MS spectrum of **P8**, related to compound **P8** in Figure 4G
